# A taxon-rich and genome-scale phylogeny of Opisthokonta

**DOI:** 10.1371/journal.pbio.3002794

**Published:** 2024-09-16

**Authors:** Hongyue Liu, Jacob L. Steenwyk, Xiaofan Zhou, Darrin T. Schultz, Kevin M. Kocot, Xing-Xing Shen, Antonis Rokas, Yuanning Li

**Affiliations:** 1 Institute of Marine Science and Technology, Shandong University, Qingdao, China; 2 Laboratory for Marine Biology and Biotechnology, Qingdao Marine Science and Technology Center, Qingdao, China; 3 Howards Hughes Medical Institute and the Department of Molecular and Cell Biology, University of California, Berkeley, Berkeley, California, United States of America; 4 Guangdong Laboratory for Lingnan Modern Agriculture, Guangdong Province Key Laboratory of Microbial Signals and Disease Control, Integrative Microbiology Research Centre, South China Agricultural University, Guangzhou, China; 5 Department of Neuroscience and Developmental Biology, University of Vienna, Vienna, Austria; 6 Department of Biomolecular Engineering, University of California, Santa Cruz, Santa Cruz, California, United States of America; 7 Monterey Bay Aquarium Research Institute, Moss Landing, California, United States of America; 8 University of Alabama, Department of Biological Sciences & Alabama Museum of Natural History, Tuscaloosa, Alabama, United States of America; 9 Institute of Insect Sciences and Centre for Evolutionary and Organismal Biology, Zhejiang University, Hangzhou, China; 10 Department of Biological Sciences, Vanderbilt University, Nashville, Tennessee, United States of America; 11 Vanderbilt Evolutionary Studies Initiative, Vanderbilt University, Nashville, Tennessee, United States of America; University of Bergen, NORWAY

## Abstract

Ancient divergences within Opisthokonta—a major lineage that includes organisms in the kingdoms Animalia, Fungi, and their unicellular relatives—remain contentious. To assess progress toward a genome-scale Opisthokonta phylogeny, we conducted the most taxon rich phylogenomic analysis using sets of genes inferred with different orthology inference methods and established the geological timeline of Opisthokonta diversification. We also conducted sensitivity analysis by subsampling genes or taxa from the full data matrix based on filtering criteria previously shown to improve phylogenomic inference. We found that approximately 85% of internal branches were congruent across data matrices and the approaches used. Notably, the use of different orthology inference methods was a substantial contributor to the observed incongruence: analyses using the same set of orthologs showed high congruence of 97% to 98%, whereas different sets of orthologs resulted in somewhat lower congruence (87% to 91%). Examination of unicellular Holozoa relationships suggests that the instability observed across varying gene sets may stem from weak phylogenetic signals. Our results provide a comprehensive Opisthokonta phylogenomic framework that will be useful for illuminating ancient evolutionary episodes concerning the origin and diversification of the 2 major eukaryotic kingdoms and emphasize the importance of investigating effects of orthology inference on phylogenetic analyses to resolve ancient divergences.

## Abstract 2

Opisthokonta is the major lineage that includes animals, fungi, and their unicellular relatives, but some ancient divergences remain contentious. This study presents a genome-scale phylogeny and establishes the geological timeline of opisthokont diversification.

## Introduction

**Fig 1:**
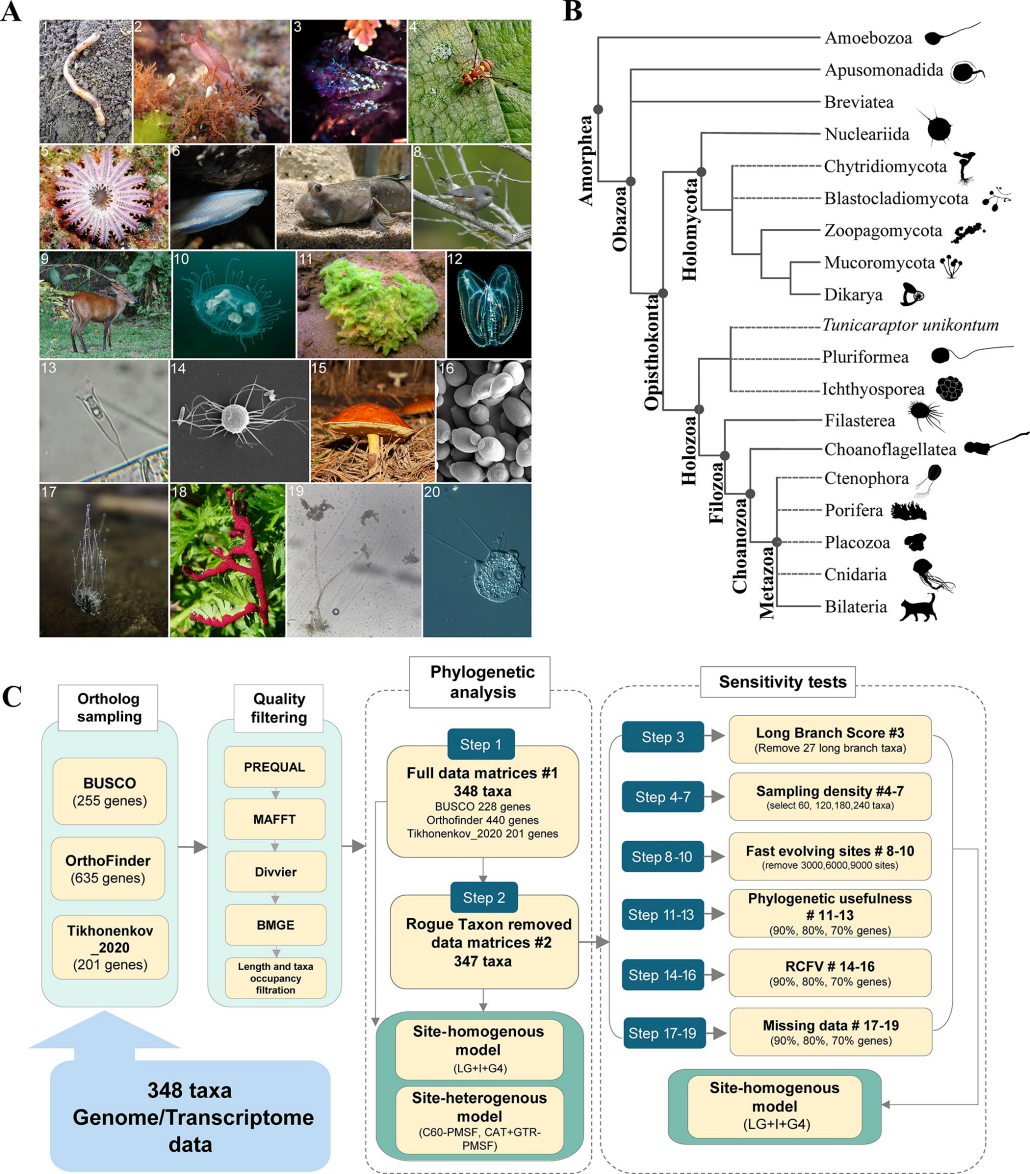
Diversity of major Opisthokonta lineages and incongruence across the Opisthokonta tree of life, and a workflow for examining evolutionary relationships. (**A**) (1) Common earthworm *Lumbricus terrestris* (Annelida); (2) California sea hare *Aplysia californica* (Mollusca); (3) common bugula, Bugula neritina (Bryozoa); (4) melon fly *Zeugodacus cucurbitae* (Arthopoda); (5) crown-of-thorns starfish Acanthaster planci (Echinodermata); (6) lancelets *Epigonichthys hectori* (Cephalochordata); (7) great blue spotted mudskipper *Boleophthalmus pectinirostris* (Actinopterygii and Chordata); (8) réunion gray white-eye *Zosterops borbonicus* (Aves and Chordata); (9) Southern red muntjac *Muntiacus muntjak* (Mammalia and Chordata); (10) peach blossom jellyfish *Craspedacusta sowerbii* (Cnidaria); (11) *Spongilla lacustris* (Porifera); (12) warty comb jelly *Mnemiopsis leidyi* (Ctenophora); (13) *Salpingoeca gracilis* (Choanoflagellatea); (14) *Ministeria vibrans* (Filasterea); (15) *Suillus luteus* (Basidiomycota); (16) Baker’s yeast *Saccharomyces cerevisiae* (Ascomycota); (17) *Phycomyces blakesleeanus* (Mucoromycota); (18) *Synchytrium papillatum* (Chytridiomycota); (19) *Rhopalomyces elegans* (Zoopagomycota); (20) *Nuclearia thermophila* (Nucleariida). Images 7, 14, 16, and 20 are available in the public domain and were sourced from Wikimedia Commons (https://commons.wikimedia.org/wiki/Main_Page). The rest of the images were retrieved from iNaturalist (https://www.inaturalist.org/). All images are credited to various artists under Creative Commons licenses with slight modifications. For specific author names, hyperlinks to the images, and copyright license details, please refer to S1 Table. (**B**) Schematic representation of the phylogenetic relationships of Opisthokonta based on recent molecular phylogenies [11,19,20]. Dashed branches reflect uncertain relationships across Opisthokonta. (**C**) A workflow that broadly samples gene and model space and implements sensitivity analyses to dissect sources of error. Data matrices are referenced throughout the text as BUSCO, OrthoFinder, and Tikhonenkov_2020. Subsampled data matrices have numbers following the “#” character reflecting the filtering step used to generate them. Each step of the sensitivity test was conducted independently. Detailed information on each data matrix is provided in S2 Table, and explanations for each subsampling strategy are outlined in the Methods section. BUSCO, Benchmarking Universal Single-Copy Orthologs.

Opisthokonta, a monophyletic supergroup containing animals, fungi, and their unicellular relatives (Fig 1A) [1–3], is divided into 2 main lineages: Holomycota [4], containing fungi and their unicellular relatives (e.g., Nucleariida), and Holozoa [5,6], which includes Metazoa (Porifera, Placozoa, Ctenophora, Cnidaria, and Bilateria) and their unicellular relatives (e.g., Choanoflagellata [7], Filasterea [8], Ichthyosporea [9,10], and Pluriformea/Corallochytrea (hereafter referred to as Pluriformea) [11]) (Fig 1B). Establishing evolutionary relationships among major lineages of Opisthokonta is key for illuminating the origins of animals and fungi, as well as of complex phenotypes like multicellularity [11–18].

In retrospect, research into the evolutionary relationships within the Opisthokonta supergroup has often focused on in-depth analyses of specific clades or lineages (e.g., [21–25]). These studies have frequently yielded conflicting hypotheses or provided equivocal support for phylogenetic relationships among some higher taxonomic ranks within Opisthokonta. Notable examples of such ambiguity within Holozoa include the relationships of unicellular holozoans [11,14,18,19,26], the root position of the animal tree between Ctenophora and Porifera [23,24,27–34], and the placement of Xenacoelomorpha—potentially a sister lineage to bilaterians [35–38] or a member of Deuterostomia [39–41]. Ambiguity also exists for certain relationships within Holomycota, such as the placements of zoospore-producing fungi (Blastocladiomycota and Chytridiomycota) [19,25,34,42–45] and the parasitic fungus *Olpidium* [46,47] on the fungal phylogeny.

Phylogenomic approaches that use genome-scale data have become the gold standard for understanding the evolution of the Opisthokonta tree of life [25,48–52]. Opisthokonta represents a remarkably diverse supergroup, but so far phylogenomic analyses of the entire supergroup have frequently been hampered by sparse taxon sampling and incomplete lineage representation (e.g., previous data matrices contained 78 genes from 58 taxa [14], 93 genes from 83 taxa [19], 255 genes from 38 taxa [11], and 201 genes from 75 taxa [18]). These data matrices captured a very small part of the full genetic diversity of the supergroup, suggesting that more in-depth data matrices and investigations of phylogenetic relationships are necessary. Furthermore, phylogenomic investigations of ancient divergences are prone to systematic and analytical errors that give rise to incongruence [53,54]. One type of error that is often overlooked is the effect of gene selection on phylogenomic inference. Variability in gene selection between studies stems from the diverse methodologies employed in identifying and choosing genes for inclusion in phylogenetic matrices. It has been shown that different gene sets, dictated by varying orthology inference methods, can markedly alter phylogenetic reconstructions [55]. Despite this, studies considering the impact of orthology inference on species tree reconstruction are scarce [56,57].

Typically, a “well-established” phylogeny should be robustly supported by independent data sources, experimental designs, and methodologies [30]. In this study, we leverage extensive genomic data from 348 taxa spanning 33 major lineages (recognized at phylum level, S3 Table) to reconstruct a comprehensive genome-scale phylogeny of the supergroup Opisthokonta and its timescale of diversification. We build 3 data matrices to assess the impact of different orthology inference methods on the resulting topologies. Through the exploration of multiple phylogenetic reconstruction parameters, we test for susceptibility to systematic errors and evaluate the robustness of our phylogenetic conclusions. The results of this study represent a nuanced understanding of the complexities in resolving the evolutionary relationships within Opisthokonta and bring the importance of orthology inference benchmarking into focus.

## Results and discussion

### Phylogenomics uncovers a broadly supported Opisthokonta tree of life

**Fig 2:**
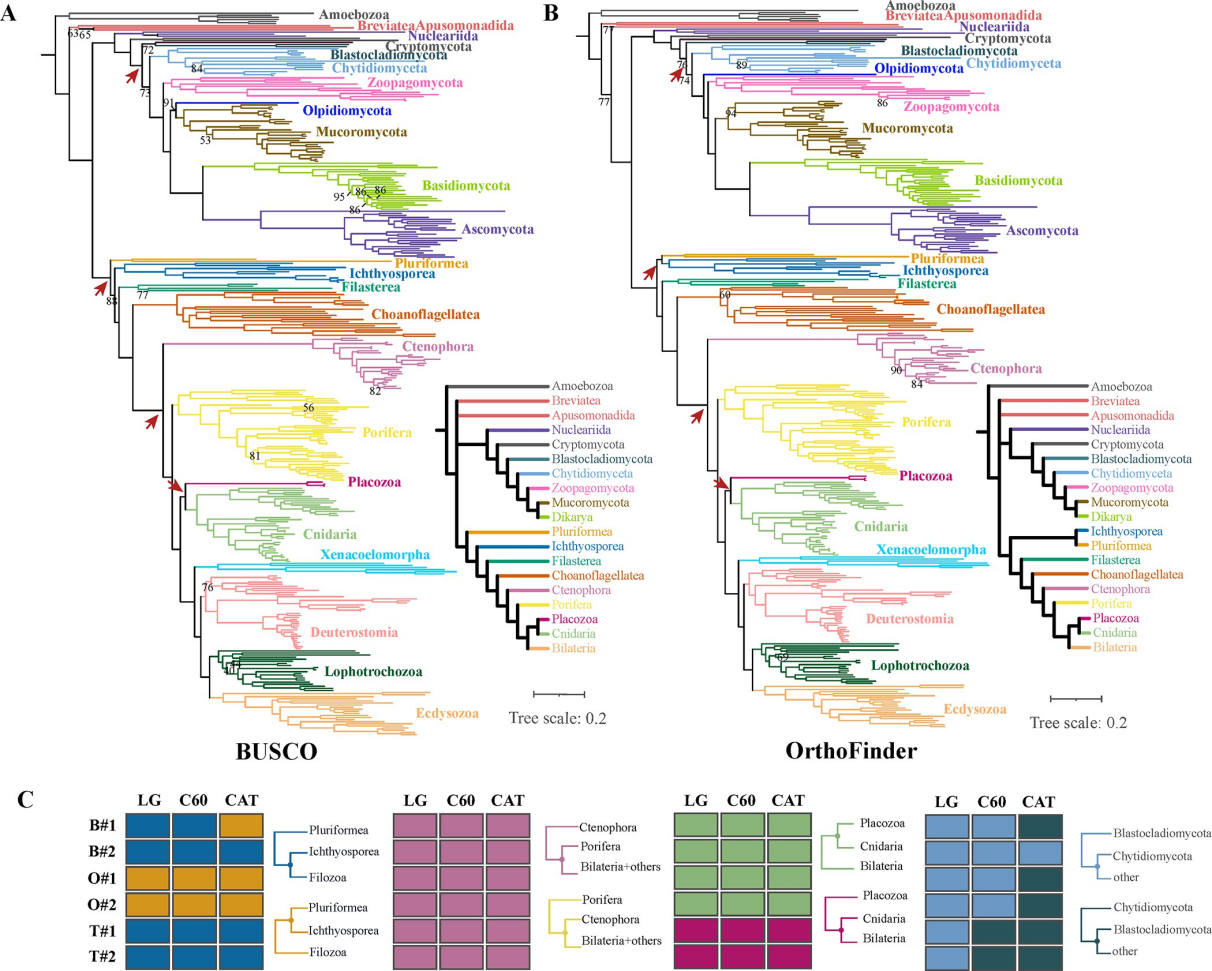
Comparison of trees obtained using IQ-TREE with the LG+C60 model from BUSCO and OrthoFinder data matrices. (**A**) The topology of the IQ-TREE 2 inference with the BUSCO data matrix#2 using the LG+C60 model. (**B**) The topology of the IQ-TREE 2 inference with the OrthoFinder data matrix#2 using the LG+C60 model. The resulting topologies of the C60 model are treated as the preferred topologies because they show the least gene tree and species tree discordance evaluated using Robinson–Foulds distance [66]. Unlabeled nodes received UFB support above 95. The cladograms are phylum-level depiction of phylogram relationships. (**C**) The distribution of topology supported across data matrices and evolutionary models, colored according to topology supported. The grids correspond to 4 contentious nodes labeled in panel A and B. From left to right, the first grid concerns the relationships between Pluriformea and Ichthyosporea, the second grid concerns whether Ctenophora or Porifera is the sister lineage to the rest of the Metazoa. The third grid refers to the relationships between Placozoa and Cnidaria, and the fourth grid correspond to the branching order of Blastocladiomycota and Chytridiomycota, the “B,” “O,” “T” represents BUSCO, OrthoFinder, and Tikhonenkov_2020 data matrix, respectively. The original tree files underlying this figure can be found in https://doi.org/10.6084/m9.figshare.23301824.v1. BUSCO, Benchmarking Universal Single-Copy Orthologs; UFB, ultrafast bootstrap.

To infer the Opisthokonta tree of life, 3 data matrices with high taxon sampling and gene occupancy were constructed using different orthology inference methods and rigorous quality control measures, termed BUSCO, OrthoFinder, and Tikhonenkov_2020, respectively, reflecting the origin of phylogenomic markers. The BUSCO data matrix includes 228 genes, the OrthoFinder matrix comprises 440 genes, and the Tikhonenkov_2020 matrix contains 201 genes (Fig 1C and Tables 1 and S2). The evolutionary history of Opisthokonta was inferred using both site-homogeneous and -heterogeneous models. These analyses produced 18 phylogenomic trees: 3 data matrices (BUSCO, OrthoFinder, and Tikhonenkov_2020) * 2 versions (full data matrix and rogue taxon pruned) * 3 modeling schemes (LG+I+G4, LG+PMSF(C60)+G+F, GTR+CAT+PMSF, hereafter referred to as LG, LG+C60, GTR+CAT). We found that approximately 85% of internal branches were congruent across the 18 trees, suggesting that a large fraction of bipartition in the Opisthokonta phylogeny were consistently supported (S4 Table and S1 Data). Within Holozoa, notable examples of relationships recovered uniformly in our results include Ctenophora as the sister group of the remaining Metazoa; this grouping was also stable in the subsampling analysis designed to detect potential biases (except for BUSCO#4 matrix with 60 taxa under GTR+CAT model) (Fig 2A and 2B and S1 Data). The very high consistency (80 out of 81 analyses, S5 Table) provides support for the hypothesis that ctenophores are the closest relatives of all other metazoans [23,28,33,34,58,59]. Furthermore, our results recapitulate many deep relationships recovered in previous phylogenomic studies: Bilateria, Deuterostomia, Ecdysozoa, Lophotrochozoa, Protostomia are all recovered [21,58,60–63], and we recover Xenacoelomorpha as the sister group to Bilateria (the Nephrozoa hypothesis) [35–38,64]. Our results also support the sister relationship of Filasterea to a Choanoflagellatea and Metazoa group (Filozoa hypothesis) [4,8,65], although this grouping is not always robustly supported (Fig 2A and 2B and S1 Data).

**Table 1:** Summary statistics of 3 phylogenomic data matrices.

Data matrix	Number of genes	Number of sites	Average taxon occupancy	Average gene length	Average site occupancy
BUSCO	228	72,657	79.9%	319	67.1%
OrthoFinder	440	113,123	85.6%	257	68.9%
Tikhonenkov_2020	201	95,808	87.0%	477	72.6%

Among Holomycota, examples of relationships recovered consistently in our results include the monophyly of the Dikarya subkingdom [67], comprising the Ascomycota and Basidiomycota phyla, which received maximal support across all analyses. Mucoromycota was recovered as the sister group of Dikarya [44] and Zoopagomycota is sister to both lineages [68]. Supporting a recent study, a Nucleariida clade consisting of *Parvularia atlantis*, *Fonticula alba*, and *Lithocolla globosa* was recovered as the sister lineage to the rest of the Holomycota [69] (Fig 2A and 2B and S1 Data).

### A timescale for Opisthokonta diversification

**Fig 3:**
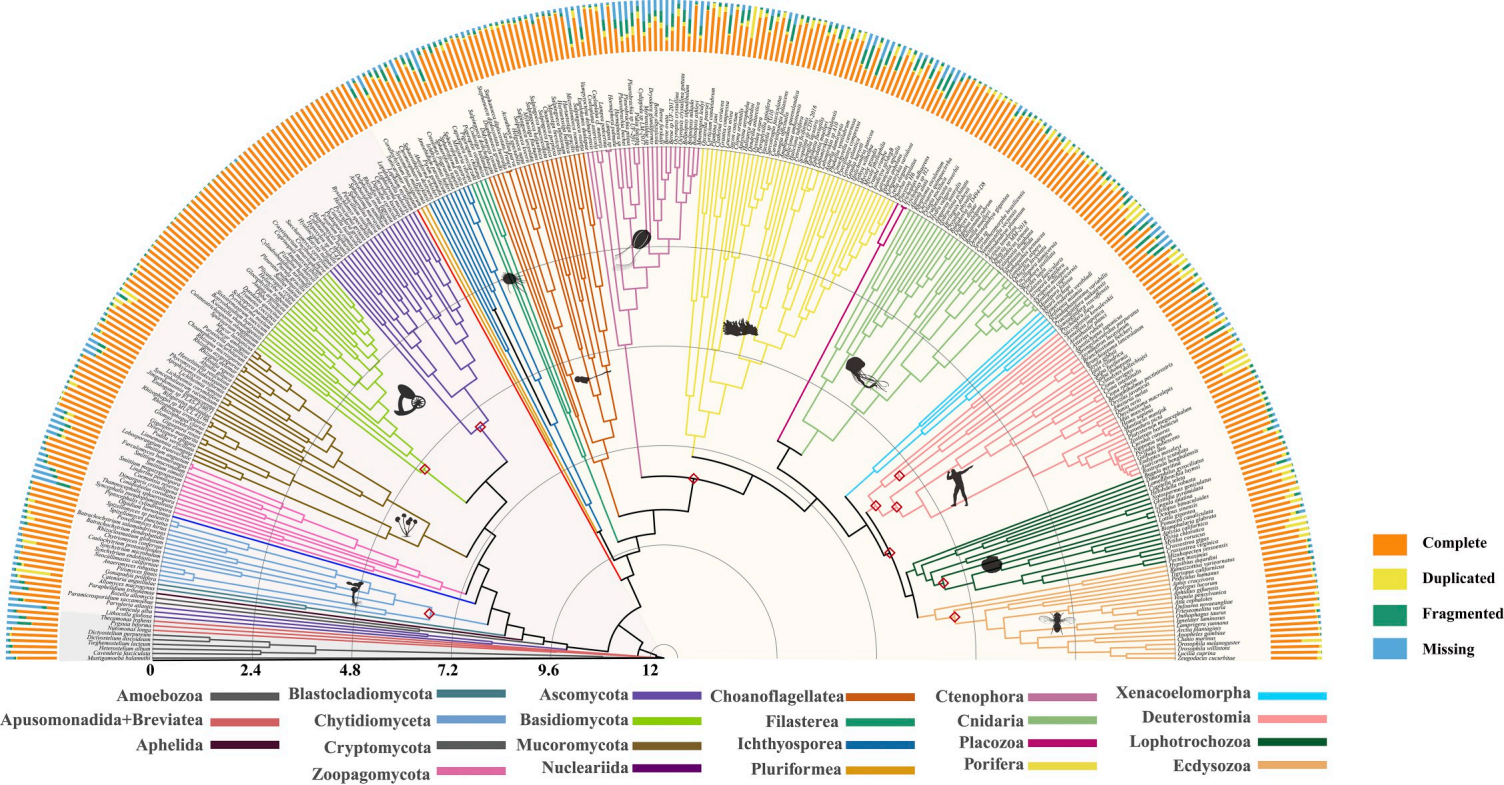
Time-calibrated phylogeny of 348 species spanning the diversity of opisthokonts. Divergence time estimation using MCMCTree with a topology reconstructed from the concatenation-based maximum likelihood analysis of OrthoFinder#1 data matrix using the LG+C60 model. The bar plot next to each species indicates genomic quality assessed using BUSCO. “Complete” indicates the fraction of full-length BUSCO genes; “Duplicated” indicated if there were 2 or more complete predicted genes for one BUSCO gene, “Fragmented” indicates the fraction of genes with a partial sequence, and “Missing” indicates the fraction of genes not found in the genome (S3 Table). Images from phylopic.org. Red diamonds represent nodes on which fossil calibration constraints were imposed. The timescale is in 100 millions of years before present. Detailed time trees could be found in S1 Data. BUSCO, Benchmarking Universal Single-Copy Orthologs.

A Bayesian relaxed molecular clock calibrated with 10 widely accepted fossil calibration points (S6 Table) facilitated estimating divergence times of Opisthokonta evolution (Figs 3 and S1 and Table 2). Estimates remain consistent across different root ages (average differences 1%, S7 Table and S1 Data), consequently, we focus our discussion on results obtained using a root age constraint of 1.5 billion years. Our analyses suggest that Opisthokonta originated approximately 1,083.2 million years ago (Mya) (95% credibility interval (CI) ranging from 978.7 to 1187.6 Mya). This result falls in the interval estimated by Eme and colleagues [70] and Parfrey and colleagues [71] across different root positions and varying molecular clock models. Holomycota is estimated to be approximately 996 Mya (95% CI: 890.1 to 1,101.9 Mya) and Holozoa emerged slightly earlier at roughly 1,003.8 Mya (95% CI; 913.8 to 1,093.9 Mya) (S1A Fig). The origin of animals, marking the emergence of animal multicellularity, began approximately 791.6 million years ago (95% CI: 745.5 to 837.8 million years ago) during the Tonian period. This timeline aligns with the widely accepted framework for animal diversification, which predicts Neoproterozoic divergences [72], and it matches the age of the oldest uncontested animal fossils [73,74] more closely compared to earlier studies that unaccounted for the rate variations of molecular evolution [75]. Our analysis also suggests origination time of Ctenophora are considerably younger than Cnidaria and Porifera, consistent with a previous study [28]. The estimated divergence time between protostomes and deuterostomes was approximately 615.9 to 651.6 Mya (mean: 633.8).

**Table 2:** Inferred 95% confidence time intervals for the various Opisthokonta clades, in millions of years before present (root age constrained to 1.5 billion years).

Crown group	min	max	width	mean
Opisthokonta	978.74	1,187.57	208.83	1,083.16
Holomycota	890.12	1,101.86	211.74	995.99
Holozoa	913.81	1,093.88	180.07	1,003.85
Choanozoa	810.4	937.27	126.87	873.84
Metazoa	745.51	837.75	92.24	791.63
Porifera-ParaHoxozoa	699.84	786.49	86.65	743.17
Bilateria	632.39	667.28	34.89	649.84
Deuterostomia	582.71	627.28	44.57	605.00
Ecdysozoa	544.71	594.87	50.16	569.79
Lophotrochozoa	574.54	608.52	33.98	591.53
Chordata	530.31	595.14	64.83	562.73
Ecdysozoa-Lophotrochozoa	596.08	632.01	35.93	614.05
Ascomycota	409.69	606.29	196.6	507.99
Basidiomycota	411.14	611.87	200.73	511.51
Mucoromycota	491.24	673.35	182.11	582.30
Zoopagomycota	583.87	758.99	175.12	671.43
Chytridiomycota	531.05	761.92	230.87	646.49
Obazoa	1,077.05	1,334.8	257.75	1,205.93

Within Holomycota, the origin of the kingdom Fungi—sister clade to Nucleariida—was dated to approximately 929.2 Mya (95% CI, 825.2 to 1,033.3 Mya). This estimate is consistent with the oldest putative fossil of fungi, dated approximately between 1,010 and 890 Mya [76]. However, it is important to note that the earliest unambiguously accepted fungal fossil, verified through microscopic and spectroscopic techniques, dates to 810 to 715 Mya [77]. The origin of terrestrial fungi was estimated at 731.7 Mya (95% CI: 645.1 to 818.2 Mya), in line with a previous report [43]. The origin of Dikarya was estimated to be around 623.9 Mya (95% CI: 539.3 to 708.4 Mya).

To compare the rate of diversification across major lineages of Opisthokonta, we utilized a lineage-through-time (LTT) plot [78,79] to examine the temporal patterns of diversification within 12 defined subgroups (S1 Fig caption). This analysis involved plotting the logarithm of the number of taxa in each subgroup across various time slices (S1B Fig). Notably, the time span from late Neoproterozoic to early Cambrian marked a period of pronounced diversification among major animal groups, such as Lophotrochozoa and Deuterostomia (S1B Fig), likely reflecting the Cambrian radiation of animals [80]. However, the LTT plots for fungal subgroups do not adequately capture the documented drastic increase in diversification rates within the kingdom fungi, such as the radiation of Leotiomyceta beginning around 450 million years ago [81].

By increasing our taxon sampling and employing advanced analytical techniques, this study infers the first detailed timetree of Opisthokonta. These results may inform the testing of hypotheses that tie the emergence of lineages and phenotypes to specific geologic events. For example, molecular dating analyses have consistently placed the emergence of animals in the Tonian-Cryogenian period, approximately 850 to 635 Mya [73,80,82], broadly coinciding with the rise in atmospheric oxygen levels and changes in the phosphorus cycle [83,84]. The detailed temporal diversification patterns revealed among key Opisthokonta subgroups provide valuable insights into the evolutionary trajectories that have shaped current biodiversity, enhancing our understanding of how geological and environmental factors have influenced diversification of Opisthokonta.

### Incongruences in the Opisthokonta phylogeny

Approximately 15% of bipartitions in the Opisthokonta phylogeny, some affecting higher opisthokont taxonomic ranks, were unstable across data matrices and approaches used. Below, we discuss key incongruent relationships of interest. For each case of instability, we detail the outcomes from different data matrices and analytical methods and highlight where these differences significantly impact the results (S5 Table and S1 Data).

### Uncovering novel relationships among unicellular holozoans

One notable example of incongruence concerned the relationships among unicellular ancestors of animals. Resolving ancient branching patterns among unicellular Holozoa have proven recalcitrant, wherein different phylogenomic studies support conflicting topologies or are equivocal in support [11,14,18–20]. Our analyses using the BUSCO and Tikhonenkov_2020 data matrices recovered a novel resolution where Pluriformea is the sister group to the remaining holozoans (Pluriformea-sister hypothesis, Fig 2A and S1 Data). In contrast, the OrthoFinder data matrix suggests that Pluriformea is the sister taxon to Ichthyosporea (known as the Teretosporea group), as reported in previous studies [19,20,26] (Teretosporea-sister hypotheses, Fig 2B and S1 Data). Relationships among unicellular Holozoa are robust to substitution model complexity, except for one instance in which the BUSCO#1 matrix with GTR+CAT model weakly supported Teretosporea-sister (UFB = 23, S1 Data). Surprisingly, the third alternative topology, which supports Ichthyosporea as the sister taxon to all other Holozoa (Ichthyosporea-sister hypothesis) [11,18] was not recovered in our analyses.

Recent studies have uncovered that the unicellular ancestors of animals have a suite of genetic elements traditionally associated with animal multicellularity (such as cell adhesion, signaling, and transcriptional regulation) [2,11,20,26]. Consequently, the branching order of unicellular relatives of animals is essential for interpreting the sequence of events that led to the emergence of animals and their potential contributions to the origin of multicellularity. For example, the Ichthyosporea-sister hypothesis suggests that an animal-like extracellular matrix (ECM) structure arose in a common ancestor shared by Pluriformea, Filasterea, Choanoflagellata, and Metazoa, subsequent to their evolutionary split from the Ichthyosporea [11]. Interestingly, despite utilizing the same gene set as Tikhonenkov and colleagues [18], our analysis yielded a different topology (Pluriformea-sister hypothesis versus Ichthyosporea-sister hypothesis), marking this as a particularly intriguing case that warrants further investigation, as discussed below.

### Revisiting the placement of Placozoa

The position of Placozoa also showed conflict: the Tikhonenkov_2020 matrix supports the sister relationship between Cnidaria and Bilateria with Placozoa as sister to this clade (Fig 2C and S1 Data). In contrast, the BUSCO and OrthoFinder matrices recovered a sister taxon relationship between Placozoa and Cnidaria (Fig 2C and S1 Data). This discrepancy was reported before and has been attributed to the effect of compositional heterogeneity [85,86]. Specifically, Laumer and colleagues [85] generated 2 ortholog sets, with one indicating a sister relationship between Placozoa and Cnidaria (derived from OrthoFinder orthologs), and the other positioning Placozoa as a sister lineage to both Cnidaria and Bilateria (using BUSCO genes). Through a null-simulation test for compositional bias, they suggested that the latter topology might be an artifact of compositional heterogeneity. In a subsequent study, Laumer and colleagues [86] reinforced the support for the Placozoa + Cnidaria clade by employing a data matrix that reduces compositional heterogeneity through Dayhoff recoding.

Notably, our subsampling analysis demonstrates the potential impact of compositional heterogeneity, as well as missing data on the phylogenetic topology derived from the Tikhonenkov_2020 data matrix: excluding genes with high compositional heterogeneity (measured by RCFV scores, see Methods section) alters the resulting topologies but favors neither 2 hypotheses (S5 Table and S1 Data); excluding genes with high amount of missing data shifts the support towards the sister relationship between Placozoa and Cnidaria. However, the influence of gene subsampling based on different criteria appears to be matrix specific and not universally effective across different data sets.

### The relationships between Chytridiomycota and Blastocladiomycota

The relationships between flagellated zoosporic fungi Blastocladiomycota and Chytridiomycota have been contentious [25,42–45]. Understanding the phylogenetic placement of Blastocladiomycota, which display many terrestrial fungal characteristics including developed hyphae, spore-bearing structures for the dissemination of sexual and asexual spores, closed mitosis, β-1-3-glucan cell walls, and a Spitzenkörper [87,88], is crucial for elucidating the evolution of structural complexity, reproductive strategies, and adaptive mechanisms that have shaped fungal diversity. In our analysis, we observed that Blastocladiomycota as sister to Chytridiomycota and other fungi is consistently recovered using the site-homogeneous LG+I+G4 model. Conversely, the designation of Chytridiomycota as the sister group to the rest of the fungi could only be recovered under site-heterogeneous models, though this is not observed across all data matrices (Fig 2C and S5 Table and S1 Data). For example, analyses using the BUSCO and OrthoFinder data matrices with the C60 model still recover the same topology as produced by the site-homogeneous model (Fig 2B and 2C and S5 Table and S1 Data). Notably, recent studies using site-heterogeneous models (e.g., C models and CAT) support the divergence of Blastocladiomycota following that of Chytridiomycota [45,89].

In addition, the placement of the endoparasitic zoosporic fungus *Olpidium* was unstable and data matrix dependent. OrthoFinder and Tikhonenkov_2020 data matrices strongly supported *Olpidium* as sister to a clade of non-flagellated terrestrial fungi (Fig 2B and S1 Data), in line with the most parsimonious explanation for the loss of the fungal flagellum [47,89,90]. However, the BUSCO data matrices supported *Olpidium* nested within non-flagellated fungi, either as the sister group of Mucoromycota, or as the sister group to Dikarya (Fig 2A and S1 Data).

### Different orthology inference methods contribute to incongruence

**Fig 4:**
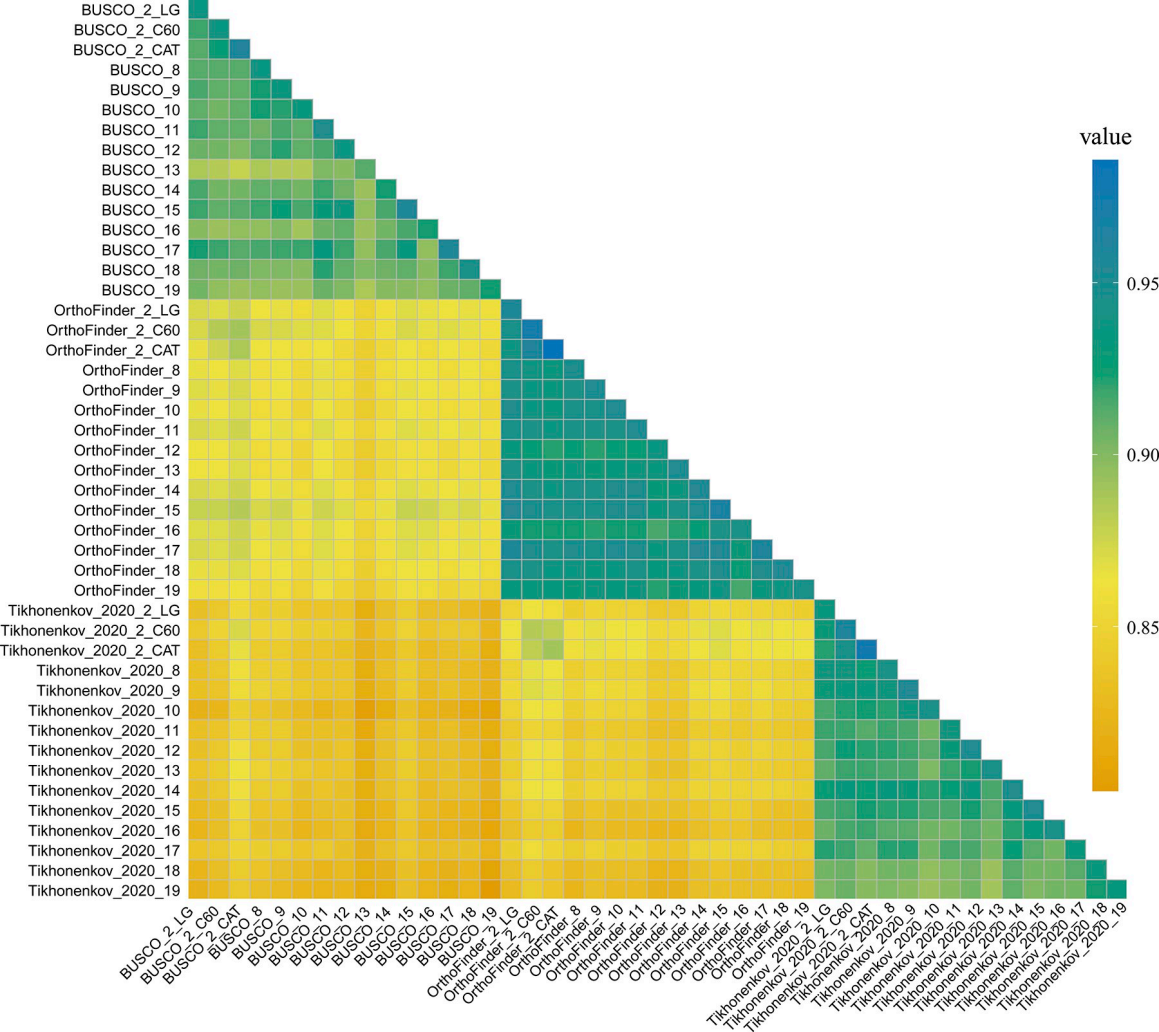
Heatmap of topological similarities for all pairwise comparisons among the phylogenies reconstructed from 39 data matrices (#2, #8–19). The topological congruence between each pair of phylogenies was calculated using GoTree [91], function “compare.” To ensure that only highly supported relationships are illustrated, nodes with UFB support less than 95 were collapsed prior to comparison. The color of the squares represents the percentage of bipartitions (n/344) shared between trees. Results from data matrices#3–7 are not compared here since they do not share the same number of tree tips. A dendrogram constructed from a Euclidean distance matrix—calculated based on the number of shared bipartitions across data sets—is provided in S3 Fig. Full illustration of the resulting topologies could be found in S1 Data. The code used to generate this plot is available in https://doi.org/10.6084/m9.figshare.23301824.v1. UFB, ultrafast bootstrap.

Phylogenetic analysis using different models and sensitivity analysis—reinferring species-level relationships using 18 subsampling strategies—revealed high degrees of congruence in analyses of the same data matrix, but not in analyses of different data matrices. Specifically, phylogenies inferred using BUSCO, OrthoFinder, and Tikhonenkov_2020 data matrices and their subsets shared 97.5%, 98.2%, and 97.3% of bipartitions, respectively, whereas the average bipartitions shared among different data matrices were 87.7% (BUSCO versus Tikhonenkov_2020), 88.8% (OrthoFinder versus Tikhonenkov_2020), and 90.8% (BUSCO versus OrthoFinder) (Fig 4 and S4 Table).

The very high congruence within the same ortholog set and the varying sensitivity to approaches used (modeling schemes and subsampling analysis) suggest gene sets derived using different orthology methods might be a source of incongruence for the Opisthokonta phylogeny. To explore this possibility further, we first analyzed the gene overlap among the 3 data matrices. The results revealed significant disparities: about 44% (100 out of 228) of the BUSCO genes were recovered by OrthoFinder data matrix, while BUSCO and OrthoFinder contain only about 22% (44 out of 201) and 30% (61 out of 201) of the genes present in the Tikhonenkov_2020 data matrix, respectively (S8 Table). Approximately half of the genes in each data matrix are absent in the other two, with only 19 genes present across all 3 data matrices (S2A Fig and S9 Table). Additionally, there is variation in the functional categories represented in each matrix. For example, the Translation (J) category is the most abundantly represented in both the BUSCO (15.2%) and Tikhonenkov_2020 (22.1%) matrices while the OrthoFinder matrix is dominated by the secondary metabolism (O) category (14.5%) (S2B Fig).

Due to functional constraints and different evolutionary trajectories, genes may contain positions that vary in their functional constraint, resulting in varying saturation levels among data sets [53]. To test this hypothesis, we quantified the saturation level of the data matrices following Philippe and colleagues (48) using PhyKIT [92]; data with no saturation will have a value of 1, while a value of 0 means complete data saturation. We found that the Tikhonenkov_2020 data matrices were the most saturated (approximately 0.12) and that the OrthoFinder data matrices were the least affected by multiple substitutions (approximately 0.24). The varying degrees of saturation may contribute to the observed incongruence among the 3 data matrices (S10 Table).

In assessing the relative quality of different ortholog sets, we focused on their “information content” through a sensitivity analysis of submatrices derived from 3 data matrices. We evaluated several metrics using PhyKIT including average bootstrap score, saturation, Robinson–Foulds distance, and treeness/RCV—a measure indicates signal-to-noise ratio and susceptibility to composition bias. Statistical analysis using ANOVA demonstrated no significant differences in average bootstrap support (*p*-value = 0.94) and Robinson–Foulds distance (*p*-value = 0.52) among the data matrices. However, submatrices derived from OrthoFinder exhibited significantly lower saturation levels (*p*-value = 1.91e-14) and higher treeness/RCV values (*p*-value = 7.87e-32), indicating a potentially superior information content. These results suggest that the OrthoFinder data matrix may provide enhanced robustness for phylogenetic analyses.

Our results suggest that variation in ortholog selection between data matrices is a significant contributor to incongruence. Notably, recent investigations have documented significant variances in both the orthologs identified and the resulting phylogenetic trees when employing diverse orthologous group reconstruction methodologies [49,55,93]. Despite the availability of various automated orthology inference methods, achieving standardized ortholog benchmarking remains a challenge. This issue affects not only phylogenetic analysis but also extends to broader aspects of evolutionary biology, such as comparative genomic analysis, the identification of chromosome fusions, and more. Evaluating multiple orthology inference methods and comparing how they affect species tree reconstruction should be considered a good practice in refining phylogenetic histories.

### The intricacies of unicellular holozoan relationships

**Fig 5:**
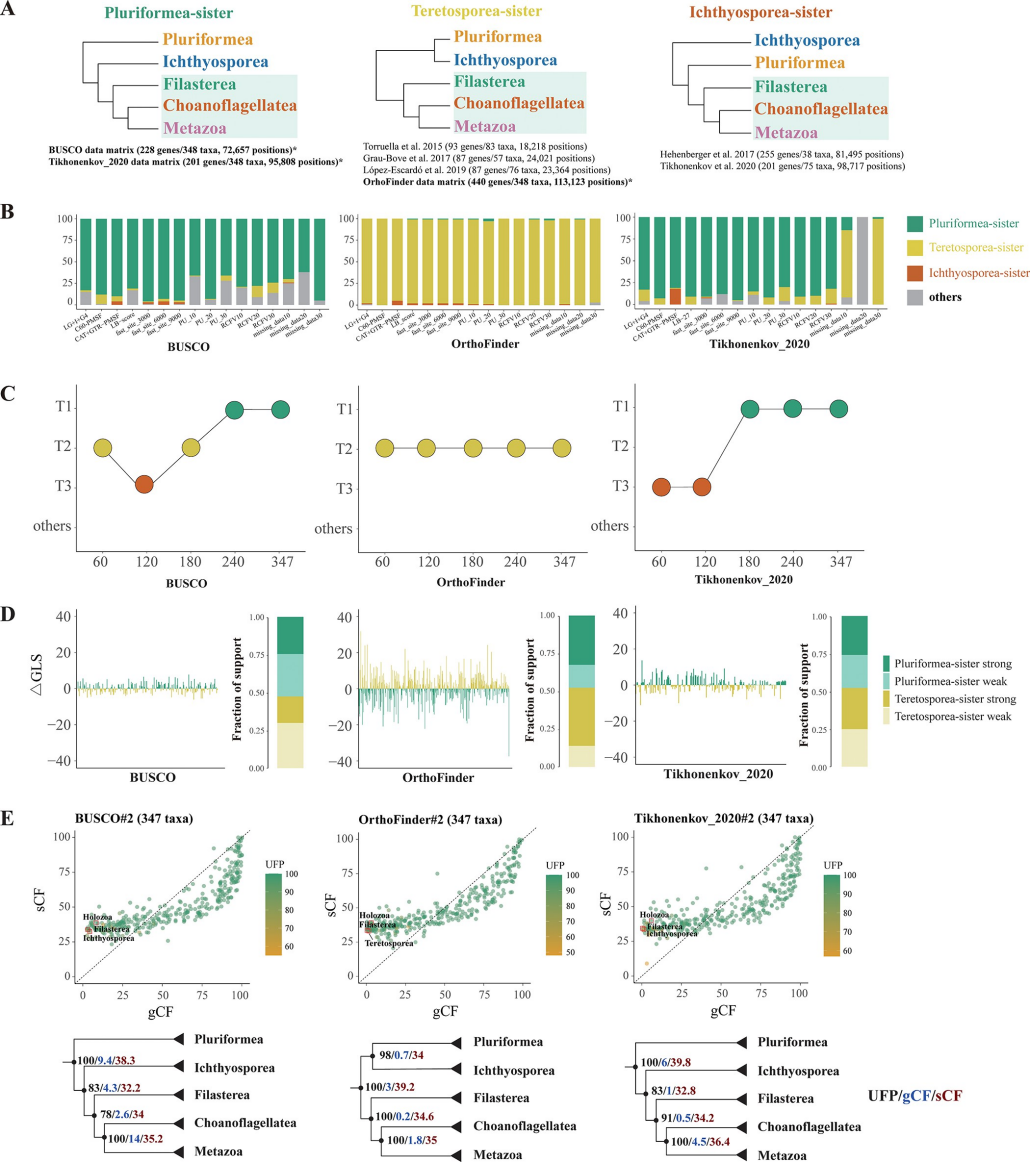
Integrated analysis of alternative phylogenetic hypotheses. (**A**) Alternative hypotheses of the relationships of unicellular holozoans. Studies that support these hypotheses are listed below each tree; studies with an asterisk are results from this study. The 3 hypotheses from left to right are Pluriformea is the sister lineage to the rest of the Holozoa, a clade of Pluriformea + Ichthyosporea as the sister lineage to the rest of the Holozoa, and Ichthyosporea as the sister lineage to the rest of the Holozoa, respectively. (**B**) Bootstrap support values for alternative hypotheses across different data sets are presented. The stack bar plots indicate the occurrence frequencies of each topology in 1,000 UFB trees. (**C**) Topological differences among different taxon-sampling densities and modeling schemes. Initially, we selected 60 taxa to cover the diversity of Opisthokonta; subsequent increments in taxon sampling were done by randomly selecting additional sets of 60 taxa at each step. (**D**) Bar plot of the difference in gene log-likelihood scores (ΔGLS) between the 2 hypotheses recovered in this study. Proportions of genes supporting each of 2 alternative hypotheses for 3 data matrices are also shown. The ΔGLS values for the genes across each data matrix can be found in the S12 Table. We considered a gene with an absolute value of log-likelihood difference of 2 as a gene with strong (|ΔGLS| > 2) or weak (|ΔGLS| < 2) phylogenetic signal. (**E**) The distribution of gCFs and sCFs across all nodes of the Opisthokonta tree. Critical nodes concerning the relationships of unicellular Holozoa were labeled. The actual values of gCF, sCF, and UFB for the nodes concerning the relationships of unicellular holozoans were labeled on the schematic tree. The data and code underlying this figure is available at https://doi.org/10.6084/m9.figshare.23301824.v1. The script for panel E can be found in http://www.robertlanfear.com/blog/files/archive-2018.html. gCF, gene concordance factor; sCF, site concordance factor; UFB, ultrafast bootstrap.

Relationships within unicellular holozoans were a particularly interesting example of the effect of different orthology inference methods on phylogenetic reconstruction. Observing differing results despite utilizing the same gene set as a previous study prompted us to undertake a comprehensive investigation to explore these discrepancies. Specifically, despite using the same set of genes and evolutionary models with similar complexity (CAT+GTR+PMSF here versus CAT+GTR in the original study) [18], the Tikhonenkov_2020 matrix here recovered Pluriformea-sister hypothesis, a topology that has not been recovered previously. In contrast, the original analyses by Tikhonenkov and colleagues [18] provided support for the Ichthyosporea-sister hypothesis (Fig 5A). This topology was not recovered in our analysis and was rarely observed among UFB approximated trees, indicating that it received minimal support (Fig 5B and S11 Table).

In addition, sensitivity analysis revealed no significant predictors of topological preference. Although the removal of 20% of the missing data led to topological changes in unicellular holozoans, this resulted in a topology that is likely to be erroneous [14]. Moreover, the effects of data removal were not consistent (Fig 5B), the possibility of this result simply being due to a decrease in the number of positions analyzed cannot be excluded. These findings imply that factors beyond the orthology inference methods and systematic errors tested may be influencing the results.

A key difference between this study and Tikhonenkov and colleagues [18] is the number of taxa sampled, raising the hypothesis that increased taxon sampling density could affect the relationships of unicellular holozoans. To test this hypothesis, we created submatrices by down-sampling data sets to a number of taxa comparable to previous studies [11,18–20] (N_taxa_ = 60; data matrices #4) and conducted phylogenetic inference using the CAT-GTR model under the PMSF assumption (S2 Table). As anticipated, the topology of Tikhonenkov_2020#4 (60 taxa) shifted to support the Ichthyosporea-sister hypothesis (Fig 5C), aligning with the results of [18]. In contrast, expanding the sampling density to 180, 240, and 347 taxa led to robust support for the Pluriformea-sister hypothesis (Fig 5C and S1 Data). Notably, Ichthyosporea-sister topology was also recovered when down-sampling BUSCO data matrix to 120 taxa (Fig 5C and S1 Data). To examine the potential influence of outgroup sampling on this part of the tree, we excluded remote outgroups and restricted our analysis to taxa from Holozoa and Holomycota with 3 rogue removed data matrices, both BUSCO and Tikhonenkov_2020 data matrices inferred identical unicellular holozoa relationships (Pluriformea-sister) as in analyses performed with full outgroup sampling, suggests that the Pluriformea-sister hypothesis is likely not an artifact driven by the inclusion of distant outgroups. These analyses suggest taxon sampling density plays a significant role in shaping the phylogenetic landscape of unicellular holozoans. The impact on the resulting topology, however, depends on the specific matrix employed.

To further explore incongruence in relationships of unicellular holozoans across 3 data matrices, we employed gene-wise likelihood scores (ΔGLS values) and concordance factors to quantify the phylogenetic signal for 2 contrasting topologies (Pluriformea-sister and Teretosporea-sister) across 3 data matrices. The results of ΔGLS values indicate varying strengths of phylogenetic signals across data matrices. Specifically, the OrthoFinder#2 data matrix had stronger phylogenetic signals than the other 2 (average |ΔGLS| = 5.33, compared to 2.68 Tikhonenkov_2020#2 matrix and 1.91 in BUSCO#2 matrix). Despite these differences, the proportions of genes supporting 2 hypotheses were close to a 50–50 ratio across all matrices (Fig 5D and S12 Table), suggestive of ambiguous phylogenetic signals regarding this part of the tree. Furthermore, the distribution of gene- and site-concordance factors (gCF and sCF, respectively)—measures for quantifying genealogical concordance in phylogenomic data sets, showed low gene tree concordance, contentious nodes with high UFB support constantly had low gCF scores (Fig 5E and S13 Table). For example, despite the Teretosporea-sister hypothesis being strongly supported using the OrthoFinder#2 matrix under a site-homogeneous model (UFB support = 98), gCFs revealed that only 0.7% (3/426) of individual loci supported the Teretosporea-sister hypothesis, and up to 98.6% (420/426) of gene trees supported topologies other than the 3 candidate topologies. Examining sCF values revealed substantial noise among single sites evidenced by a similar proportion of support for each hypothesis (34.04/32.98/32.98; S13 Table).

Robust phylogenetic relationships across various orthology methods may reflect strong phylogenetic signals in the data [93]. Examination of the distribution of support from individual genes reveal weak signals in single loci and their respective sites regarding the relationships of unicellular holozoans, might be the underlying reason for the lack of robustness to different orthology inference methods. In cases when signals are weak, comparing the performance of different orthology methods becomes particularly crucial. The observed scarcity of phylogenetic signals in our study underscores the need for further research to confidently resolve the relationships among unicellular holozoans. Future investigations will benefit from the precise identification of orthologs and the inclusion of additional genomic data from unicellular Holozoa to clarify the currently uncertain relationships.

## Conclusion

In this study, we curated three phylogenomic matrices with high taxon sampling and occupancy; we analyzed these matrices using a phylogenomic workflow (Fig 1C) that we devised to examine artifacts and evaluate the robustness of phylogenomic inference. Using this workflow, we inferred a genome-scale and taxon-rich phylogeny of Opisthokonta with a timescale of diversification from the Mesoproterozoic era to the present and identified contentious branches warranting further investigation (Figs 2 and 3). Our analyses reveal that varying gene sets from different orthology methods contribute to incongruence in the Opisthokonta tree of life. Together with previous reports [11,18–20], 3 topologies have received support concerning the root of the Holozoa tree (Fig 5A), our analysis underscores the crucial role of taxon sampling density in shaping these relationships (Fig 5C). However, the weak phylogenetic signals observed suggest that resolving this part of the tree remains one of the most challenging enigmas in the phylogenomic era (Fig 5D and 5E). Additional genomic data from unicellular holozoans may be key to achieving further resolution. Our study assesses the current state of progress toward a fully resolved Opisthokonta tree of life; the methodologies developed herein could be adapted for detailed investigations into other lineages within the tree of life.

## Methods

### Data acquisition

Genome and transcriptome data for over 800 Opisthokonta species were retrieved from public databases. Transcriptome data were included due to the limited availability of genomic data for certain lineages, such as unicellular holozoans, Ctenophora, Porifera, and Cnidaria. Representatives of fast-evolving lineages containing pathogens and parasites known to cause long-branch attraction (LBA) were excluded (i.e., Microsporidia, Platyhelminthes, Nematoda) [60,94]. To minimize the amount of missing data and remove potential low-quality genomes/transcriptomes, completeness was assessed using the Benchmarking Universal Single-Copy Orthologs (BUSCO) v5.02 [95] pipeline with the eukaryotic_odb10 database (255 near-universally single-copy orthologs or BUSCO genes; last accession date: June 14, 2022) [96]. BUSCO genes were classified as single-copy, duplicated, fragmented, or missing based on the presence/absence, copy number, and length of the predicted BUSCO gene; the fraction of single-copy BUSCO genes present is a proxy for assembly completeness. With the exception of unicellular lineages and non-bilaterian animal lineages, other taxa were filtered based on BUSCO gene completeness while also ensuring a balanced representation of different Opisthokonta lineages. The final list contained 339 Opisthokonta species (217 genomes and 122 transcriptomes). Additionally, 9 outgroup taxa were downloaded from NCBI (last accession date: December 17, 2022) based on the current understanding of Opisthokonta phylogeny [14,19] (S3 Table). Our study presents the most comprehensive collection of unicellular holozoans to date, incorporating genome data from 4 Filasterea species [17]. We have also included genomic and transcriptomic data from an extensive set of 10 Ichthyosporea species, along with data from 2 Pluriformea taxa: *Corallochytrium* and *Syssomonas*.

### Construction of 3 phylogenomic data matrices

Orthology inference plays a crucial role in the phylogenomic analyses. Despite the burgeoning of available methods, their impact on downstream phylogenetic analysis was rarely compared, and few studies have regarded orthology methods as an influencing factor in phylogenetic reconstructions [56,57]. To explore the performance of different ortholog inference methods in the context of Opisthokonta tree of life, we constructed 2 novel data matrices using different strategies—that is, targeted identification of phylogenomic markers (BUSCO) and de novo inference (OrthoFinder), both are popular and widely utilized in phylogenomic studies [18,25,93,95,97–99]. Additionally, we utilized a data set based on a set of genes from an earlier phylogenomic study [18] to facilitate direct comparisons with prior findings; this approach also provides a unique opportunity to assess the impact of taxon sampling density (Fig 2).

#### (i) BUSCO data matrix

BUSCO aims to identify putatively orthologous genes using a predetermined set of profile hidden Markov model sequence alignments (pHMMs) derived from single-copy orthologous proteins from the OrthoDB database [95,100]. BUSCO genes have been used as phylogenomic markers in diverse lineages [25,95,101]. Therefore, a data matrix was constructed using complete, single-copy sequences identified with the BUSCO algorithm as described above, resulting in 255 single-copy orthologs.

#### (ii) OrthoFinder data matrix

The OrthoFinder software conducts BLAST all-vs-all searches across proteomes to infer groups of putatively orthologous genes [102]. Orthologous groups were initially constructed using the genomic data from 52 taxa—49 Opisthokonta species and 3 outgroup taxa (2 amoebozoans and 1 apusomonadid). Each major Opisthokonta lineage was represented by 1 to 3 taxa with the best assembly quality (S14 Table). OrthoFinder v2.5.4 [102] was used to identify putatively orthologous sequences shared among taxa using default parameters (inflation parameter 1.5). To identify additional phylogenomic makers, species-specific inparalogs—genes that have undergone duplication events along terminal taxa—were pruned from groups of orthologous genes [103,104]. To do so, orthogroups with greater than or equal to 80% taxon occupancy (*N* = 42) were aligned with MAFFT v7.505 [105] using the auto parameter and maxiterate set to 1,000. Ambiguously aligned sites were removed using trimAl v1.415 [106] with the “gappyout” option following benchmarking studies [107,108]. Approximate maximum likelihood (ML) phylogenies were inferred from the trimmed alignments using FastTree v2.2.11 with the slow and gamma arguments [109]. Species-specific inparalogs were trimmed using PhyloPyPruner v0.9.5 (https://pypi.org/project/phylopypruner) with the following arguments: “—min-len 50—trim-lb 7—min-support 0.75—min-taxa 35—trim-freq-paralogs 5—prune LS”, resulting in 635 single-copy orthologs. A profile HMM was made for each single-copy ortholog using hmmbuild in HMMER v3.2.1 [110]. The resulting HMMs and orthofisher v1.0.3 [111] were used to identify single-copy orthologs in the 348 proteomes using a fractional bitscore threshold of 0.95.

#### (iii) Tikhonenkov_2020 data matrix

To enhance our analysis, we constructed an additional data matrix using 201 previously identified Opisthokonta orthologs [18]. The study of Tikhonenkov and colleagues [18] focused extensively on the phylogenetic relationships among unicellular holozoans, which are of particular interest in this study. They utilized OrthoFinder for ortholog clustering and subsequently selected the resulting orthologs through a manual curation process, but with a different taxon sampling strategy (55 taxa), providing a valuable opportunity to assess the effects of taxonomic sampling on this segment of the phylogenetic tree. Following this, HMMs were constructed from the multiple sequence alignments using HMMER. Orthofisher was subsequently utilized to pinpoint single-copy orthologs in each proteome.

### Supermatrix construction

Single-copy orthologs from each data set were treated using the same procedure adapted from the PhyloFisher pipeline [112] (Fig 2). Specifically, quality filtering for unaligned single-copy ortholog sequences was done using PREQUAL v1.02 [113] with a 0.95 posterior probability filtering threshold. Filtered sequences were then aligned with MAFFT v7.505 [105] using the argument globalpair, maxiterate set to 1,000, and unalignlevel set to 0.6. Alignments were then processed with Divvier v1.01 [114] using the “divvygap” option and requiring a minimum of 4 characters per column for output. Multiple sequence alignments with lengths less than half of the total alignment length were removed. Highly divergent and gappy sites (>80% gaps) were then trimmed using BMGE v.1.12.2 with default settings [115]. Multiple sequence alignments shorter than 100 bp or with less than 70% taxon representation were removed. Remaining multiple sequence alignments were concatenated using PhyKIT v1.11.10 [92]. The final BUSCO, OrthoFinder, and Tikhonenkov_2020 data matrices contained 228, 440, and 201 genes, respectively, and are represented as BUSCO#1, OrthoFinder#1, and Tikhonenkov_2020#1 (Fig 2 and S2 Table). The overlap between the 3 data matrices was identified using an all-versus-all comparison using DIAMOND [116] with default parameters. Functional categories of each ortholog set in 3 data matrices were annotated using eggNOG v5.0 [117] and BLASTP searches.

### Phylogenomic analysis

To infer the Opisthokonta phylogeny and evaluate the impact of different models on the tree topology, we performed phylogenetic analyses using both site-homogeneous and site-heterogeneous evolutionary models (Fig 2). The site-heterogeneous models were specifically utilized to accommodate varying evolutionary rates across sites, aiming to minimize the impact of LBA. The best-fitting substitution model (LG) was determined using ModelFinder [118] with the option msub set to nuclear. We first inferred phylogenetic trees using the computationally efficient site-homogeneous model LG+I+G4 (hereafter referred to as LG). For site-heterogeneous models, the large size of our data matrices is intractable for the C models [119] and the CAT model [120] implemented in IQ-TREE and PhyloBayes, respectively. However, approximations thereof offer similar benefits and require fewer, but still substantial, resources. Thus, we employed the PMSF (posterior mean site frequency) approximation for these 2 models, which requires a guide tree (inferred using the site-homogenous mode), site-specific stationary distributions, and amino acid exchangeabilities. Approximate site-specific stationary distributions and amino acid exchangeabilities were estimated using the Bayesian GTR+CAT-PMSF model [120,121] (referred to as GTR+CAT) with 1,100 generations and a burn-in of 100 using PhyloBayes-MPI [122] following a previous study [123]. Results were reformatted using publicly available scripts (https://github.com/drenal/cat-pmsf-paper) to be compatible with IQ-TREE 2. Tree inference was then performed in IQ-TREE 2 using the LG+C60+F+G4 model under the PMSF approximation (referred to as LG+C60) [119,124]. All analyses were conducted using unpartitioned models, where the entire data matrix was treated as a single unit without subdividing into separate partitions.

For each data set, branch support was evaluated using ultrafast bootstrap (UFB) replicates. Using 1,000 UFB replicates [125], branch support was binned into 3 categories: strongly supported (above 95), moderately supported (between 90 and 95), and weakly supported (below 90) following a previous study [126]. We constructed single-gene trees for each gene in every data set employing the “-m MFP -msub nuclear” option in IQ-TREE 2. The discordance between the gene trees and the corresponding species tree was quantified using the Robinson–Foulds (RF) distance.

### Molecular dating

To infer the timing of Opisthokonta divergences, we used the Bayesian method MCMCTree in the paml4.9e package [127]. MCMCTree analyses were run on the OrthoFinder#1 data matrix using approximate likelihood calculations with uncorrelated (clock = 2) relaxed clock models and the topology inferred using the LG+C60 model. We used 10 node calibrations based on well-established fossil evidence—7 from Metazoa and 3 from fungi [82,128–132] (S6 Table). To investigate the potential impact of varying root age constraints, 2 alternative ages were established for the root: 1.5 billion years ago and 1.9 billion years ago. For computational tractability, MCMCTree were run on 10 sub-matrices, each consisting of a randomly chosen subset of 100 genes. The MCMC chain was first run for 100,000 iterations as burn-in, then sampled every 500 iterations until a total of 3,000 samples was collected. Lastly, the divergence time estimate for each internal branch was calculated as the average across the timetrees produced by the 10 runs. To analyze historical rates of species accumulation, we utilized the resulting timetree to construct an LTT plot with the APE R package [133].

### Systematically evaluating analytical errors

Phylogenetic inference of deep divergences, such as those concerning major Opisthokonta lineages, are susceptible to many sources of error that may lead to erroneous reconstructions [54,94,134–136]. By prioritizing a subset of genes deemed more dependable, it becomes possible to evaluate contentious branches and disentangle the effects of confounding variables [21,27,33,137] such as missing data and saturation. Specifically, a series of submatrices were generated using an information theory based framework. Subsetting strategies featured subsampling taxa, sites, or genes based on multiple dimensions of information content, such as rogue taxa, long-branch scores (LB scores), rates of sequence evolution, composition heterogeneity (measured by relative composition frequency variability or RCFV) [138,139], missing data, and phylogenetic usefulness (Fig 2). We also tested the effect of taxon sampling on the resolution of unicellular Holozoa using a taxonomy-informed subsampling strategy (Fig 2). The details of data matrices generated in the analyses can be found in S2 Table. To remove potential confounding effects, all the subsetting was conducted on the rogue taxon pruned data matrices (denoted by the suffix “#2”, see below). This process was carried out in parallel, not progressively.

### (i) Rogue taxa—Data matrices #2

A taxon is deemed rogue if it exhibits considerable variability in its placement across bootstrap trees. Removing them allows for the merging of bipartitions that were distinct prior to their exclusion, resulting in a better resolved consensus tree [140]. Rogue taxa were identified in the 3 full data matrices (denoted by the suffix “#1”) using a graph-based algorithm RogueNaRoK [140], revealing *Tunicaraptor unikontum* is a putatively rogue taxon in the OrthoFinder#1 data matrix, but not the other 2 data matrices. This result corroborates previous reports that the placement of *T*. *unikontum* is unstable and its inclusion has a substantial confounding effect on the resolution of early holozoan phylogeny (S1 Data) [18]. Hence, *T*. *unikontum* was pruned from each data matrix (S2 Table), we then performed the same phylogenetic analyses as described above on the resulting data matrices.

### (ii) Long-branch score—Data matrices #3

Removing taxa that exhibit high evolutionary rates, or “long branches,” could help address issues related to heterotachy in phylogenetic analyses [141]. LB scores, a metric that can be used to identify taxa that might cause LBA artifact [142], was calculated for each taxon using PhyKIT [92] following [142]. Lower LB scores are thought to be desirable because they are indicative of taxa or trees that likely do not have issues with LBA. To rigorously identify long-branched taxa, we selected the top 10% of taxa with the highest long-branch scores from each of 3 data sets. We then cross-referenced these selections to identify taxa that consistently appeared in the top 10% across all data sets, thereby defining our long-branched taxa. This analysis identified 27 “long-branched” taxa (S15 Table), which were pruned from the #2 data matrices (Fig 2).

### (iii) Taxon sampling—Data matrices #4–7

To assess the impact of taxon sampling on phylogenetic topologies, 4 submatrices with different taxon sampling densities (while maintaining a high diversity) were generated. To be comparable with the taxon number in previous studies (Torruella and colleagues [19], 83 taxa; Grau-Bove and colleagues [20], 57 taxa; López-Escardó and colleagues [26], 79 taxa; Hehenberger and colleagues [11], 38 taxa; Tikhonenkov and colleagues [18], 75 taxa), 60 taxa representing 25 major lineages in Opisthokonta were selected while preserving the most comprehensive representation of Filasterea, Ichthyosporea, and Pluriformea (S16 Table). The impact of increased taxon sampling was evaluated by randomly selecting additional, nonredundant species from the remaining taxa to create 3 additional data sets of 120, 180, and 240 taxa resulting in 12 new data matrices (Fig 2 and S16 Table), this approach guaranteed each species adds unique value to the phylogenetic analysis. Step size was set at 60 to ensure a uniform and methodical increase from the initial data set.

### (iv) Fast evolving sites—Data matrices #8–10

Fast-evolving sites may suffer from saturation by multiple substitutions and cause LBA artifacts [11]. For each data matrix, 3,000, 6,000, or 9,000 sites with the highest rates of sequence evolution were removed using the fast_site_remover.py script from PhyloFisher [112], which uses DistEst [143] to estimate evolutionary rates. Briefly, site-wise evolutionary rates are estimated by assigning sites to various rate categories based on their evolutionary rates, calculated using a discrete gamma distribution and optimized through maximum-likelihood estimation. This method resulted in a total of 9 new data matrices (Fig 2).

### (v) Phylogenetic usefulness—Data matrices #11–13

Phylogenetic usefulness predicts the performance of genes in phylogenetic analyses based on a principal component axis derived from 7 gene properties: Robinson–Foulds distance; average bootstrap support; saturation; compositional heterogeneity; root to tip variance; average patristic distance; and proportion of variable sites, offering a distinct advantage by not depending on a single gene property or the direct assessment of variables measured [137]. Gene properties related to potential phylogenetic usefulness and bias were calculated using the genesortR package [137]. The 3 data matrices were then subsampled using the best-ranked 90, 80, and 70 percent of genes (Fig 2). These particular thresholds were selected after finding that using less than 50% of the genes led to poorly resolved trees. The goal was to maintain the maximum number of loci while incrementally removing them to examine the impact on the phylogenetic trees.

### (vi) Compositional heterogeneity—Data matrices #14–16

Compositional heterogeneity has been implicated as an important source of systematic error in Opisthokonta phylogeny [14,18,86,89], which could lead to compositional bias and LBA artifacts, potentially skewing phylogenetic results. One way to assess it is using the RCFV score measured from the frequencies of the amino acid in each gene alignment. Reduce compositional heterogeneity in the data matrix could help ameliorate the compositional bias. The 90, 80, and 70 percent of genes with the lowest RCFV scores, indicative of being least prone to compositional biases, were subsampled using genesortR [137] (Fig 2).

### (vii) Missing data—Data matrices #17–19

Missing data are common in data matrices and can result from alignment gaps or the absence of information for certain genes in some species [144]. The effect of such missing data on phylogenetic inference is a subject of ongoing debate. In this study, we assess the impact of missing data by subsampling genes that retain 90%, 80%, and 70% completeness—those with the least amount of missing information—using the genesortR [137] (Fig 2).

### Phylogenetic inference of subsampled data matrices #3–19

We performed ML phylogenetic analyses with IQ-TREE 2 [145] on the subsampled matrices using a single LG model, assessing topological support with 1,000 UFBs [125]. Phylogenetic inference of data matrices #4–7 were further examined using the GTR+CAT model as described above. Support for the 3 alternative topologies (Pluriformea-sister, Teretosporea-sister, and Ichthyosporea-sister hypotheses, Fig 4A) was also examined by examining the frequency of each topology among the 1,000 UFB replicates using IQ-TREE 2. Specifically, cladogram of Pluriformea-sister: (Pluriformea, (Ichthyosporea, Filozoa)), Teretosporea-sister: ((Pluriformea, Ichthyosporea), Filozoa), and Ichthyosporea-sister: (Ichthyosporea, (Pluriformea, Filozoa)) were input to IQ-TREE 2 via the sup option, with the remaining taxa constrained as polytomies.

### Quantifying single-gene phylogenetic signal

Single-gene phylogenetic signal was quantified using 2 approaches: likelihood scores and concordance factors. gCFs and sCFs—the percentage of gene trees that support a node based on descendant taxa and the percentage of informative sites that support that node via parsimony, respectively [146]—were calculated using IQ-TREE 2. To calculate gCFs, individual gene trees were first inferred using IQ-TREE 2 using the best fitting substitution model selected by ModelFinder with the msub parameter set to nuclear, gCFs were then estimated by comparing individual gene trees to the concatenated tree inferred with LG model; sCFs were calculated using 100 random quartets.

To examine phylogenetic signals supporting 2 conflicting hypotheses recovered in this study (Pluriformea-sister and Teretosporea-sister, see Fig 4A), we examined the gene likelihood scores for each data matrix (#2). Site-wise support was calculated for both hypotheses using IQ-TREE 2 with the g option and the LG model. The number of genes supporting each hypothesis was then calculated from IQ-TREE 2 using the wsl option by comparing genewise log-likelihood scores (ΔGLS) [147]. Genes with an absolute value of log-likelihood difference greater than 2 (|ΔGLS| > 2) were considered to have strong phylogenetic signal; those with a difference less than 2 (|ΔGLS| < 2) were considered to have weak signals, following Shen and colleagues [147].

To examine the influence of single genes with high ΔGLS values, each of the data matrices #2 were subsampled by pruning the 1, 5, 10, and 50 genes with the highest absolute ΔGLS values following Shen and colleagues [147], resulting in 12 new data matrices. A species tree was then estimated for each matrix using IQ-TREE 2 with the LG model and 1,000 ultrafast bootstrapping replicates [125].

## Supporting information

S1 Fig Lineage-through-time (LTT) plot for major component groups in Opisthokonta tree of life. The time tree generated using mcmctree was used for lineage-through-time plot using the ltt.plot function in the APE R package [133]. We defined 12 groups: Unicellular holozoans, includes Choanoflagellatea, Filasterea, Ichthyosporea, and Pluriformea; Ctenophora; Porifera; Placozoa; Cnidaria; Deuterostomia: comprises Chordata, Echinodermata, Hemichordata and Xenacoelomorpha; Ecdysozoa: consists of Arthropoda and Tardigrada; Lophotrochozoa: includes Annelida, Mollusca, Nemertea, Bryozoa, and Branchiopoda; Dikarya: include Ascomycota and Basidiomycota; Zygomycetous fungi: This group includes Mucoromycota and Zoopagomycota and Olpidiomycota; Zoosporic fungi: Comprises Blastocladiomycota and Chytridiomycota; “others” include nucleariids and Cryptomycota; The script used to generate this figure is available at https://doi.org/10.6084/m9.figshare.23301824.v1.
(TIFF)

S2 Fig Comparison of the 3 data matrices constructed in this study. (A) Venn diagram of shared orthologs for the 3 data matrices (details of genes shared see S6 and S7 Tables). The venn diagram was generated using jvenn [148]. (B) Single copy orthologs with functional information, the functional category “S: unknown function” was ignored as it does not include functional information. The functional categories of every gene were determined by averaging the annotations of the corresponding cluster members. The data and code underlying this figure can be found in https://doi.org/10.6084/m9.figshare.23301824.v1.
(TIFF)

S3 Fig Hierarchical clustering dendrogram. The data and code underlying this figure can be found in https://doi.org/10.6084/m9.figshare.23301824.v1.
(TIFF)

S1 Table Figure credits. (XLSX)

S2 Table Details of data matrices. (XLSX)

S3 Table Detailed information of the 348 taxa used in this study. (XLSX)

S4 Table Bipartitions shared among phylogenies reconstructed from phylogenetic analysis and sensitivity analysis. (XLSX)

S5 Table Topology summary of the conflicting nodes recovered in our analysis. (XLSX)

S6 Table Calibrations used for dating the Opisthokonta tree of life. (XLSX)

S7 Table Divergence time estimation comparison using different root ages. (XLSX)

S8 Table Shared genes among the 3 full data matrices. (XLSX)

S9 Table The 19 genes shared among 3 data matrices and annotations. (XLSX)

S10 Table The saturation level in 6 data matrices. (XLSX)

S11 Table Bootstrap support values for key nodes in unicellular Holozoa relationships. (XLSX)

S12 Table Gene-wise likelihood scores and the tree supported. (XLSX)

S13 Table Concordance factor statistics. (XLSX)

S14 Table Detailed information about 52 taxa selected to infer the single-copy orthologs using OrthoFinder. (XLSX)

S15 Table The 27 long-branched taxa. (XLSX)

S16 Table Different sampling densities for the taxon subsampling analysis. (XLSX)

S1 Data Topology summary of all produced phylogenies in this study. (PDF)

## Abbreviations

BUSCO: Benchmarking Universal Single-Copy Orthologs
CI: credibility interval
ECM: extracellular matrix
gCF: gene concordance factor
LBA: long-branch attraction
LTT: lineage-through-time
ML: maximum likelihood
pHMM: profile hidden Markov model
PMSF: posterior mean site frequency
RCFV: relative composition frequency variability
sCF: site concordance factor
UFB: ultrafast bootstrap
