# Supplementary figures and images for: A taxon-rich and genome-scale phylogeny of Opisthokonta
Source: PLoS Biol. 2024 Sep 16;22(9):e3002794. doi: 10.1371/journal.pbio.3002794 (PMC11426530; doi:10.1371/journal.pbio.3002794)

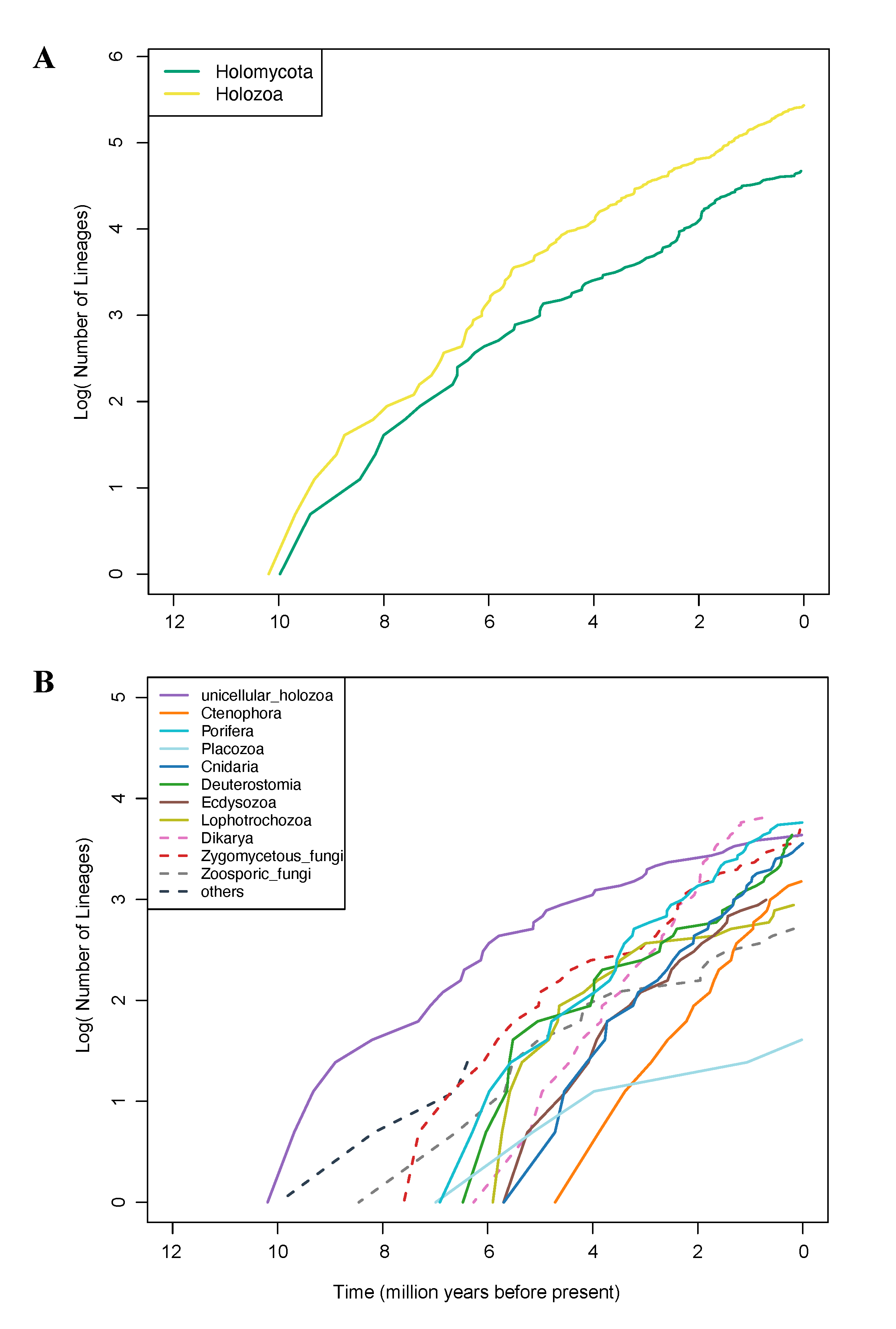

Supplement: S1 Fig — The time tree generated using mcmctree was used for lineage-through-time plot using the ltt.plot function in the APE R package [133]. We defined 12 groups: Unicellular holozoans, includes Choanoflagellatea, Filasterea, Ichthyosporea, and Pluriformea; Ctenophora; Porifera; Placozoa; Cnidaria; Deuterostomia: comprises Chordata, Echinodermata, Hemichordata and Xenacoelomorpha; Ecdysozoa: consists of Arthropoda and Tardigrada; Lophotrochozoa: includes Annelida, Mollusca, Nemertea, Bryozoa, and Branchiopoda; Dikarya: include Ascomycota and Basidiomycota; Zygomycetous fungi: This group includes Mucoromycota and Zoopagomycota and Olpidiomycota; Zoosporic fungi: Comprises Blastocladiomycota and Chytridiomycota; “others” include nucleariids and Cryptomycota; The script used to generate this figure is available at https://doi.org/10.6084/m9.figshare.23301824.v1. (TIFF) [file pbio.3002794.s001.tiff]

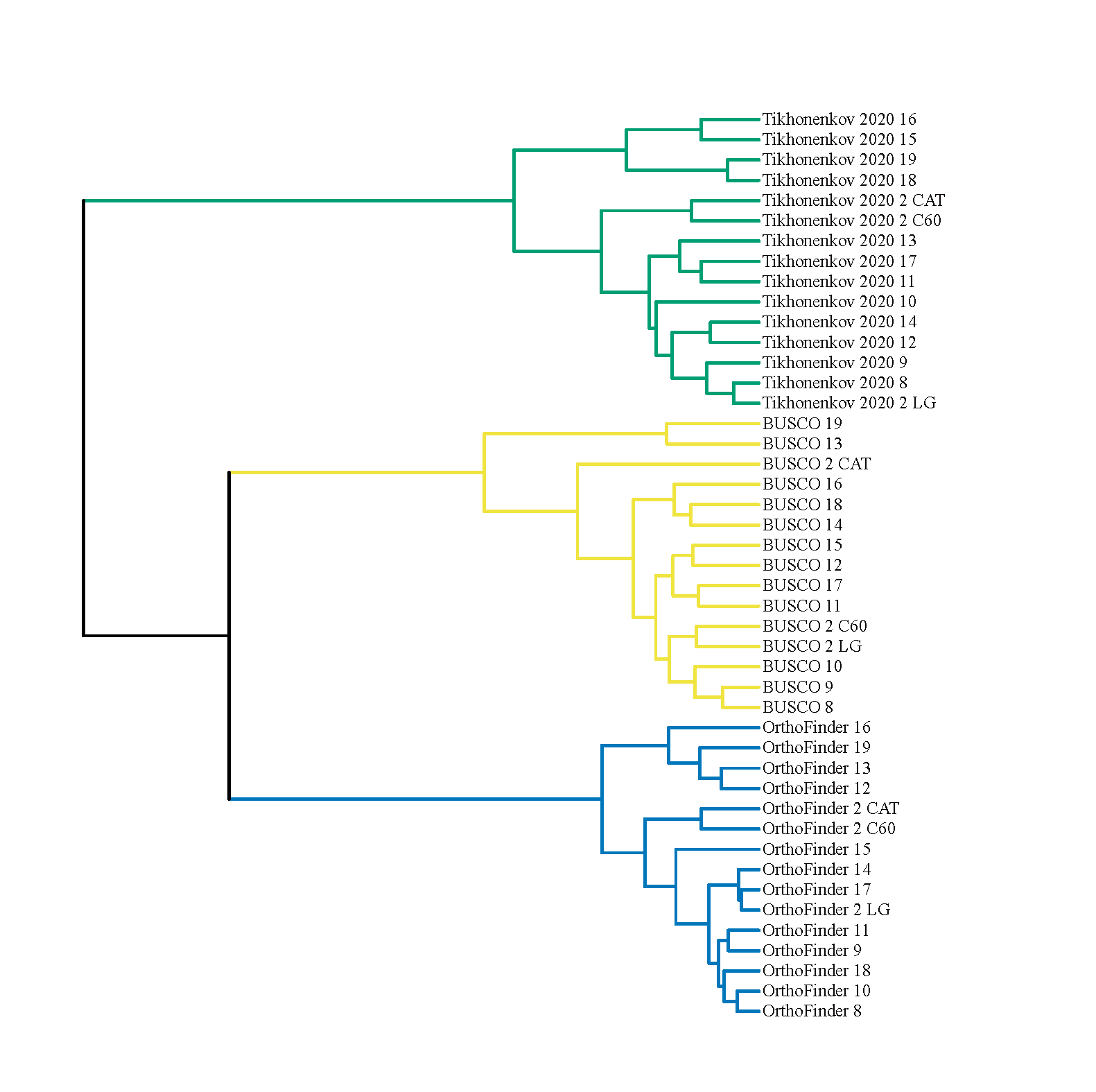

Supplement: S2 Fig — (A) Venn diagram of shared orthologs for the 3 data matrices (details of genes shared see S6 and S7 Tables). The venn diagram was generated using jvenn [148]. (B) Single copy orthologs with functional information, the functional category “S: unknown function” was ignored as it does not include functional information. The functional categories of every gene were determined by averaging the annotations of the corresponding cluster members. The data and code underlying this figure can be found in https://doi.org/10.6084/m9.figshare.23301824.v1. (TIFF) [file pbio.3002794.s002.tiff]

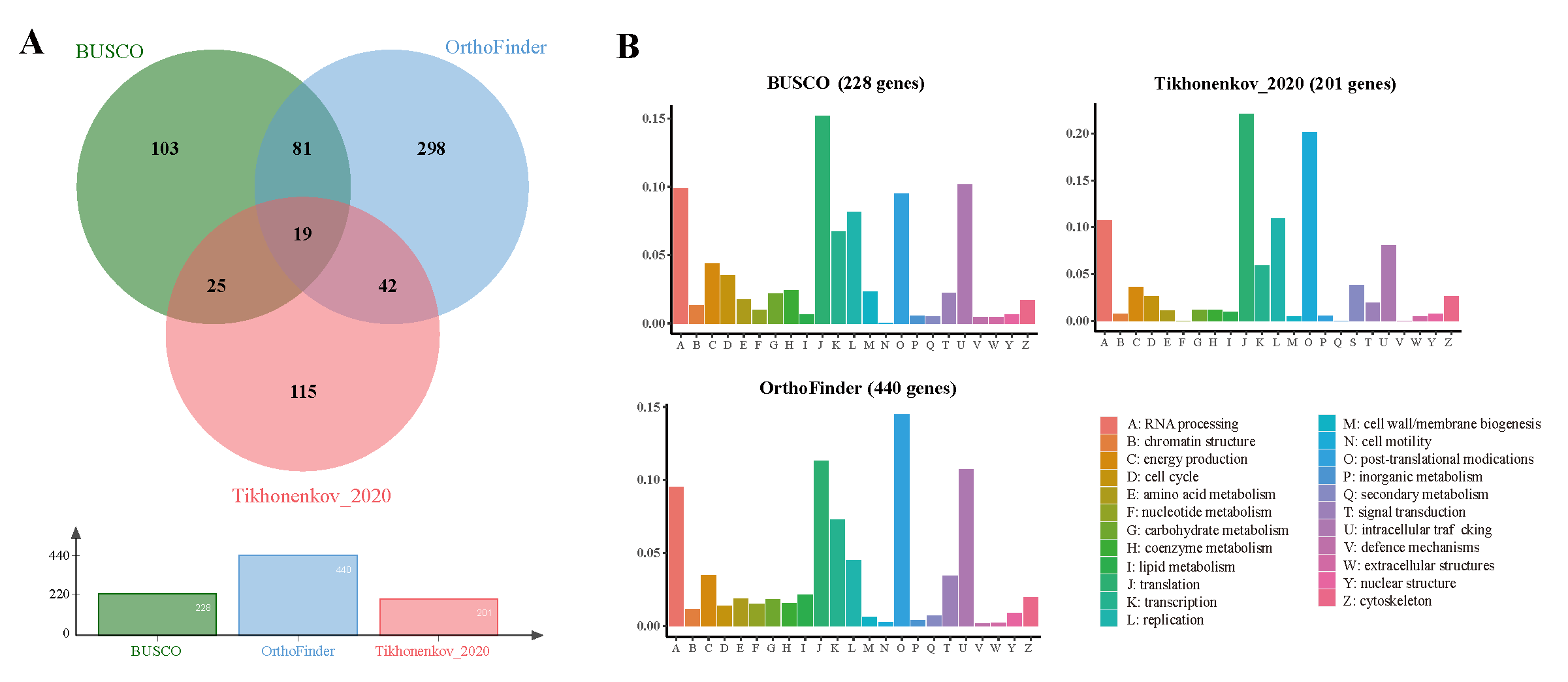

Supplement: S3 Fig — The data and code underlying this figure can be found in https://doi.org/10.6084/m9.figshare.23301824.v1. (TIFF) [file pbio.3002794.s003.tiff]
