# Supplementary material for: A taxon-rich and genome-scale phylogeny of Opisthokonta
Source: PLoS Biol. 2024 Sep 16;22(9):e3002794. doi: 10.1371/journal.pbio.3002794 (PMC11426530; doi:10.1371/journal.pbio.3002794)
Supplement: S1 Data — (PDF) [file pbio.3002794.s020.pdf]

Data S1

All produced phylogenies are provided as supplementary data. For illustration proposes, major lineages were collapsed, branches without designation had bootstrap support values greater than or equal to 95. The original tree files were deposited in Figshare repository (10.6084/m9.figshare.23301824).

1. Topology recovered from ML analyses of the full BUSCO data matrix (#1) under the LG+I+G4 model.

2. Topology recovered from ML analyses of the full BUSCO data matrix (#1) under the C60-PMSF model.

3. Topology recovered from ML analyses of the full BUSCO data matrix (#1) under the GTR+CAT-PMSF model.

4. Topology recovered from ML analyses of the rogue-pruned BUSCO data matrix (#2) under the LG+I+G4 model.

5. Topology recovered from ML analyses of the rogue-pruned BUSCO data matrix (#2) under the C60-PMSF model.

6. Topology recovered from ML analyses of the rogue-pruned BUSCO data matrix (#2) under the GTR+CAT-PMSF model.

7. Topology recovered from ML analyses of the full OrthoFinder data matrix (#1) under the LG+I+G4 model.

8. Topology recovered from ML analyses of the full OrthoFinder data matrix (#1) under the C60-PMSF model.

9. Topology recovered from ML analyses of the full OrthoFinder data matrix (#1) under the GTR+CAT-PMSF model.

10. Topology recovered from ML analyses of the rogue-pruned OrthoFinder data matrix (#2) under the LG+I+G4 model.

11. Topology recovered from ML analyses of the rogue-pruned OrthoFinder data matrix (#2) under the C60-PMSF model.

12. Topology recovered from ML analyses of the rogue-pruned OrthoFinder data matrix (#2) under the GTR+CAT-PMSF model.

13. Topology recovered from ML analyses of the full Tikhonenkov_2020 data matrix (#1) under the LG+I+G4 model.

14. Topology recovered from ML analyses of the full Tikhonenkov_2020 data matrix (#1) under the C60-PMSF model.

15. Topology recovered from ML analyses of the full Tikhonenkov_2020 data matrix (#1) under the GTR+CAT-PMSF model.

16. Topology recovered from ML analyses of the rogue-pruned Tikhonenkov_2020 data matrix (#2) under the LG+I+G4 model.

17. Topology recovered from ML analyses of the rogue-pruned Tikhonenkov_2020 data matrix (#2) under the C60-PMSF model.

18. Topology recovered from ML analyses of the rogue-pruned Tikhonenkov_2020 data matrix (#2) under the GTR+CAT-PMSF model.

19. Topology recovered from ML analyses of the BUSCO data matrix (#3, pruned 27 long-branched taxa) under the LG+I+G4 model.

20. Topology recovered from ML analyses of the OrthoFinder data matrix (#3, pruned 27 long-branched taxa) under the LG+I+G4 model.

21. Topology recovered from ML analyses of the Tikhonenkov_2020 data matrix (#3, pruned 27 long-branched taxa) under the LG+I+G4 model.

22. Topology recovered from ML analyses of the BUSCO data matrix #8 (removed 3000 fast evolving sites) under the LG+I+G4 model.

23. Topology recovered from ML analyses of the BUSCO data matrix #9 (removed 6000 fast evolving sites) under the LG+I+G4 model.

24. Topology recovered from ML analyses of the BUSCO data matrix #10 (removed 9000 fast evolving sites) under the LG+I+G4 model.

25. Topology recovered from ML analyses of the BUSCO data matrix #11 (90% of the most “phylogenetic useful” genes) under the LG+I+G4 model.

26. Topology recovered from ML analyses of the BUSCO data matrix #12 (80% of the most “phylogenetic useful” genes) under the LG+I+G4 model.

27. Topology recovered from ML analyses of the BUSCO data matrix #13 (70% of the most “phylogenetic useful” genes) under the LG+I+G4 model.

28. Topology recovered from ML analyses of the BUSCO data matrix #14 (90% of the genes with the lowest RCFV value) under the LG+I+G4 model.

29. Topology recovered from ML analyses of the BUSCO data matrix #15 (80% of the genes with the lowest RCFV value) under the LG+I+G4 model.

30. Topology recovered from ML analyses of the BUSCO data matrix #16 (70% of the genes with the lowest RCFV value) under the LG+I+G4 model.

31. Topology recovered from ML analyses of the BUSCO data matrix #17 (90% of the genes with the least missing data) under the LG+I+G4 model.

32. Topology recovered from ML analyses of the BUSCO data matrix #18 (90% of the genes with the least missing data) under the LG+I+G4 model.

33. Topology recovered from ML analyses of the BUSCO data matrix #19 (90% of the genes with the least missing data) under the LG+I+G4 model.

34. Topology recovered from ML analyses of the OrthoFinder data matrix #8 (removed 3000 fast evolving sites) under the LG+I+G4 model.

35. Topology recovered from ML analyses of the OrthoFinder data matrix #9 (removed 6000 fast evolving sites) under the LG+I+G4 model.

36. Topology recovered from ML analyses of the OrthoFinder data matrix #10 (removed 9000 fast evolving sites) under the LG+I+G4 model.

37. Topology recovered from ML analyses of the OrthoFinder data matrix #11 (90% of the most “phylogenetic useful” genes) under the LG+I+G4 model.

38. Topology recovered from ML analyses of the OrthoFinder data matrix #12 (80% of the most “phylogenetic useful” genes) under the LG+I+G4 model.

39. Topology recovered from ML analyses of the OrthoFinder data matrix #13 (70% of the most “phylogenetic useful” genes) under the LG+I+G4 model.

40. Topology recovered from ML analyses of the OrthoFinder data matrix #14 (90% of the genes with the lowest RCFV value) under the LG+I+G4 model.

41. Topology recovered from ML analyses of the OrthoFinder data matrix #15 (80% of the genes with the lowest RCFV value) under the LG+I+G4 model.

42. Topology recovered from ML analyses of the OrthoFinder data matrix #16 (70% of the genes with the lowest RCFV value) under the LG+I+G4 model.

43. Topology recovered from ML analyses of the OrthoFinder data matrix #17 (90% of the genes with the least missing data) under the LG+I+G4 model.

44. Topology recovered from ML analyses of the OrthoFinder data matrix #18 (90% of the genes with the least missing data) under the LG+I+G4 model.

45. Topology recovered from ML analyses of the OrthoFinder data matrix #19 (90% of the genes with the least missing data) under the LG+I+G4 model.

46. Topology recovered from ML analyses of the Tikhonenkov_2020 data matrix #8 (removed 3000 fast evolving sites) under the LG+I+G4 model.

47. Topology recovered from ML analyses of the Tikhonenkov_2020 data matrix #9 (removed 6000 fast evolving sites) under the LG+I+G4 model.

48. Topology recovered from ML analyses of the Tikhonenkov_2020 data matrix #10 (removed 9000 fast evolving sites) under the LG+I+G4 model.

49. Topology recovered from ML analyses of the Tikhonenkov_2020 data matrix #11 (90% of the most "phylogenetic useful" genes) under the LG+I+G4 model.

50. Topology recovered from ML analyses of the Tikhonenkov_2020 data matrix #12 (80% of the most "phylogenetic useful" genes) under the LG+I+G4 model.

51. Topology recovered from ML analyses of the Tikhonenkov_2020 data matrix #13 (70% of the most "phylogenetic useful" genes) under the LG+I+G4 model.

52. Topology recovered from ML analyses of the Tikhonenkov_2020 data matrix #14 (90% of the genes with the lowest RCFV value) under the LG+I+G4 model.

53. Topology recovered from ML analyses of the Tikhonenkov_2020 data matrix #15 (80% of the genes with the lowest RCFV value) under the LG+I+G4 model.

54. Topology recovered from ML analyses of the Tikhonenkov_2020 data matrix #16 (70% of the genes with the lowest RCFV value) under the LG+I+G4 model.

55. Topology recovered from ML analyses of the Tikhonenkov_2020 data matrix #17 (90% of the genes with the least missing data) under the LG+I+G4 model.

56. Topology recovered from ML analyses of the Tikhonenkov_2020 data matrix #18 (90% of the genes with the least missing data) under the LG+I+G4 model.

57. Topology recovered from ML analyses of the Tikhonenkov_2020 data matrix #19 (90% of the genes with the least missing data) under the LG+I+G4 model.

58. Topology recovered from ML analyses of the BUSCO data matrix #4 (60 taxa) under the GTR+CAT-PMSF model.

59. Topology recovered from ML analyses of the BUSCO data matrix #5 (120 taxa) under the GTR+CAT-PMSF model.

60. Topology recovered from ML analyses of the BUSCO data matrix #6 (180 taxa) under the GTR+CAT-PMSF model.

61. Topology recovered from ML analyses of the BUSCO data matrix #7 (240 taxa) under the GTR+CAT-PMSF model.

62. Topology recovered from ML analyses of the OrthoFinder data matrix #4 (60 taxa) under the GTR+CAT-PMSF model.

63. Topology recovered from ML analyses of the OrthoFinder data matrix #5 (120 taxa) under the GTR+CAT-PMSF model.

64. Topology recovered from ML analyses of the OrthoFinder data matrix #6 (180 taxa) under the GTR+CAT-PMSF model.

65. Topology recovered from ML analyses of the OrthoFinder data matrix #7 (240 taxa) under the GTR+CAT-PMSF model.

66. Topology recovered from ML analyses of the Tikhonenkov_2020 data matrix #4 (60 taxa) under the GTR+CAT-PMSF model.

67. Topology recovered from ML analyses of the Tikhonenkov_2020 data matrix #5 (120 taxa) under the GTR+CAT-PMSF model.

68. Topology recovered from ML analyses of the Tikhonenkov_2020 matrix #6 (180 taxa) under the GTR+CAT-PMSF model.

69. Topology recovered from ML analyses of the Tikhonenkov_2020 matrix #7 (240 taxa) under the GTR+CAT-PMSF model.

70. Topology recovered from ML analyses of the BUSCO#2 data matrix removed the remote outgroups.

71. Topology recovered from ML analyses of the OrthoFinder#2 data matrix removed the remote outgroups.

72. Topology recovered from ML analyses of the Tikhonenkov_2020#2 data matrix removed the remote outgroups.

73. Molecular time tree for the Opisthokonta (set the root age to 1.5 billion years), blue boxes around each internode correspond to 95% divergence time confidence intervals for each branch of the Opisthokonta phylogeny.

74. Molecular time tree for the Opisthokonta (set the root age to 1.9 billion years), blue boxes around each internode correspond to 95% divergence time confidence intervals for each branch of the Opisthokonta phylogeny.

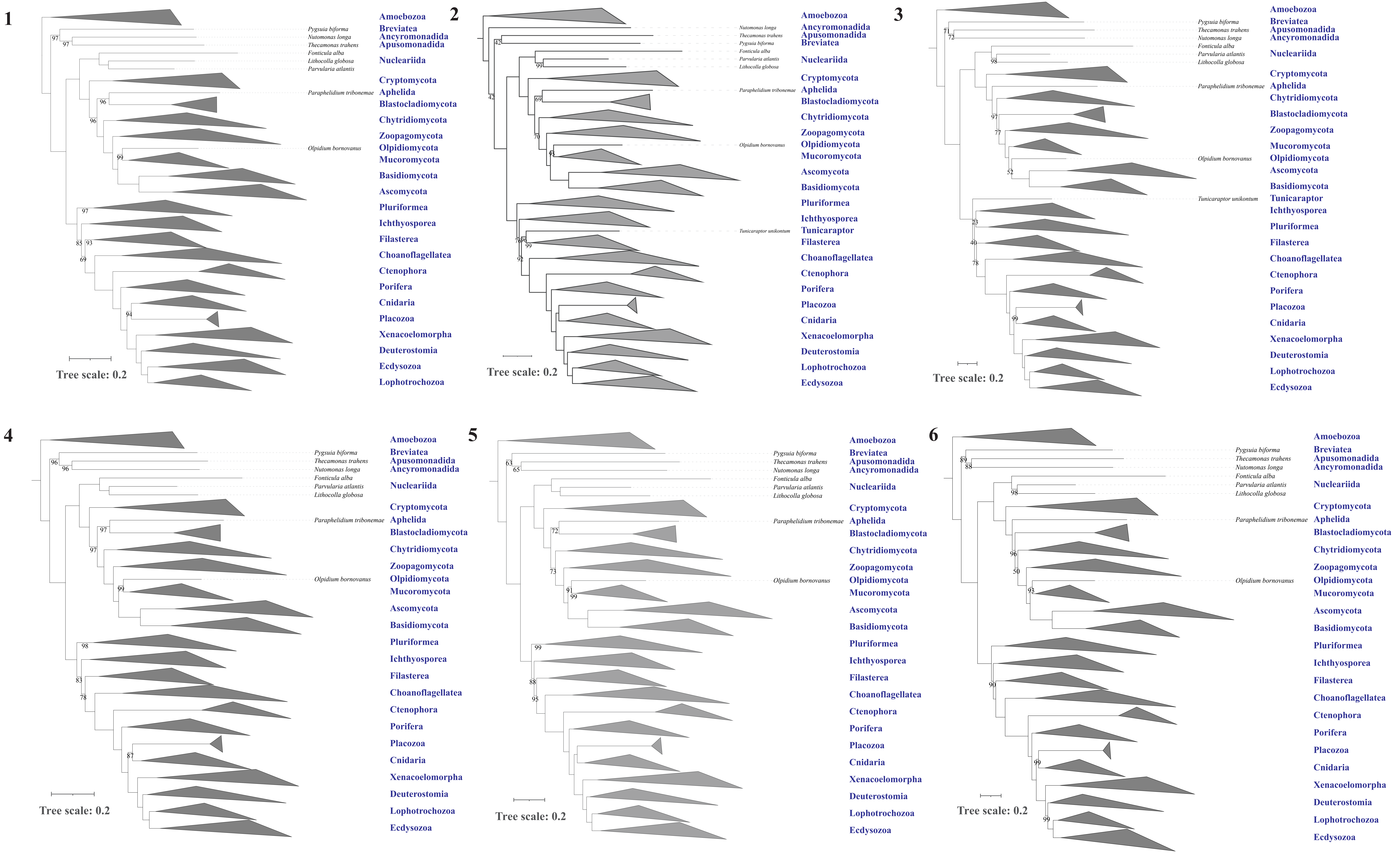
1
Amoebozoa
Breviatea
Ancyromonadida
Apusomonadida
Nucleariida
Cryptomycota
Aphelida
Blastocladiomycota
Chytridiomycota
Zoopagomycota
Olpidiomycota
Mucoromycota
Basidiomycota
Ascomycota
Pluriformea
Ichthyosporea
Filasterea
Choanoflagellatea
Ctenophora
Porifera
Cnidaria
Placozoa
Xenacoelomorpha
Deuterostomia
Ecdysozoa
Lophotrochozoa
Pygsuia biforma
Nutomonas longa
Thecamonas trahens
Fonticula alba
Lithocolla globosa
Parvularia atlantis
Paraphelidium tribonemae
Olpidium bornovanus
Tree scale: 0.2
2
Amoebozoa
Ancyromonadida
Apusomonadida
Breviatea
Nucleariida
Cryptomycota
Aphelida
Blastocladiomycota
Chytridiomycota
Zoopagomycota
Olpidiomycota
Mucoromycota
Ascomycota
Basidiomycota
Pluriformea
Ichthyosporea
Tunicaraptor
Filasterea
Choanoflagellatea
Ctenophora
Porifera
Placozoa
Cnidaria
Xenacoelomorpha
Deuterostomia
Lophotrochozoa
Ecdysozoa
Tunicaraptor unikontum
Tree scale: 0.2
3
Amoebozoa
Breviatea
Apusomonadida
Ancyromonadida
Nucleariida
Cryptomycota
Aphelida
Chytridiomycota
Blastocladiomycota
Zoopagomycota
Mucoromycota
Olpidiomycota
Ascomycota
Basidiomycota
Tunicaraptor
Ichthyosporea
Pluriformea
Filasterea
Choanoflagellatea
Ctenophora
Porifera
Placozoa
Cnidaria
Xenacoelomorpha
Deuterostomia
Lophotrochozoa
Ecdysozoa
Tree scale: 0.2
4
Amoebozoa
Breviatea
Apusomonadida
Ancyromonadida
Nucleariida
Cryptomycota
Aphelida
Blastocladiomycota
Chytridiomycota
Zoopagomycota
Olpidiomycota
Mucoromycota
Ascomycota
Basidiomycota
Pluriformea
Ichthyosporea
Filasterea
Choanoflagellatea
Ctenophora
Porifera
Placozoa
Cnidaria
Xenacoelomorpha
Deuterostomia
Lophotrochozoa
Ecdysozoa
Tree scale: 0.2
5
Tree scale: 0.2
6
Tree scale: 0.2

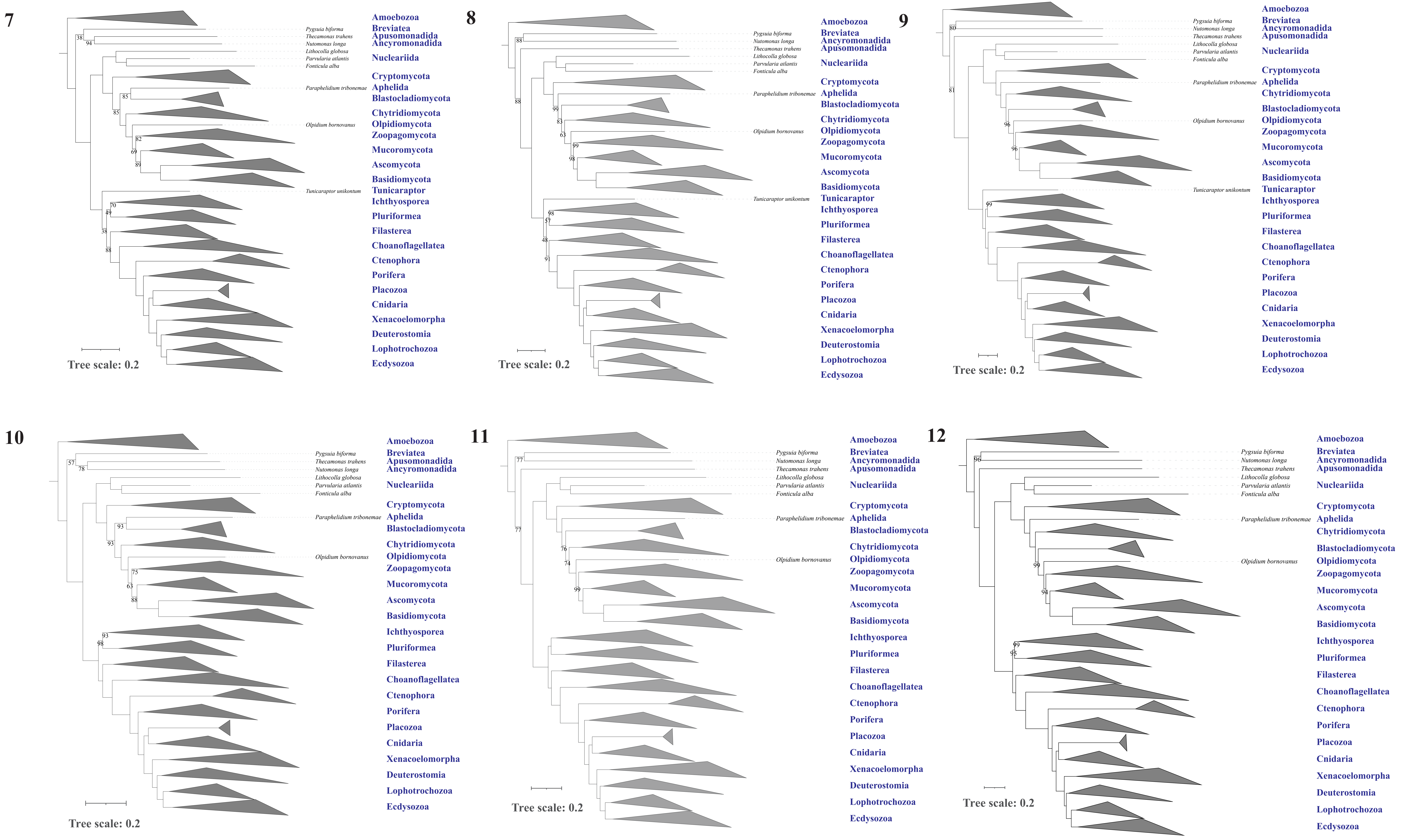
7
Pygsuia biforma
Thecamonas trahens
Nutomonas longa
Lithocolla globosa
Parvularia atlantis
Fonticula alba
Paraphelidium tribonemae
Olpidium bornovanus
Tunicaraptor unikontum
Amoebozoa
Breviatea
Apusomonadida
Ancyromonadida
Nucleariida
Cryptomycota
Aphelida
Blastocladiomycota
Chytridiomycota
Olpidiomycota
Zoopagomycota
Mucoromycota
Ascomycota
Basidiomycota
Tunicaraptor
Ichthyosporea
Pluriformea
Filasterea
Choanoflagellatea
Ctenophora
Porifera
Placozoa
Cnidaria
Xenacoelomorpha
Deuterostomia
Lophotrochozoa
Ecdysozoa
Tree scale: 0.2
8
Pygsuia biforma
Nutomonas longa
Thecamonas trahens
Lithocolla globosa
Parvularia atlantis
Fonticula alba
Paraphelidium tribonemae
Olpidium bornovanus
Tunicaraptor unikontum
Amoebozoa
Breviatea
Ancyromonadida
Apusomonadida
Nucleariida
Cryptomycota
Aphelida
Blastocladiomycota
Chytridiomycota
Olpidiomycota
Zoopagomycota
Mucoromycota
Ascomycota
Basidiomycota
Tunicaraptor
Ichthyosporea
Pluriformea
Filasterea
Choanoflagellatea
Ctenophora
Porifera
Placozoa
Cnidaria
Xenacoelomorpha
Deuterostomia
Lophotrochozoa
Ecdysozoa
Tree scale: 0.2
9
Pygsuia biforma
Nutomonas longa
Thecamonas trahens
Lithocolla globosa
Parvularia atlantis
Fonticula alba
Paraphelidium tribonemae
Olpidium bornovanus
Tunicaraptor unikontum
Amoebozoa
Breviatea
Ancyromonadida
Apusomonadida
Nucleariida
Cryptomycota
Aphelida
Chytridiomycota
Blastocladiomycota
Olpidiomycota
Zoopagomycota
Mucoromycota
Ascomycota
Basidiomycota
Tunicaraptor
Ichthyosporea
Pluriformea
Filasterea
Choanoflagellatea
Ctenophora
Porifera
Placozoa
Cnidaria
Xenacoelomorpha
Deuterostomia
Lophotrochozoa
Ecdysozoa
Tree scale: 0.2
10
Pygsuia biforma
Thecamonas trahens
Nutomonas longa
Lithocolla globosa
Parvularia atlantis
Fonticula alba
Paraphelidium tribonemae
Olpidium bornovanus
Amoebozoa
Breviatea
Apusomonadida
Ancyromonadida
Nucleariida
Cryptomycota
Aphelida
Blastocladiomycota
Chytridiomycota
Olpidiomycota
Zoopagomycota
Mucoromycota
Ascomycota
Basidiomycota
Ichthyosporea
Pluriformea
Filasterea
Choanoflagellatea
Ctenophora
Porifera
Placozoa
Cnidaria
Xenacoelomorpha
Deuterostomia
Lophotrochozoa
Ecdysozoa
Tree scale: 0.2
11
Pygsuia biforma
Nutomonas longa
Thecamonas trahens
Lithocolla globosa
Parvularia atlantis
Fonticula alba
Paraphelidium tribonemae
Olpidium bornovanus
Amoebozoa
Breviatea
Ancyromonadida
Apusomonadida
Nucleariida
Cryptomycota
Aphelida
Blastocladiomycota
Chytridiomycota
Olpidiomycota
Zoopagomycota
Mucoromycota
Ascomycota
Basidiomycota
Ichthyosporea
Pluriformea
Filasterea
Choanoflagellatea
Ctenophora
Porifera
Placozoa
Cnidaria
Xenacoelomorpha
Deuterostomia
Lophotrochozoa
Ecdysozoa
Tree scale: 0.2
12
Pygsuia biforma
Nutomonas longa
Thecamonas trahens
Lithocolla globosa
Parvularia atlantis
Fonticula alba
Paraphelidium tribonemae
Olpidium bornovanus
Amoebozoa
Breviatea
Ancyromonadida
Apusomonadida
Nucleariida
Cryptomycota
Aphelida
Chytridiomycota
Blastocladiomycota
Olpidiomycota
Zoopagomycota
Mucoromycota
Ascomycota
Basidiomycota
Ichthyosporea
Pluriformea
Filasterea
Choanoflagellatea
Ctenophora
Porifera
Placozoa
Cnidaria
Xenacoelomorpha
Deuterostomia
Lophotrochozoa
Ecdysozoa
Tree scale: 0.2

13

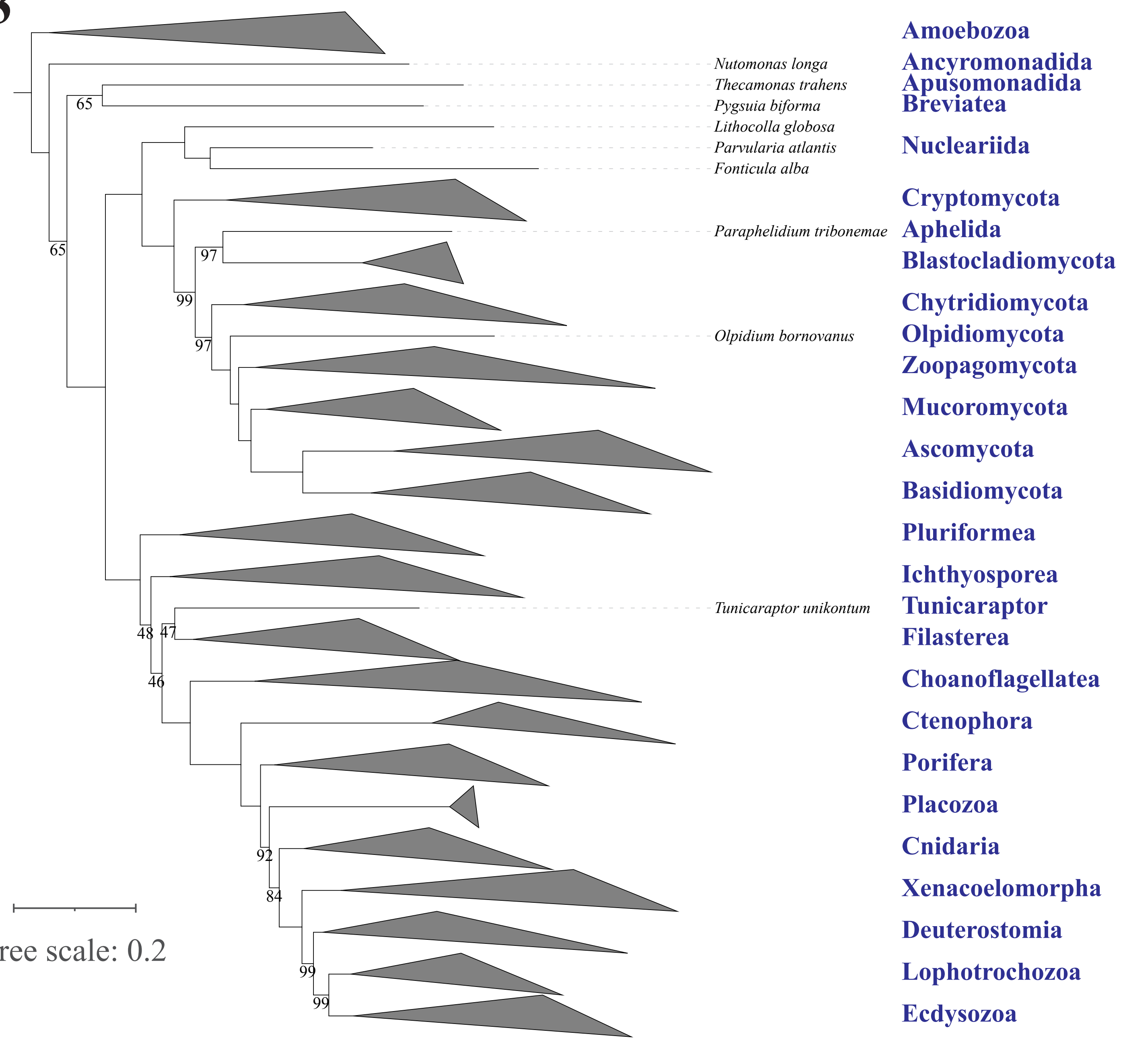

14

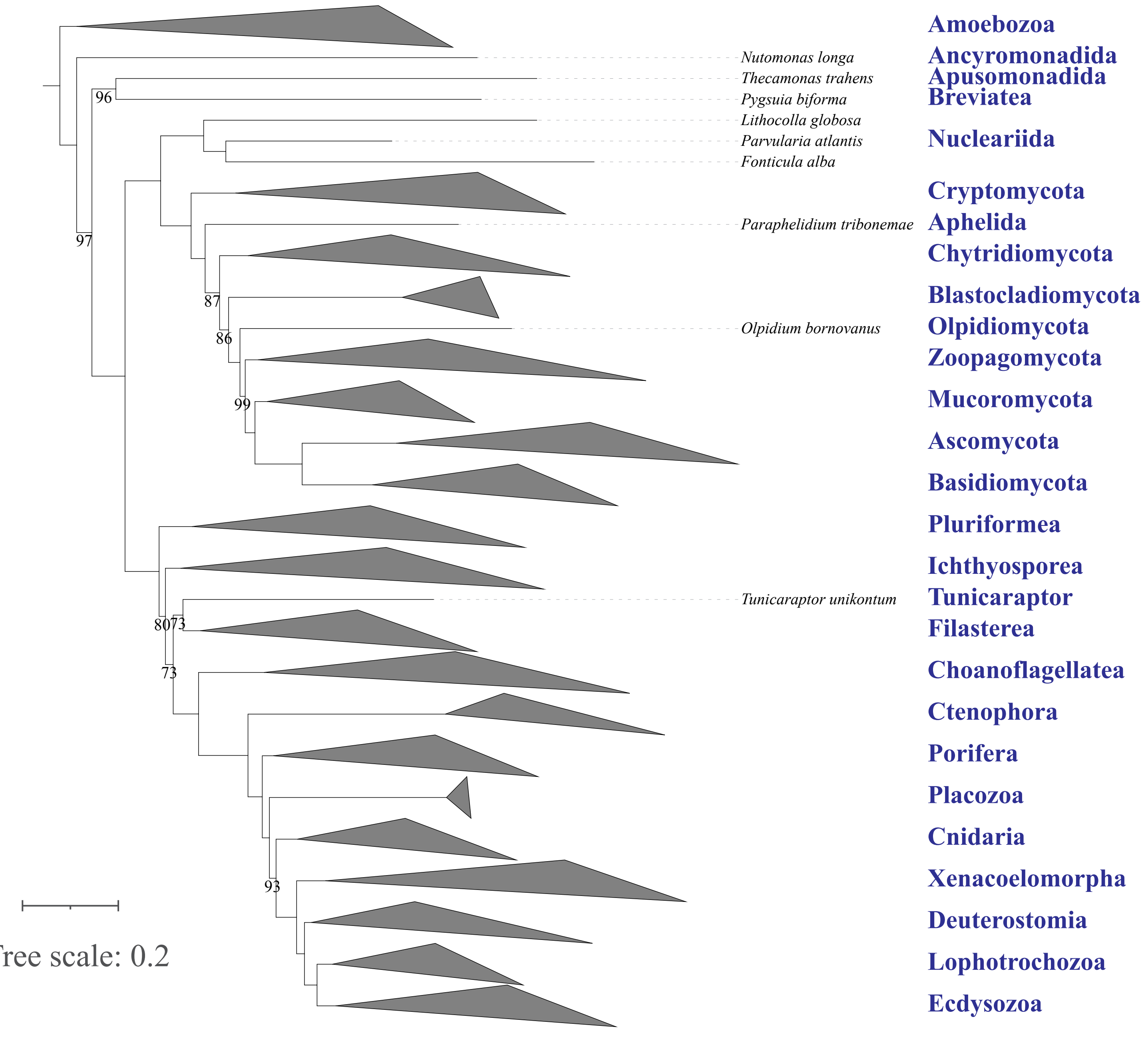

15

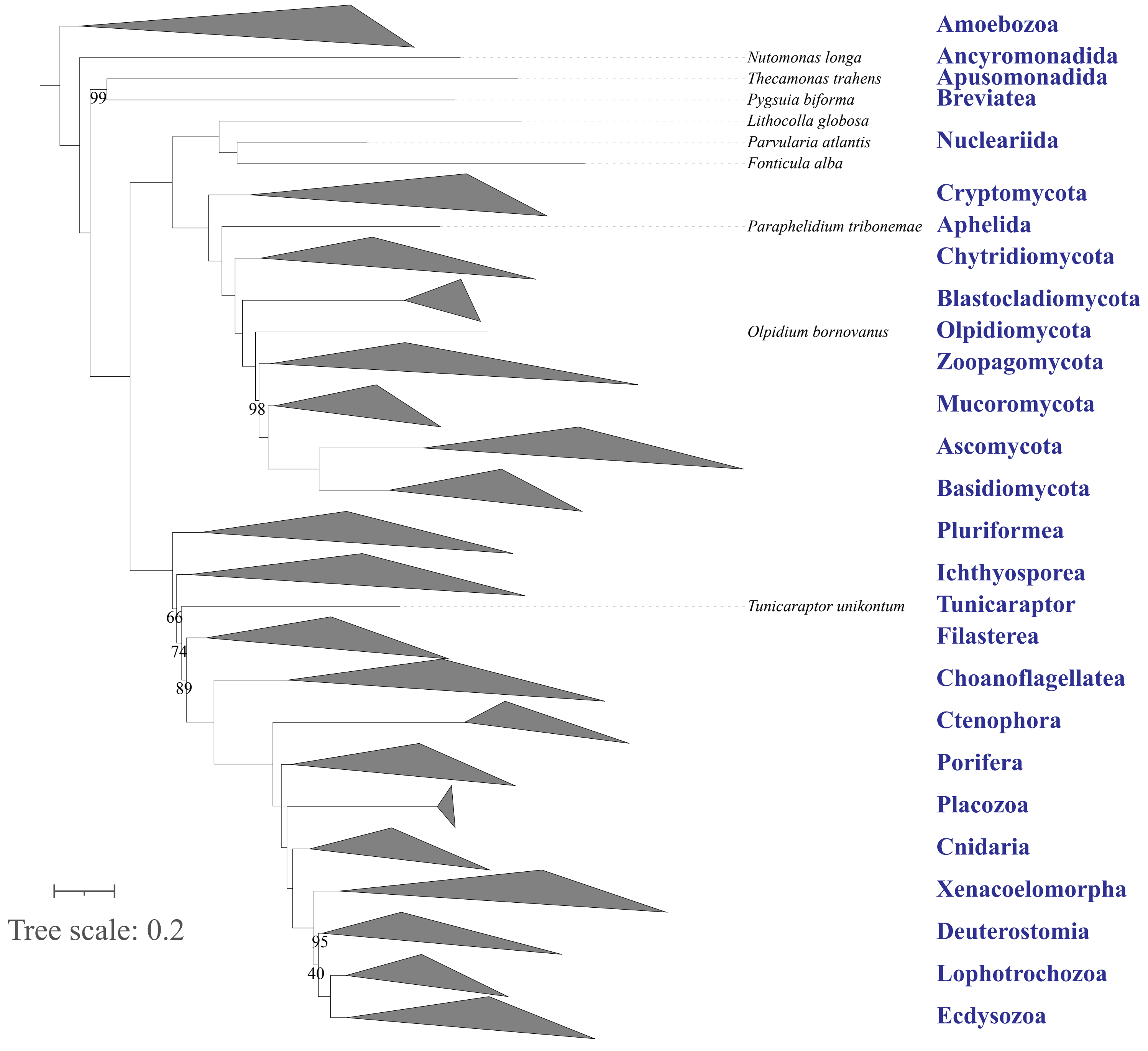

16

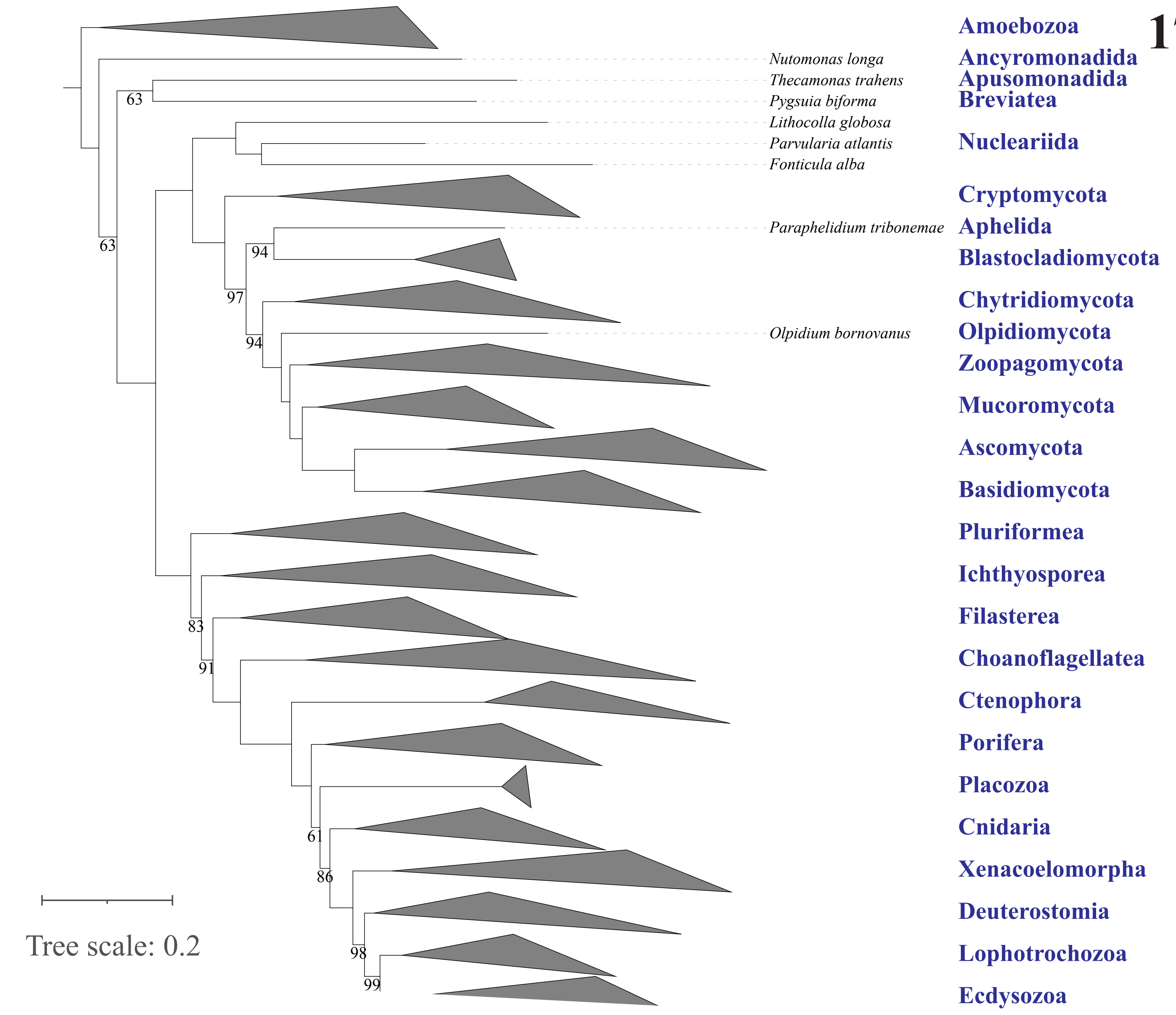

17

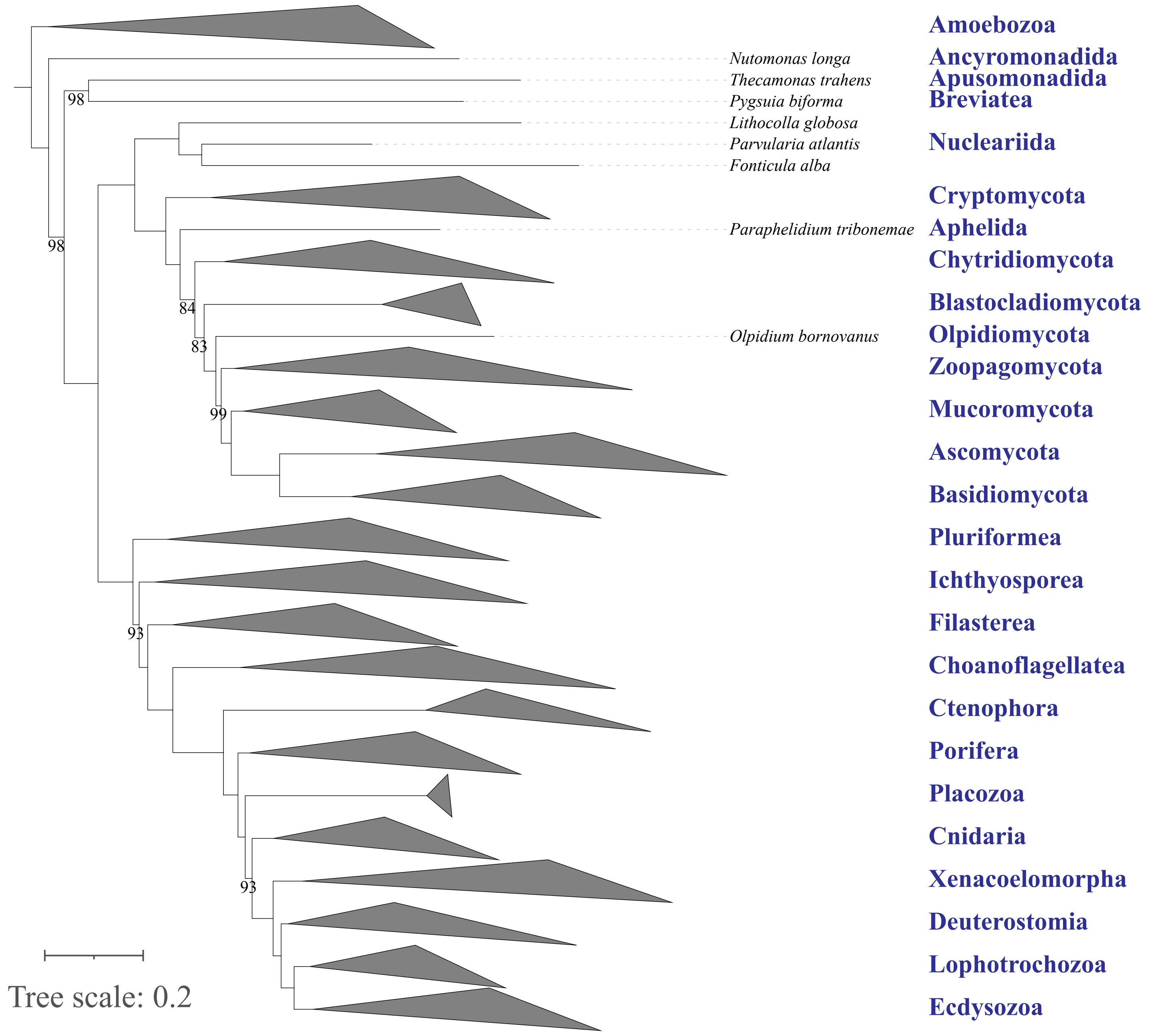

18

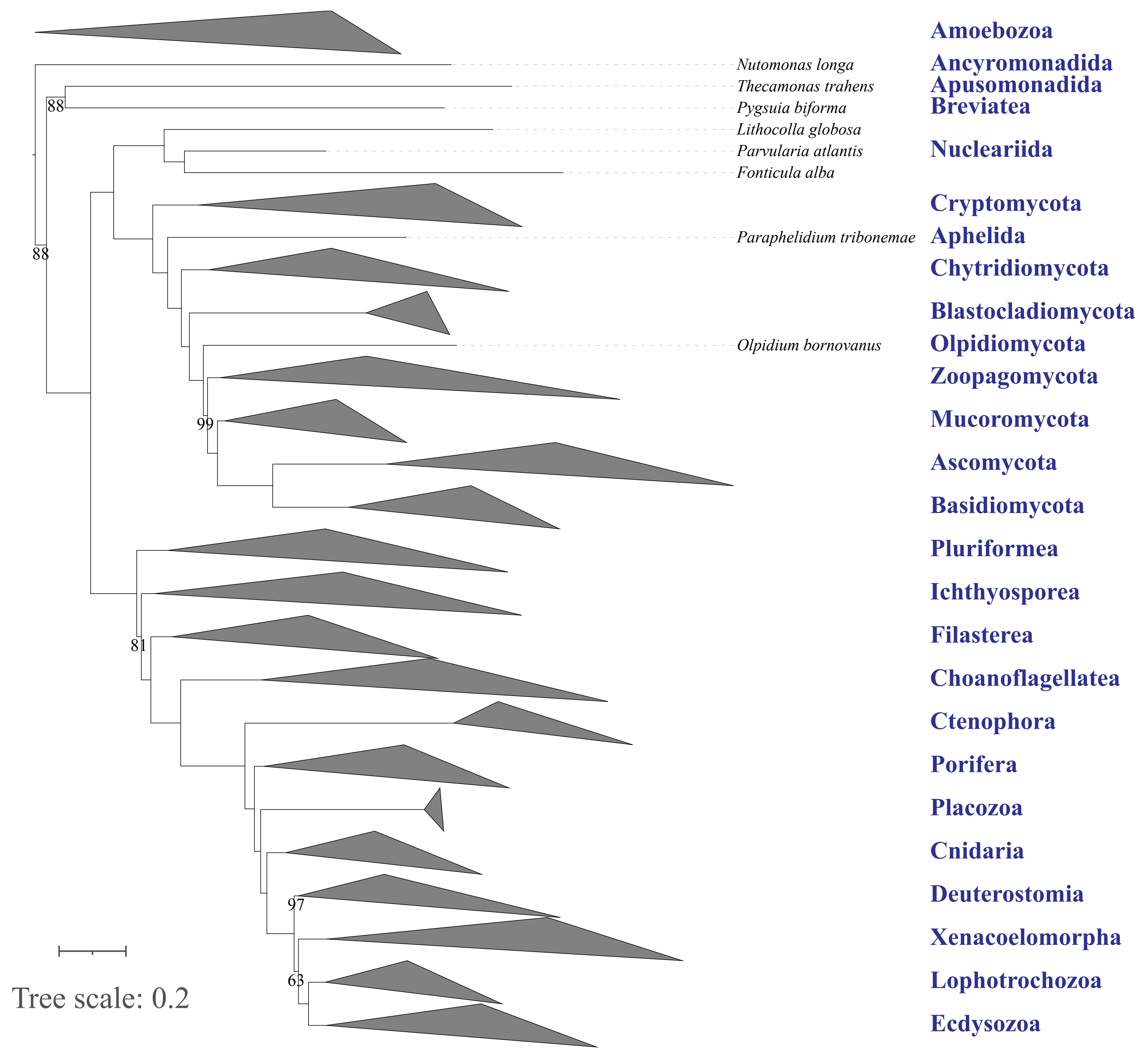

19

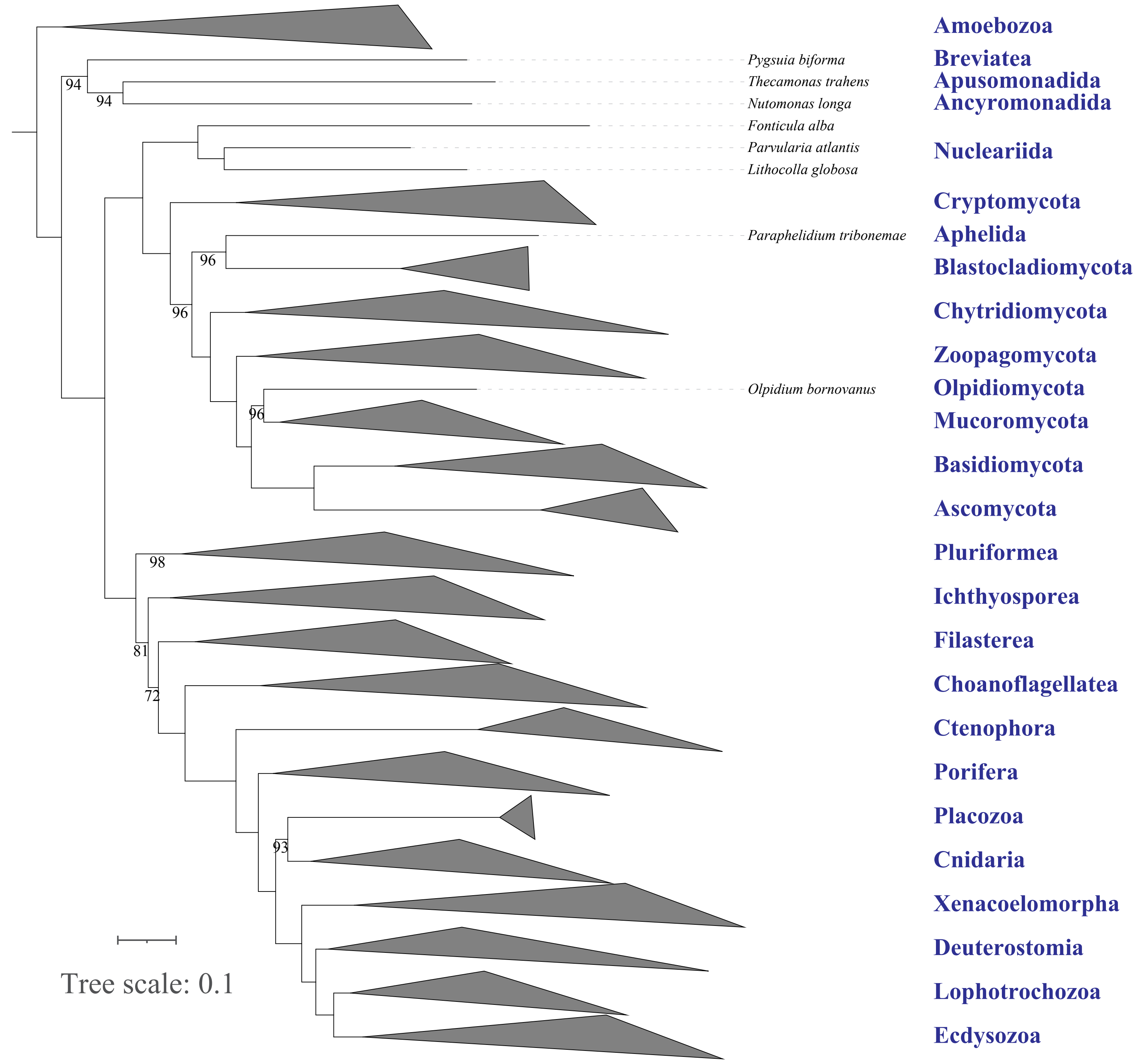

20

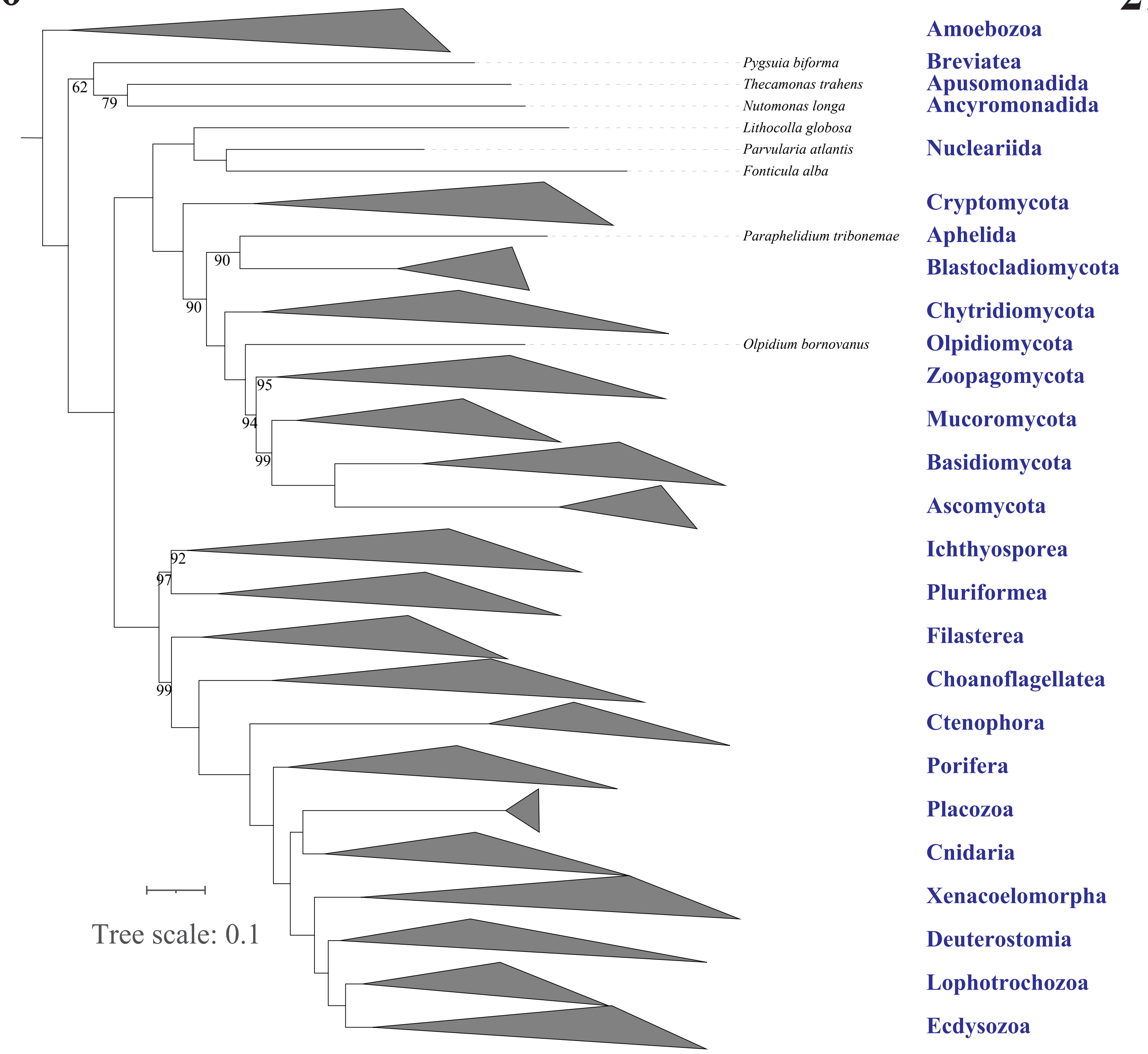

21

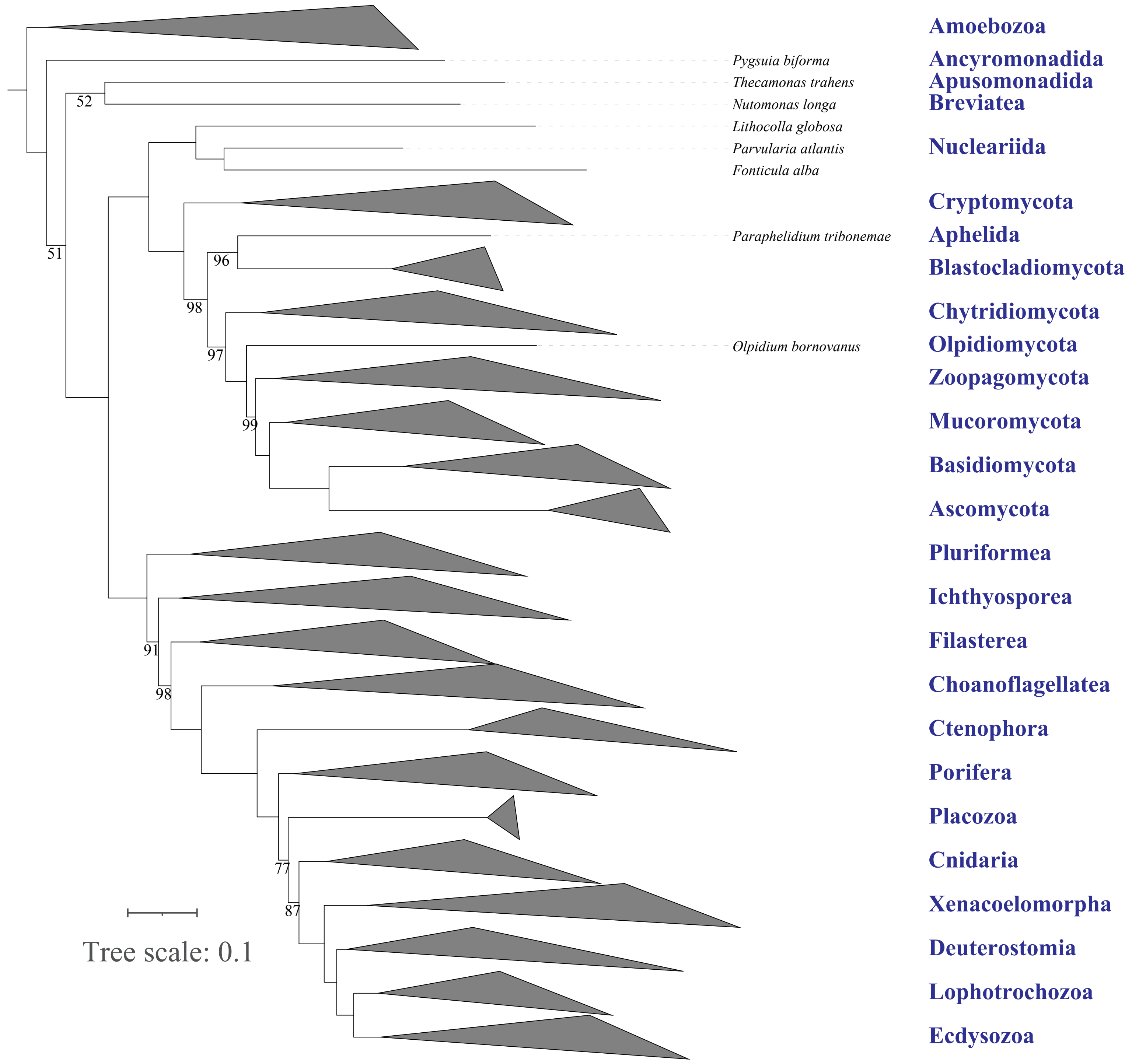

BUSCO LB

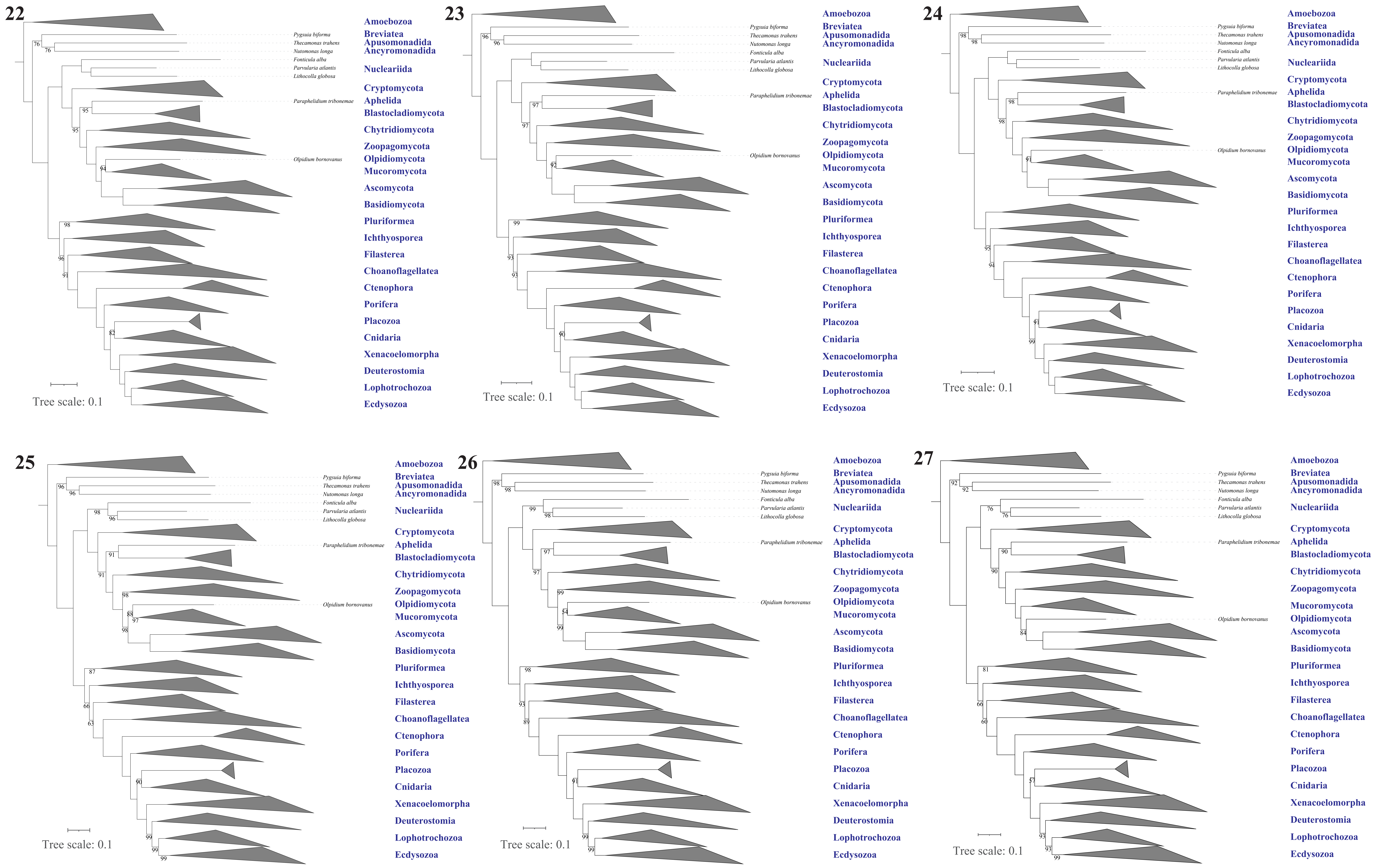
22
Pygsuia biforma
Thecamonas trahens
Nutomonas longa
Fonticula alba
Parvularia atlantis
Lithocolla globosa
Paraphelidium tribonemae
Olpidium bornovanus
Amoebozoa
Breviatea
Apusomonadida
Ancyromonadida
Nucleariida
Cryptomycota
Aphelida
Blastocladiomycota
Chytridiomycota
Zoopagomycota
Olpidiomycota
Mucoromycota
Ascomycota
Basidiomycota
Pluriformea
Ichthyosporea
Filasterea
Choanoflagellatea
Ctenophora
Porifera
Placozoa
Cnidaria
Xenacoelomorpha
Deuterostomia
Lophotrochozoa
Ecdysozoa
Tree scale: 0.1
23
Pygsuia biforma
Thecamonas trahens
Nutomonas longa
Fonticula alba
Parvularia atlantis
Lithocolla globosa
Paraphelidium tribonemae
Olpidium bornovanus
Amoebozoa
Breviatea
Apusomonadida
Ancyromonadida
Nucleariida
Cryptomycota
Aphelida
Blastocladiomycota
Chytridiomycota
Zoopagomycota
Olpidiomycota
Mucoromycota
Ascomycota
Basidiomycota
Pluriformea
Ichthyosporea
Filasterea
Choanoflagellatea
Ctenophora
Porifera
Placozoa
Cnidaria
Xenacoelomorpha
Deuterostomia
Lophotrochozoa
Ecdysozoa
Tree scale: 0.1
24
Pygsuia biforma
Thecamonas trahens
Nutomonas longa
Fonticula alba
Parvularia atlantis
Lithocolla globosa
Paraphelidium tribonemae
Olpidium bornovanus
Amoebozoa
Breviatea
Apusomonadida
Ancyromonadida
Nucleariida
Cryptomycota
Aphelida
Blastocladiomycota
Chytridiomycota
Zoopagomycota
Olpidiomycota
Mucoromycota
Ascomycota
Basidiomycota
Pluriformea
Ichthyosporea
Filasterea
Choanoflagellatea
Ctenophora
Porifera
Placozoa
Cnidaria
Xenacoelomorpha
Deuterostomia
Lophotrochozoa
Ecdysozoa
Tree scale: 0.1
25
Pygsuia biforma
Thecamonas trahens
Nutomonas longa
Fonticula alba
Parvularia atlantis
Lithocolla globosa
Paraphelidium tribonemae
Olpidium bornovanus
Amoebozoa
Breviatea
Apusomonadida
Ancyromonadida
Nucleariida
Cryptomycota
Aphelida
Blastocladiomycota
Chytridiomycota
Zoopagomycota
Olpidiomycota
Mucoromycota
Ascomycota
Basidiomycota
Pluriformea
Ichthyosporea
Filasterea
Choanoflagellatea
Ctenophora
Porifera
Placozoa
Cnidaria
Xenacoelomorpha
Deuterostomia
Lophotrochozoa
Ecdysozoa
Tree scale: 0.1
26
Pygsuia biforma
Thecamonas trahens
Nutomonas longa
Fonticula alba
Parvularia atlantis
Lithocolla globosa
Paraphelidium tribonemae
Olpidium bornovanus
Amoebozoa
Breviatea
Apusomonadida
Ancyromonadida
Nucleariida
Cryptomycota
Aphelida
Blastocladiomycota
Chytridiomycota
Zoopagomycota
Olpidiomycota
Mucoromycota
Ascomycota
Basidiomycota
Pluriformea
Ichthyosporea
Filasterea
Choanoflagellatea
Ctenophora
Porifera
Placozoa
Cnidaria
Xenacoelomorpha
Deuterostomia
Lophotrochozoa
Ecdysozoa
Tree scale: 0.1
27
Pygsuia biforma
Thecamonas trahens
Nutomonas longa
Fonticula alba
Parvularia atlantis
Lithocolla globosa
Paraphelidium tribonemae
Olpidium bornovanus
Amoebozoa
Breviatea
Apusomonadida
Ancyromonadida
Nucleariida
Cryptomycota
Aphelida
Blastocladiomycota
Chytridiomycota
Zoopagomycota
Mucoromycota
Olpidiomycota
Ascomycota
Basidiomycota
Pluriformea
Ichthyosporea
Filasterea
Choanoflagellatea
Ctenophora
Porifera
Placozoa
Cnidaria
Xenacoelomorpha
Deuterostomia
Lophotrochozoa
Ecdysozoa
Tree scale: 0.1

28

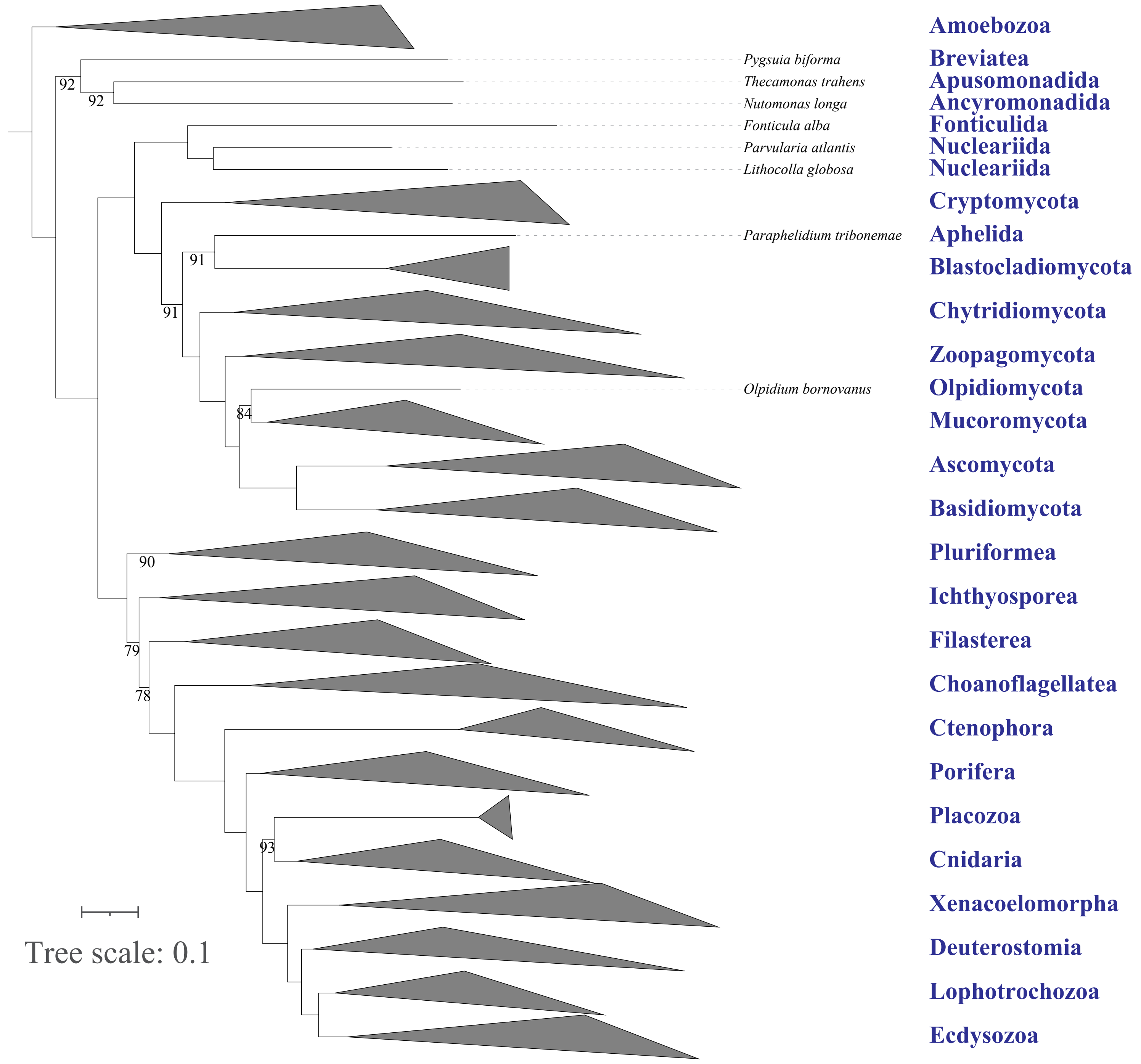

29

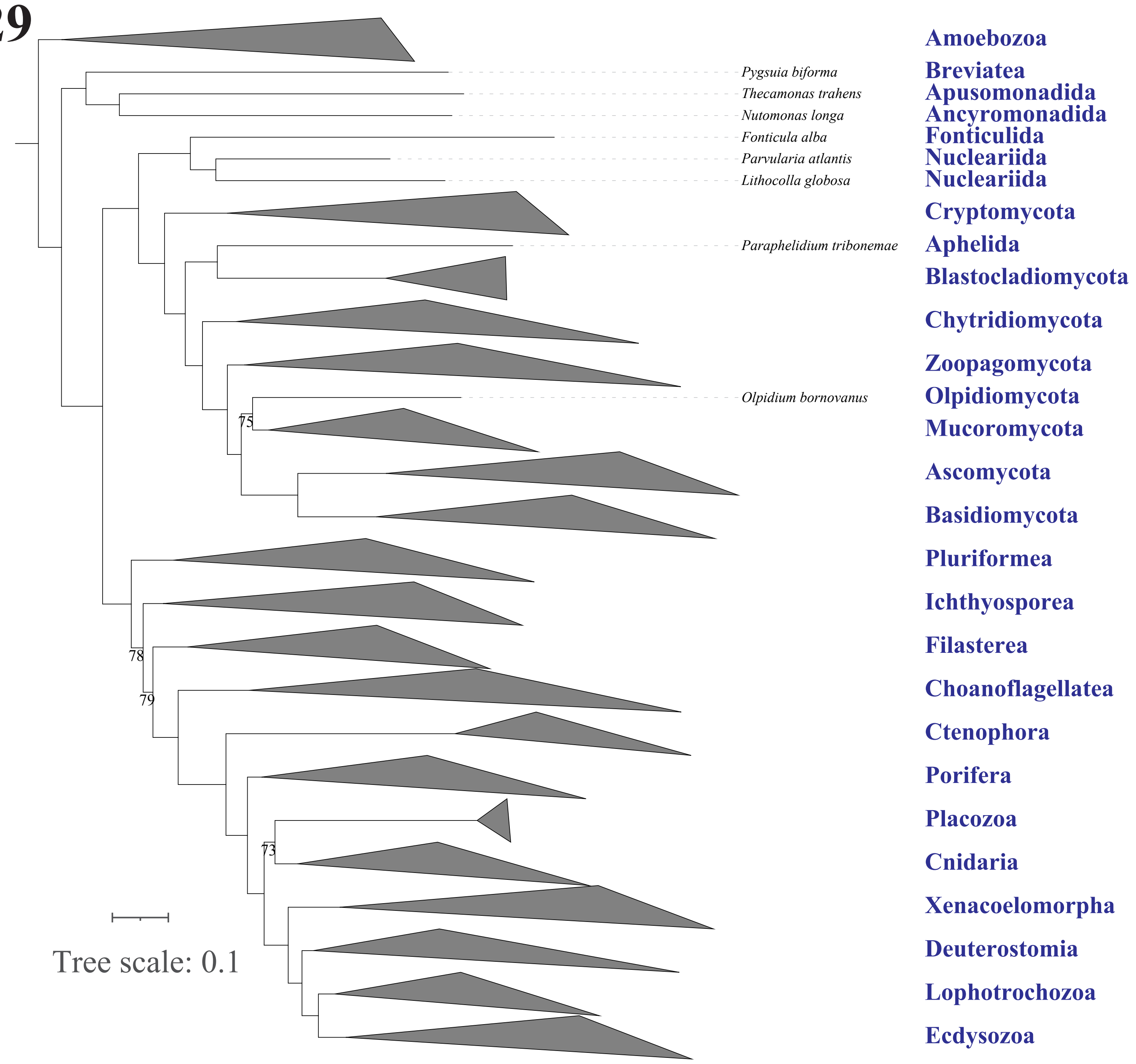

30

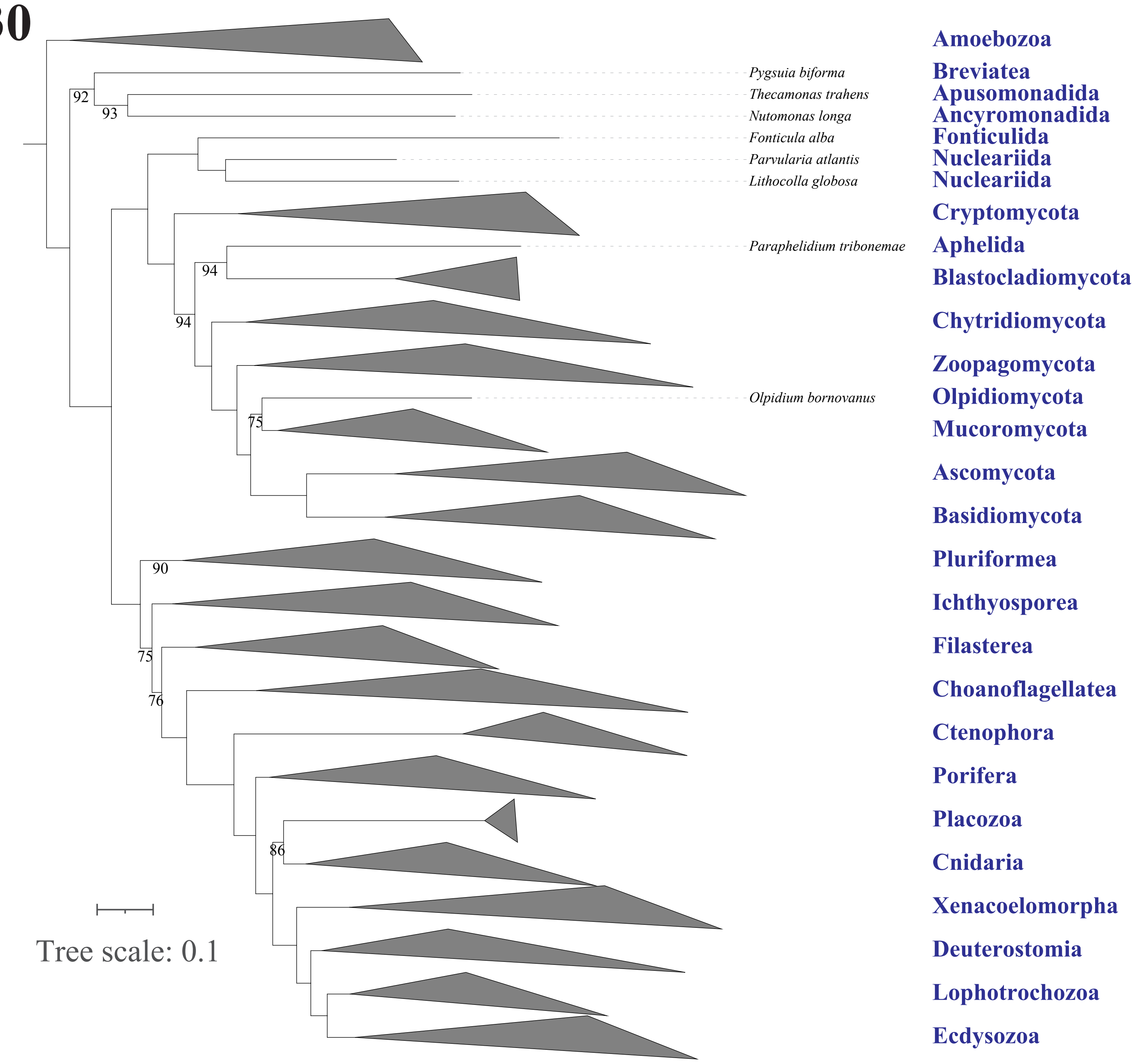

31

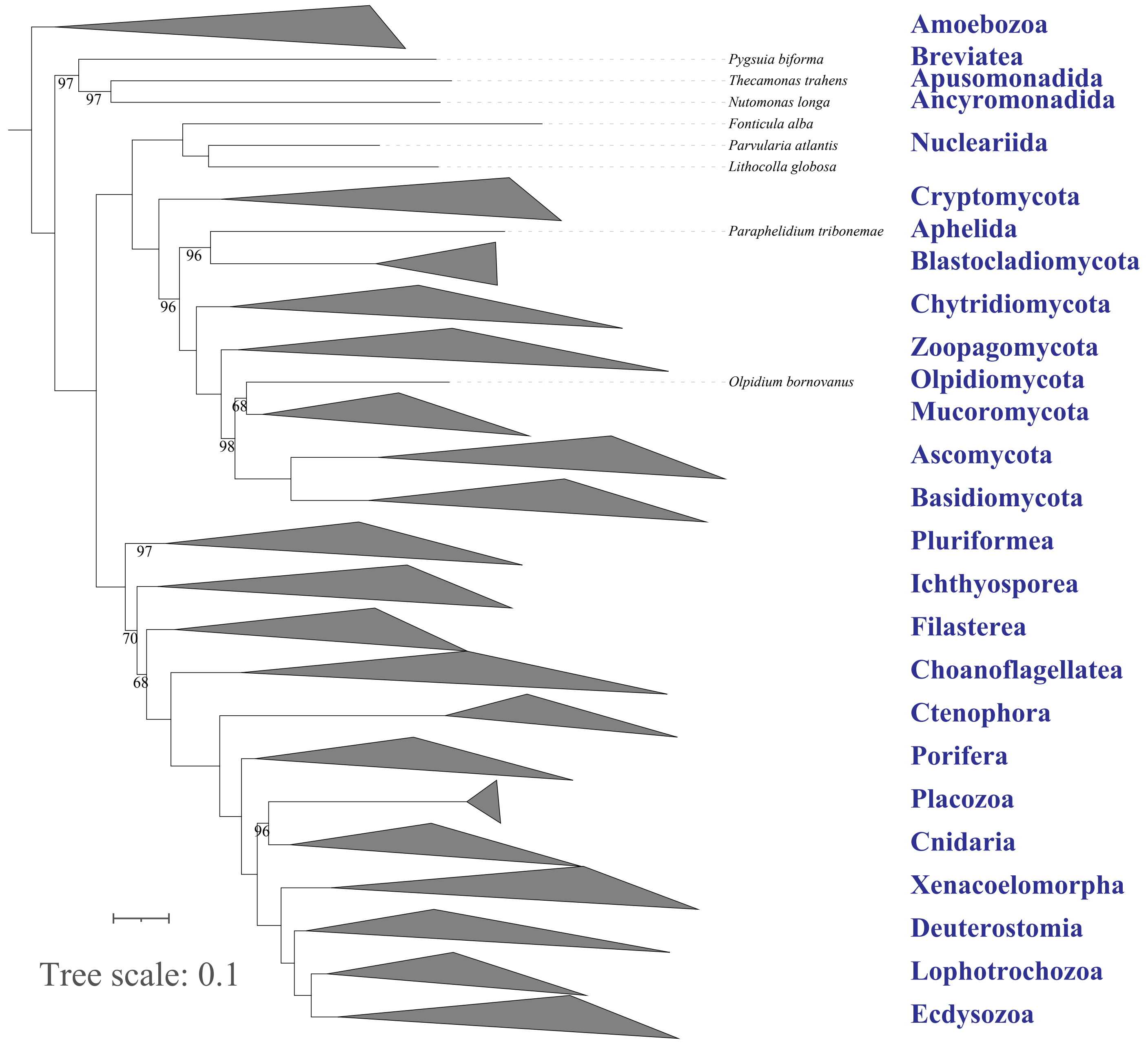

32

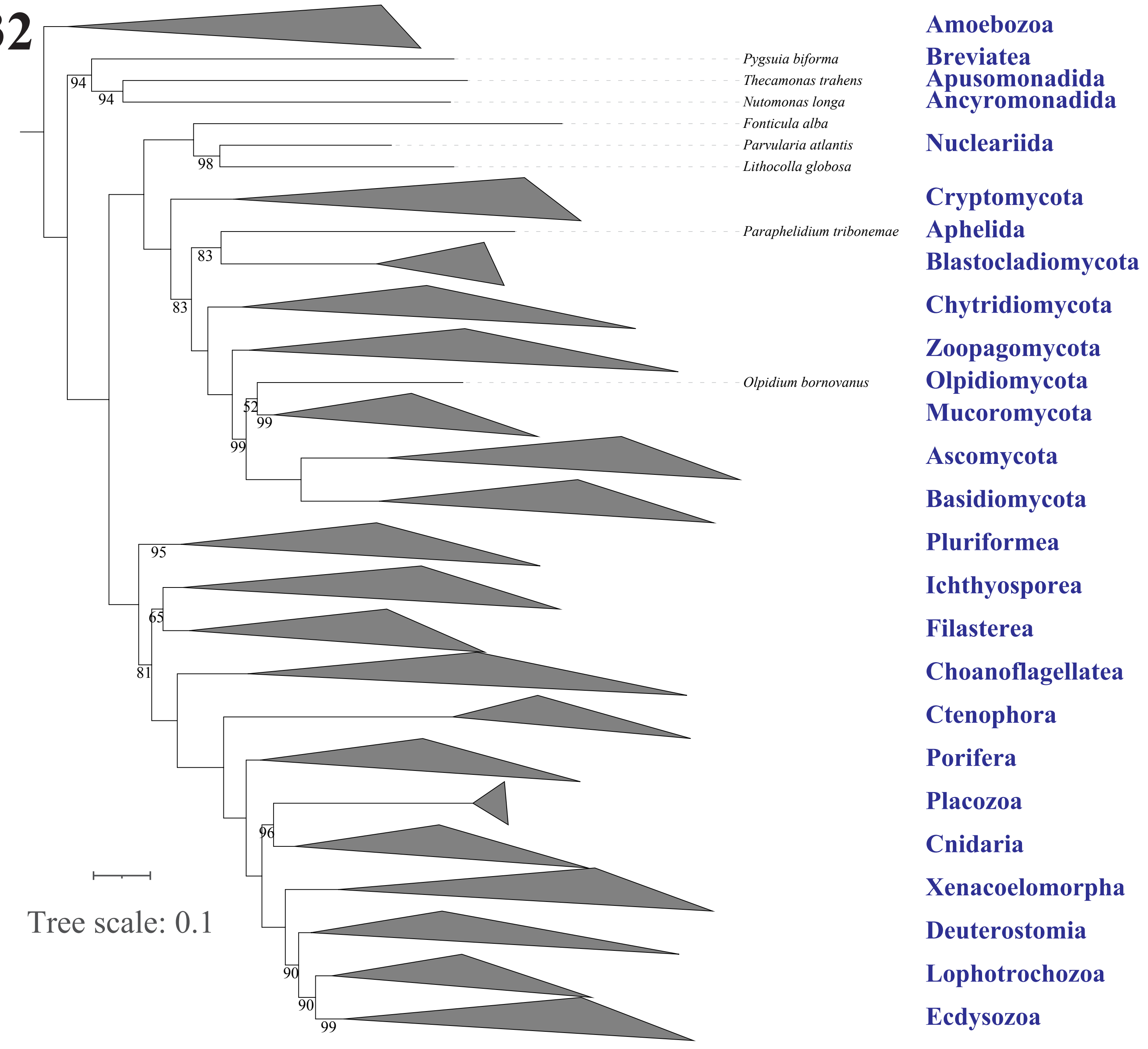

33

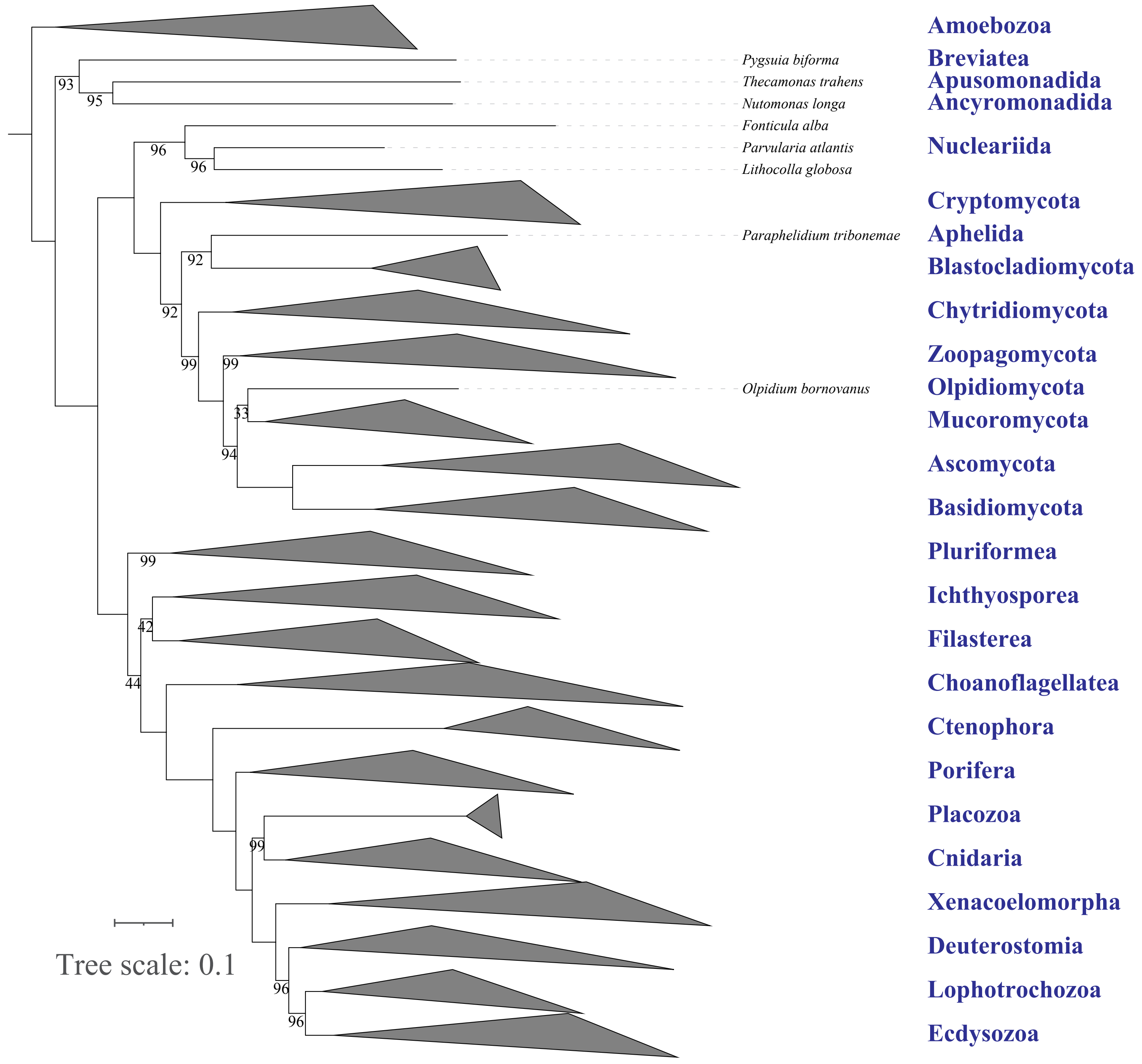

34

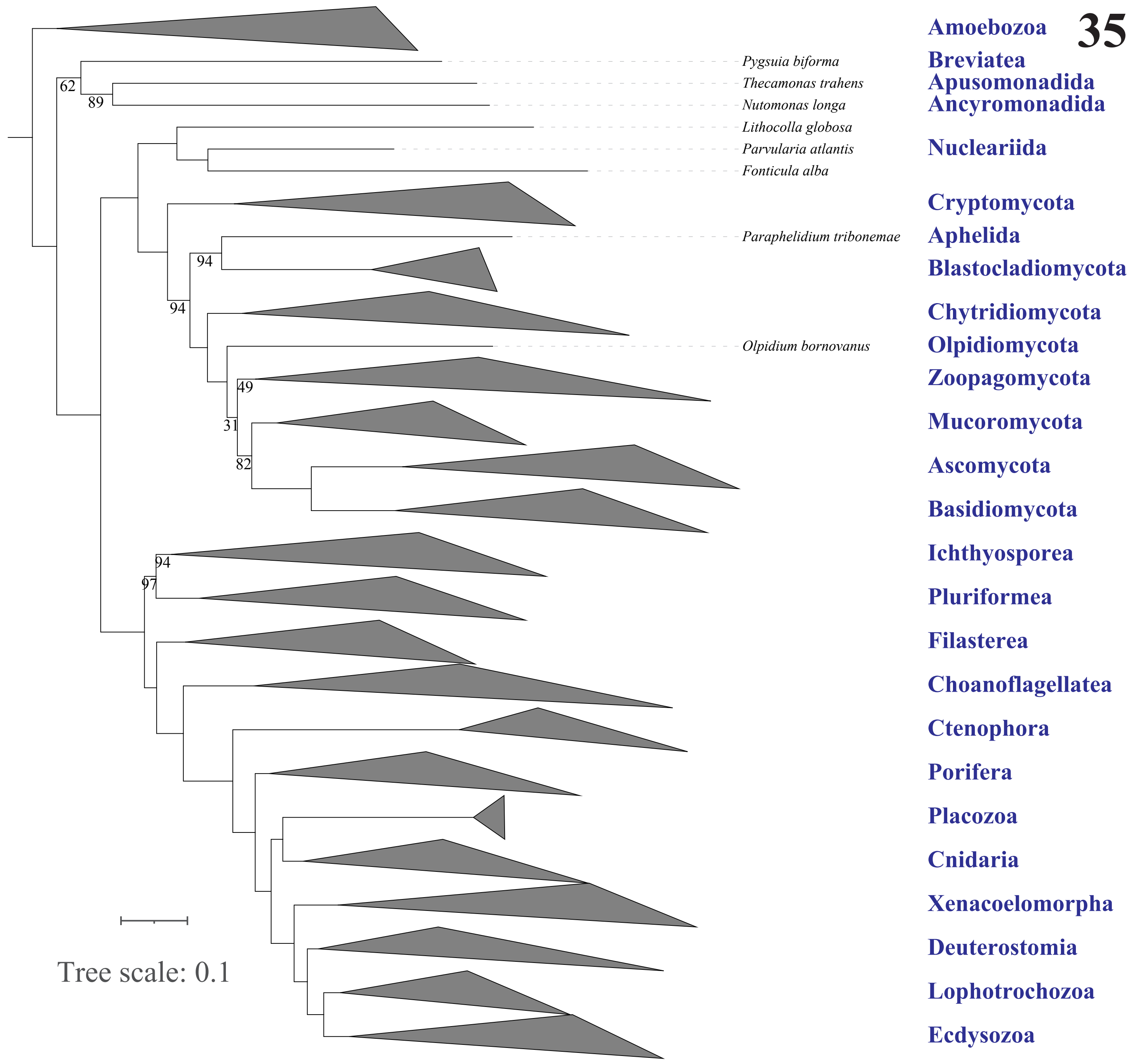

35

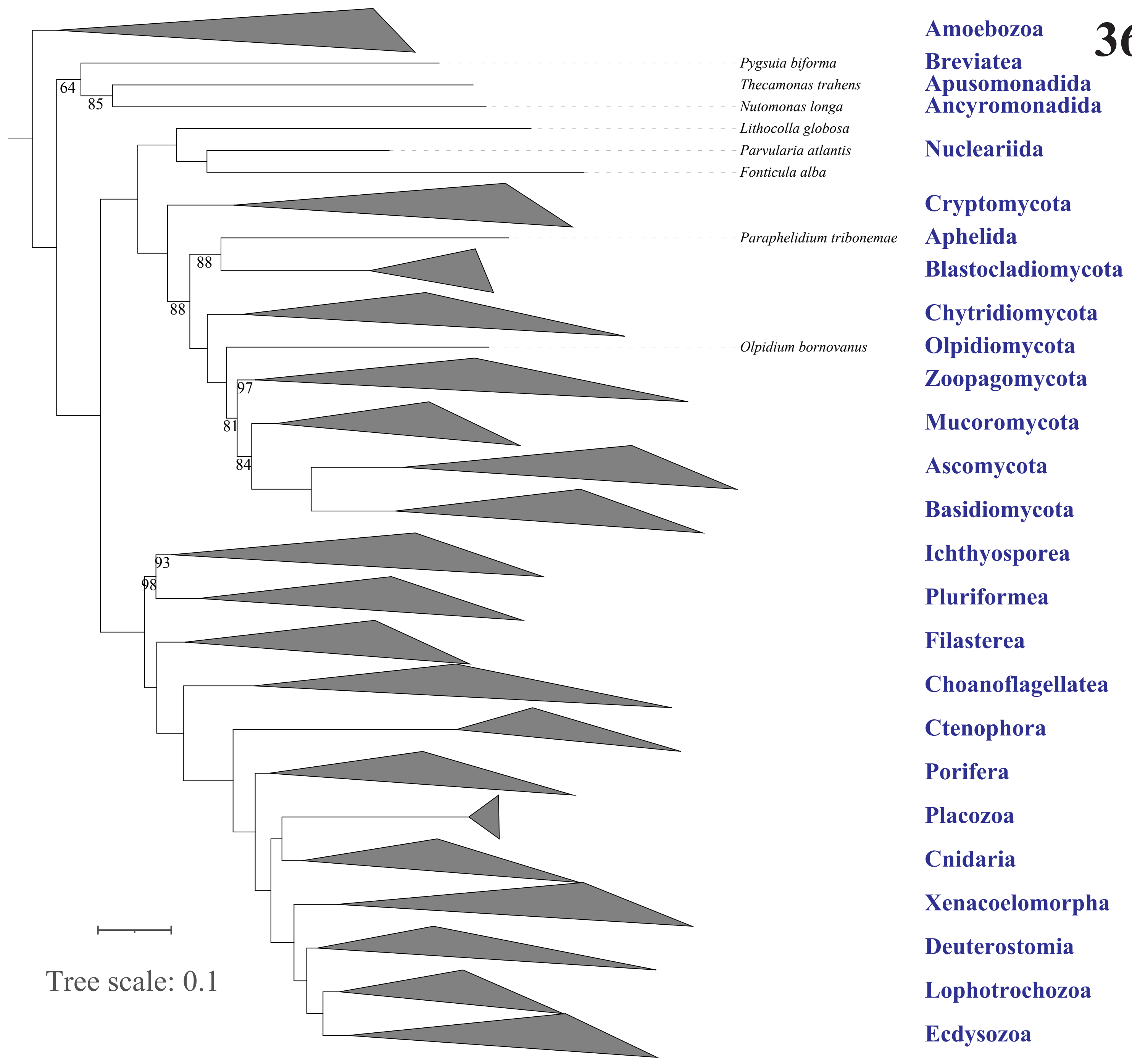

36

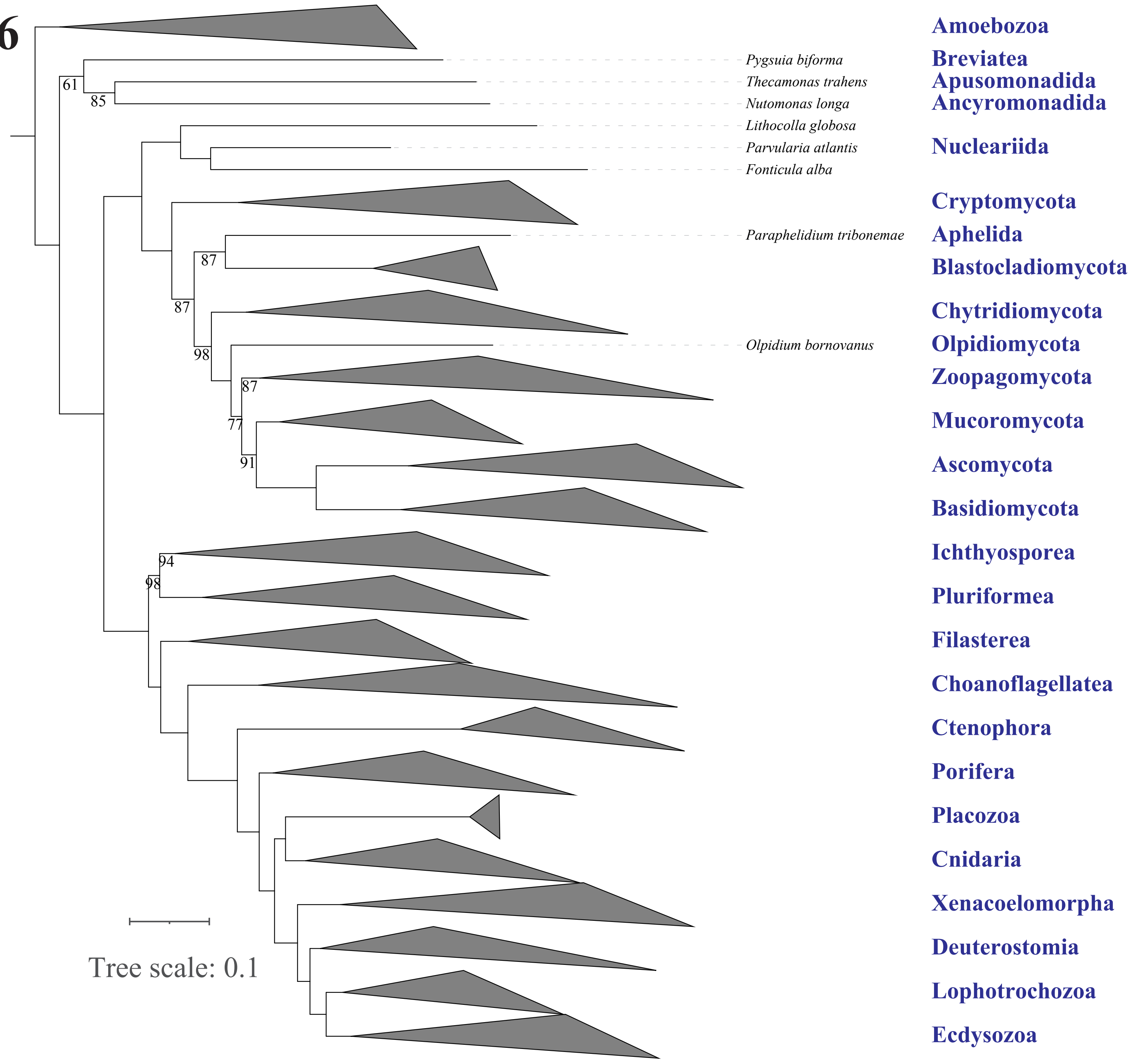

37

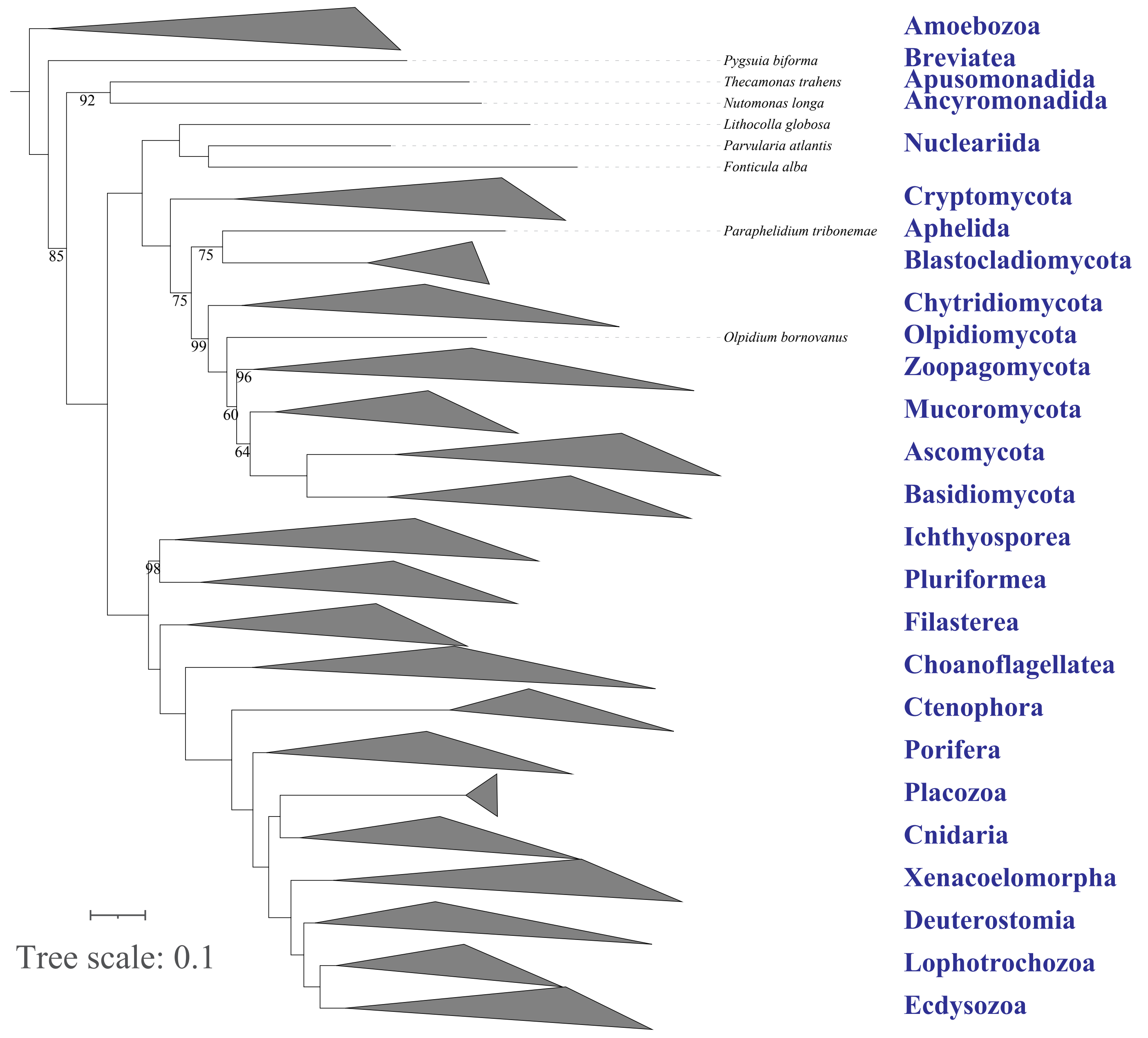

38

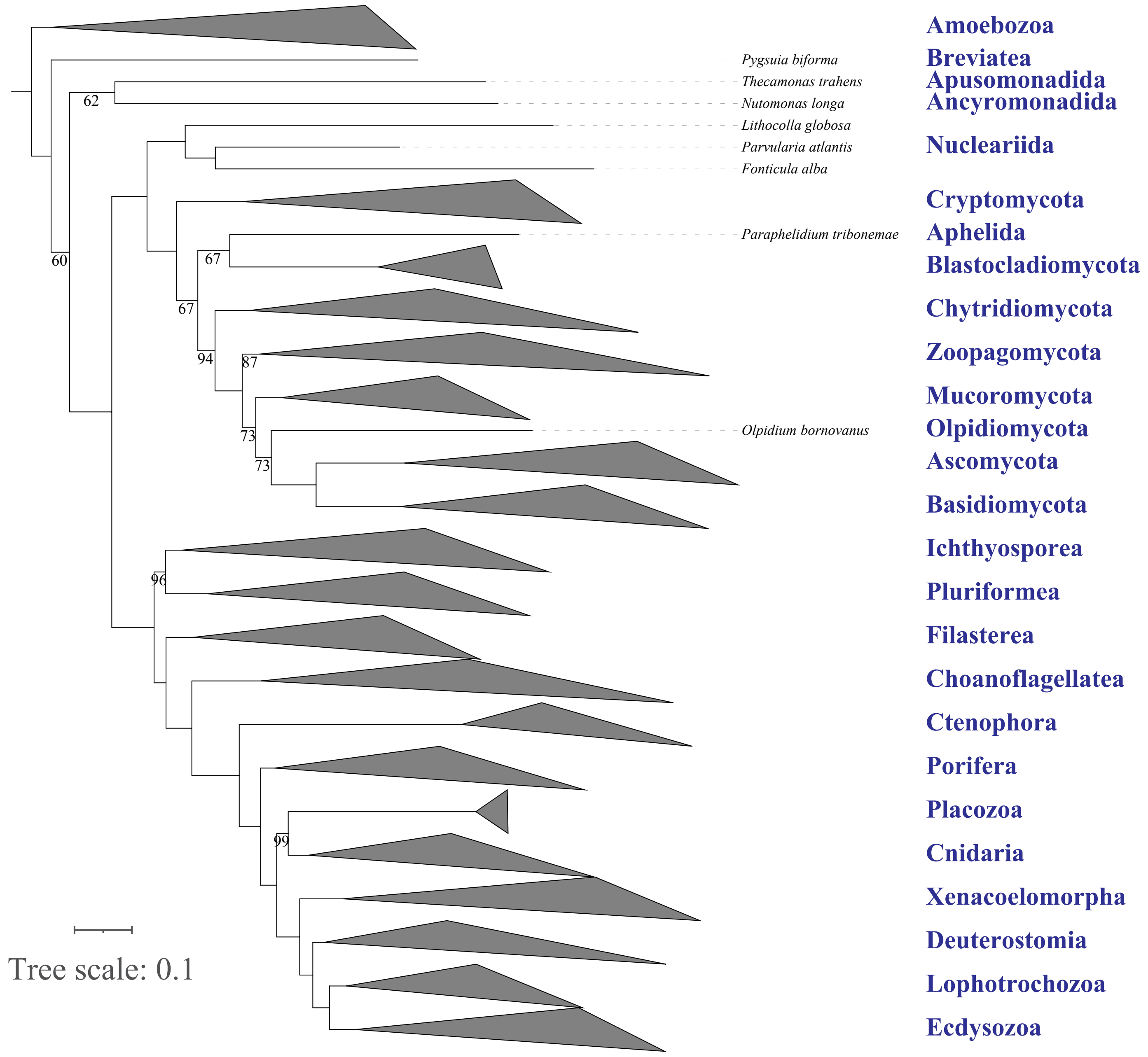

39

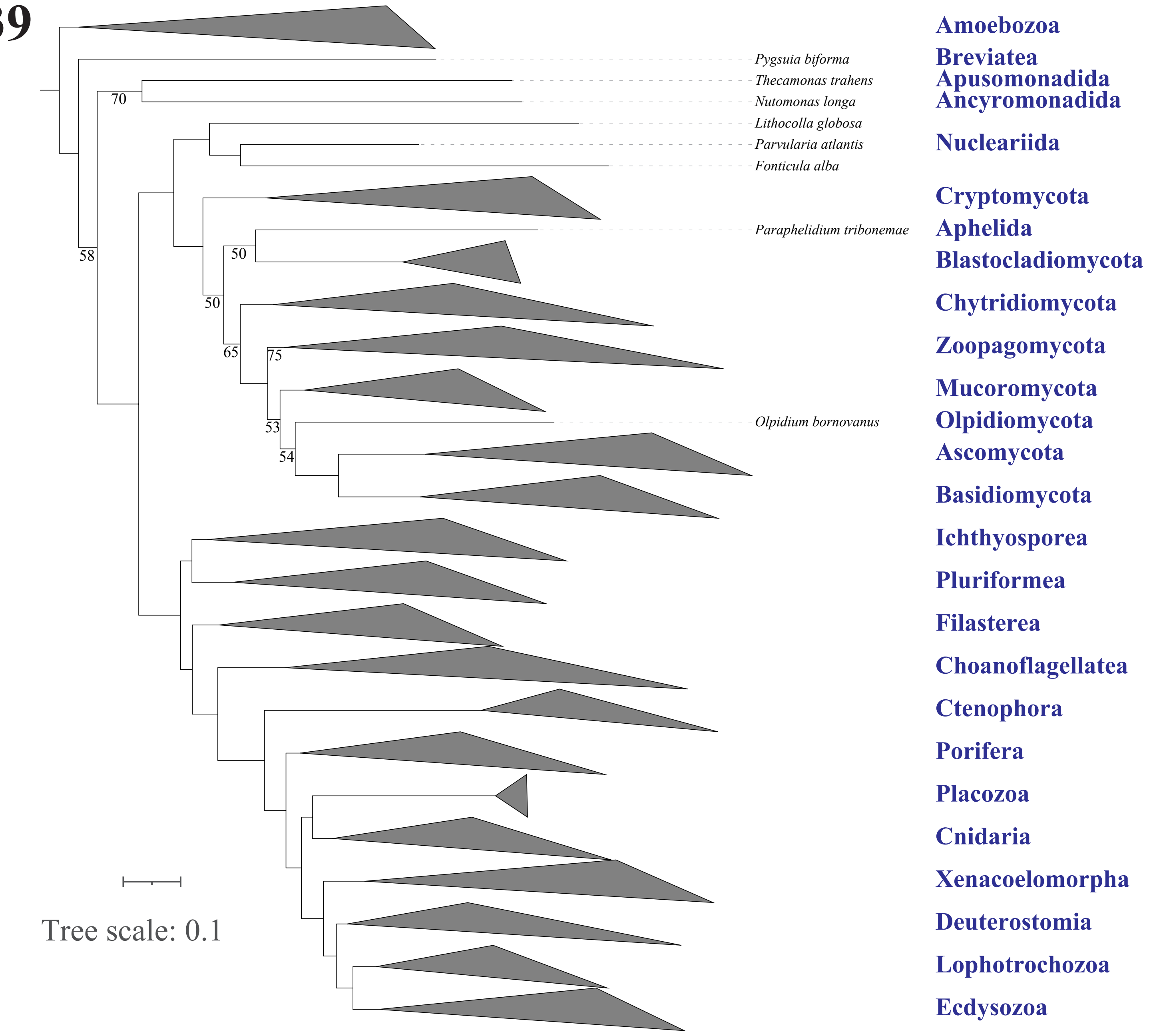

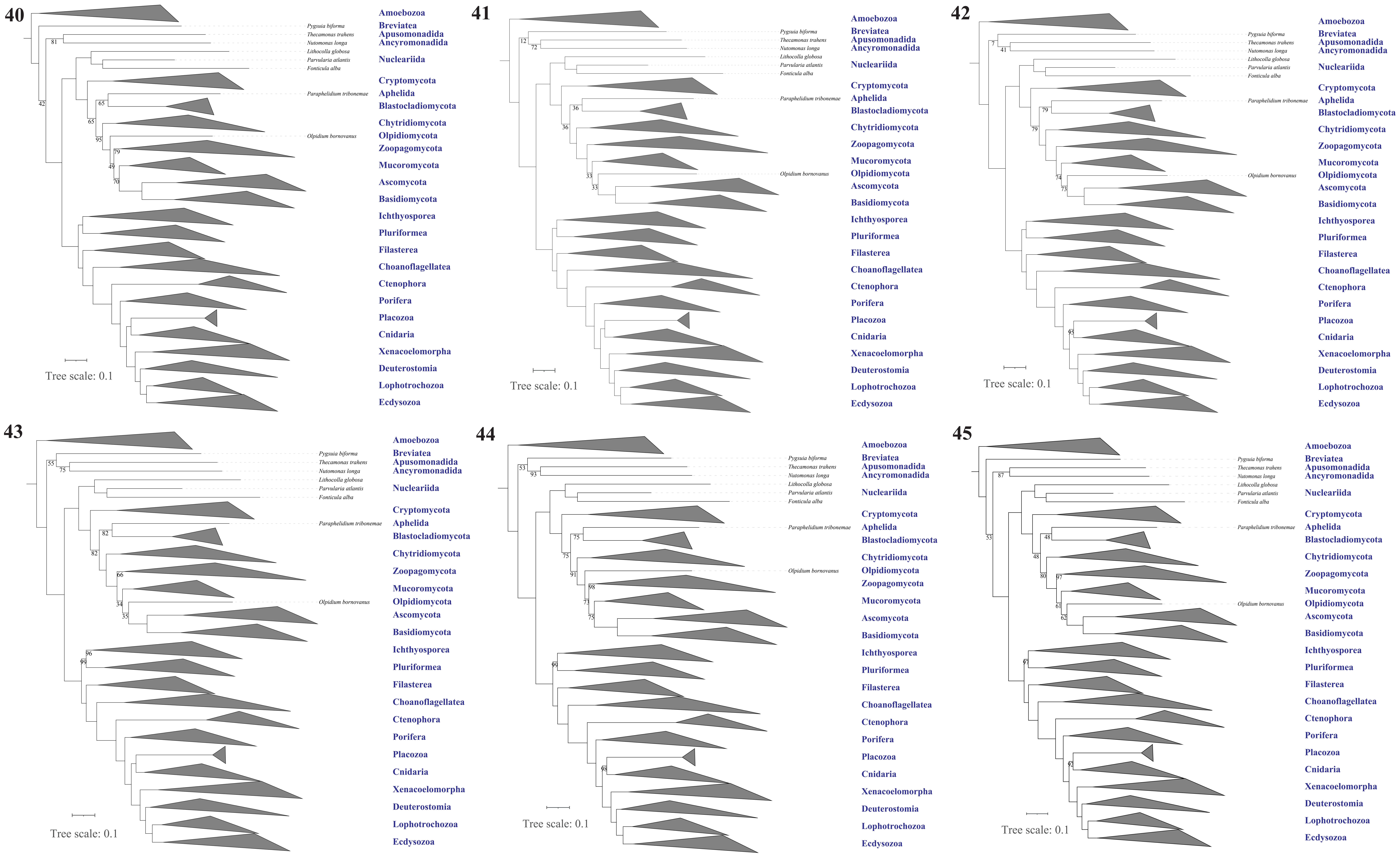
40
Pygsuia biforma
Thecamonas trahens
Nutomonas longa
Lithocolla globosa
Parvularia atlantis
Fonticula alba
Paraphelidium tribonemae
Olpidium bornovanus
Amoebozoa
Breviatea
Apusomonadida
Ancyromonadida
Nucleariida
Cryptomycota
Aphelida
Blastocladiomycota
Chytridiomycota
Olpidiomycota
Zoopagomycota
Mucoromycota
Ascomycota
Basidiomycota
Ichthyosporea
Pluriformea
Filasterea
Choanoflagellatea
Ctenophora
Porifera
Placozoa
Cnidaria
Xenacoelomorpha
Deuterostomia
Lophotrochozoa
Ecdysozoa
Tree scale: 0.1
41
Pygsuia biforma
Thecamonas trahens
Nutomonas longa
Lithocolla globosa
Parvularia atlantis
Fonticula alba
Paraphelidium tribonemae
Olpidium bornovanus
Amoebozoa
Breviatea
Apusomonadida
Ancyromonadida
Nucleariida
Cryptomycota
Aphelida
Blastocladiomycota
Chytridiomycota
Zoopagomycota
Mucoromycota
Olpidiomycota
Ascomycota
Basidiomycota
Ichthyosporea
Pluriformea
Filasterea
Choanoflagellatea
Ctenophora
Porifera
Placozoa
Cnidaria
Xenacoelomorpha
Deuterostomia
Lophotrochozoa
Ecdysozoa
Tree scale: 0.1
42
Pygsuia biforma
Thecamonas trahens
Nutomonas longa
Lithocolla globosa
Parvularia atlantis
Fonticula alba
Paraphelidium tribonemae
Olpidium bornovanus
Amoebozoa
Breviatea
Apusomonadida
Ancyromonadida
Nucleariida
Cryptomycota
Aphelida
Blastocladiomycota
Chytridiomycota
Zoopagomycota
Mucoromycota
Olpidiomycota
Ascomycota
Basidiomycota
Ichthyosporea
Pluriformea
Filasterea
Choanoflagellatea
Ctenophora
Porifera
Placozoa
Cnidaria
Xenacoelomorpha
Deuterostomia
Lophotrochozoa
Ecdysozoa
Tree scale: 0.1
43
Pygsuia biforma
Thecamonas trahens
Nutomonas longa
Lithocolla globosa
Parvularia atlantis
Fonticula alba
Paraphelidium tribonemae
Olpidium bornovanus
Amoebozoa
Breviatea
Apusomonadida
Ancyromonadida
Nucleariida
Cryptomycota
Aphelida
Blastocladiomycota
Chytridiomycota
Zoopagomycota
Mucoromycota
Olpidiomycota
Ascomycota
Basidiomycota
Ichthyosporea
Pluriformea
Filasterea
Choanoflagellatea
Ctenophora
Porifera
Placozoa
Cnidaria
Xenacoelomorpha
Deuterostomia
Lophotrochozoa
Ecdysozoa
Tree scale: 0.1
44
Pygsuia biforma
Thecamonas trahens
Nutomonas longa
Lithocolla globosa
Parvularia atlantis
Fonticula alba
Paraphelidium tribonemae
Olpidium bornovanus
Amoebozoa
Breviatea
Apusomonadida
Ancyromonadida
Nucleariida
Cryptomycota
Aphelida
Blastocladiomycota
Chytridiomycota
Olpidiomycota
Zoopagomycota
Mucoromycota
Ascomycota
Basidiomycota
Ichthyosporea
Pluriformea
Filasterea
Choanoflagellatea
Ctenophora
Porifera
Placozoa
Cnidaria
Xenacoelomorpha
Deuterostomia
Lophotrochozoa
Ecdysozoa
Tree scale: 0.1
45
Pygsuia biforma
Thecamonas trahens
Nutomonas longa
Lithocolla globosa
Parvularia atlantis
Fonticula alba
Paraphelidium tribonemae
Olpidium bornovanus
Amoebozoa
Breviatea
Apusomonadida
Ancyromonadida
Nucleariida
Cryptomycota
Aphelida
Blastocladiomycota
Chytridiomycota
Zoopagomycota
Mucoromycota
Olpidiomycota
Ascomycota
Basidiomycota
Ichthyosporea
Pluriformea
Filasterea
Choanoflagellatea
Ctenophora
Porifera
Placozoa
Cnidaria
Xenacoelomorpha
Deuterostomia
Lophotrochozoa
Ecdysozoa
Tree scale: 0.1

46

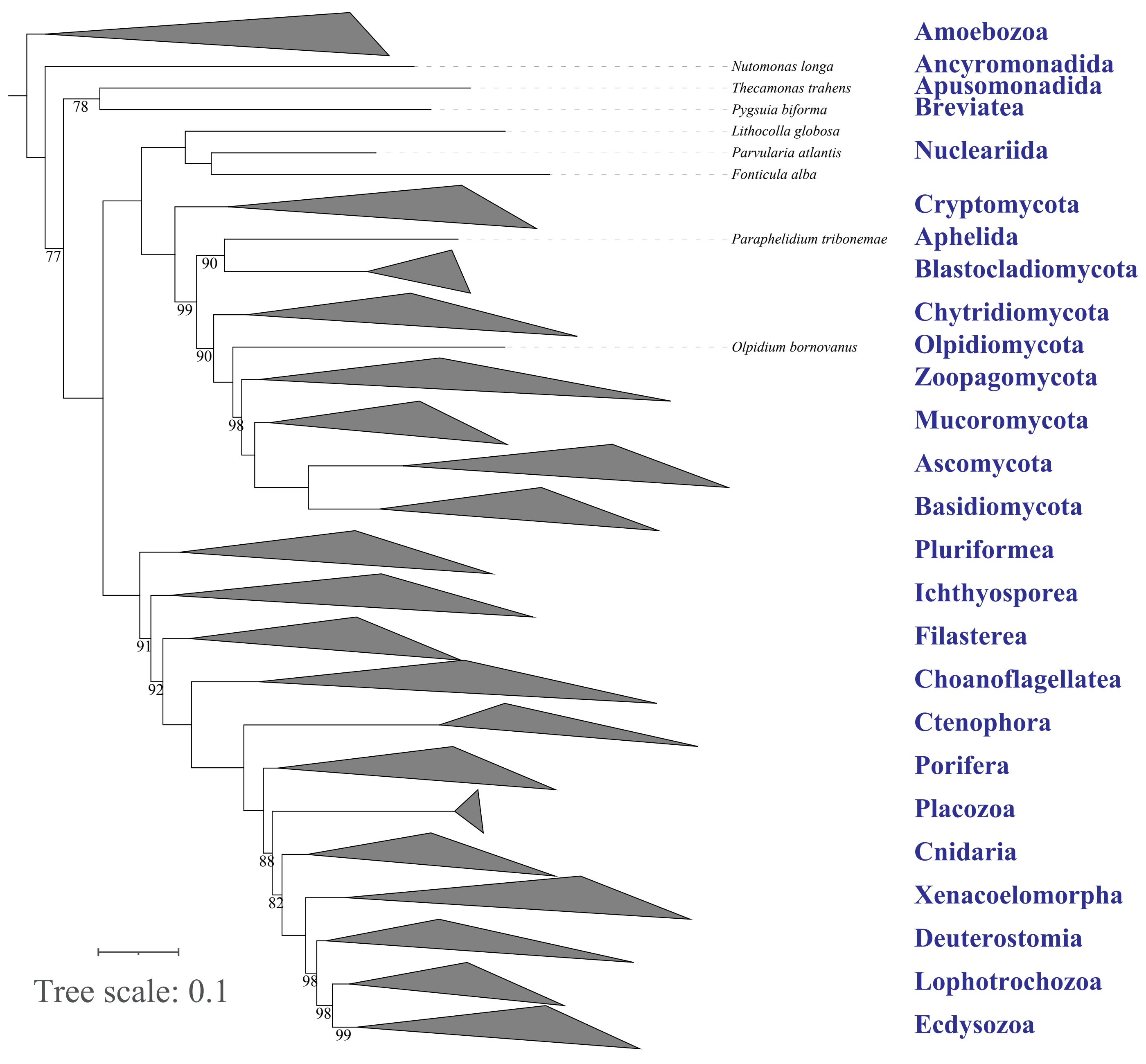

47

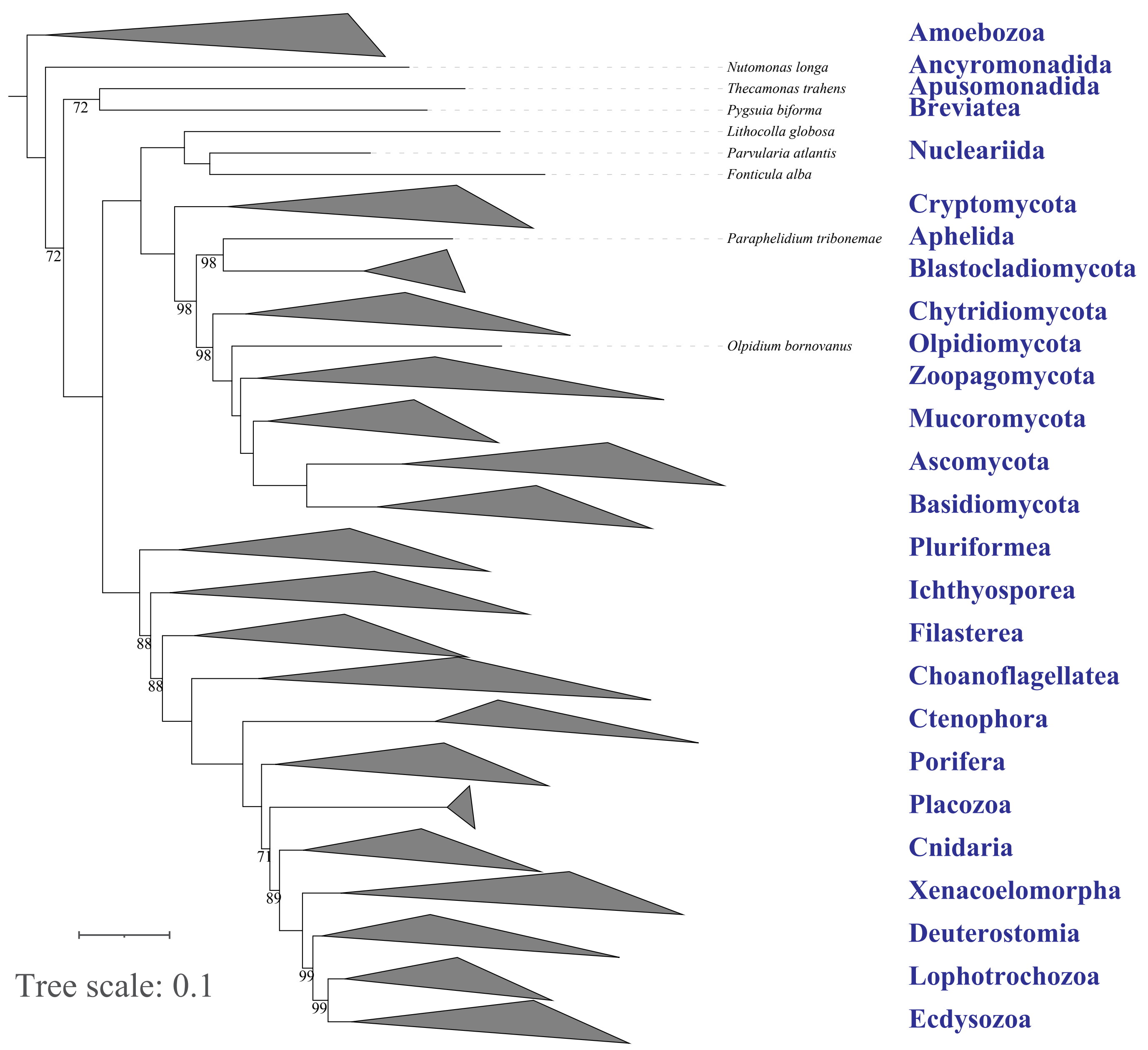

48

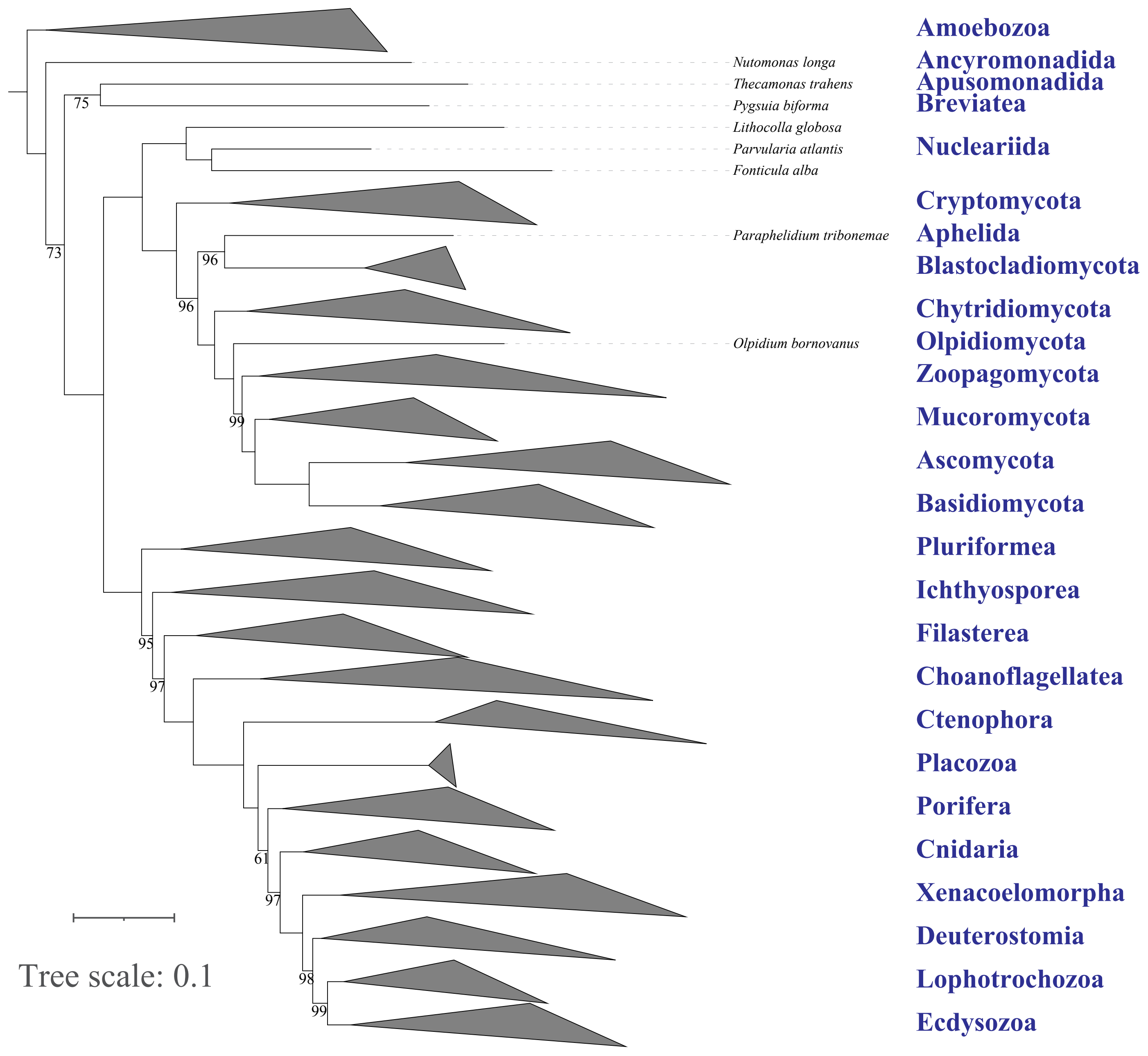

49

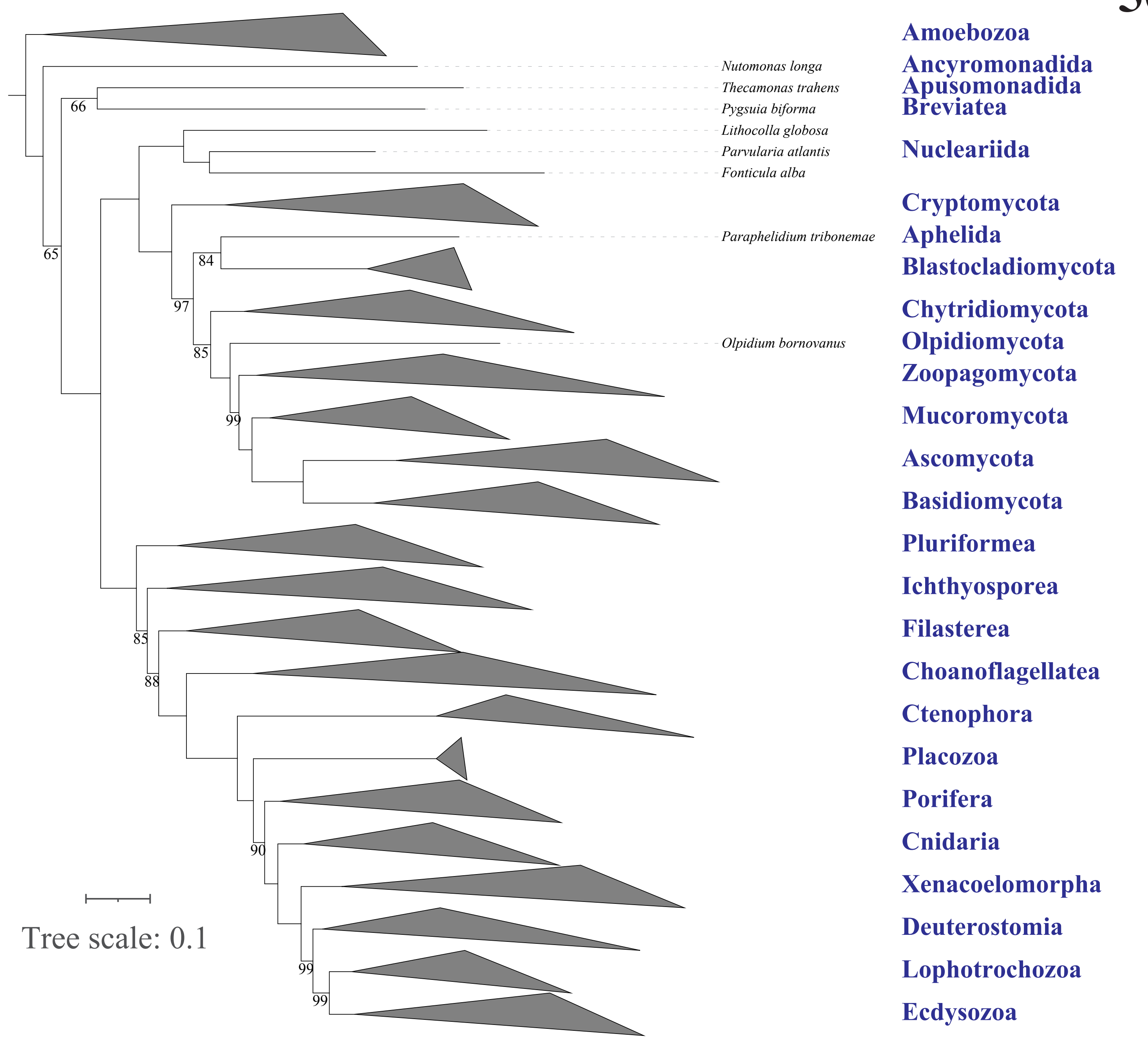

50

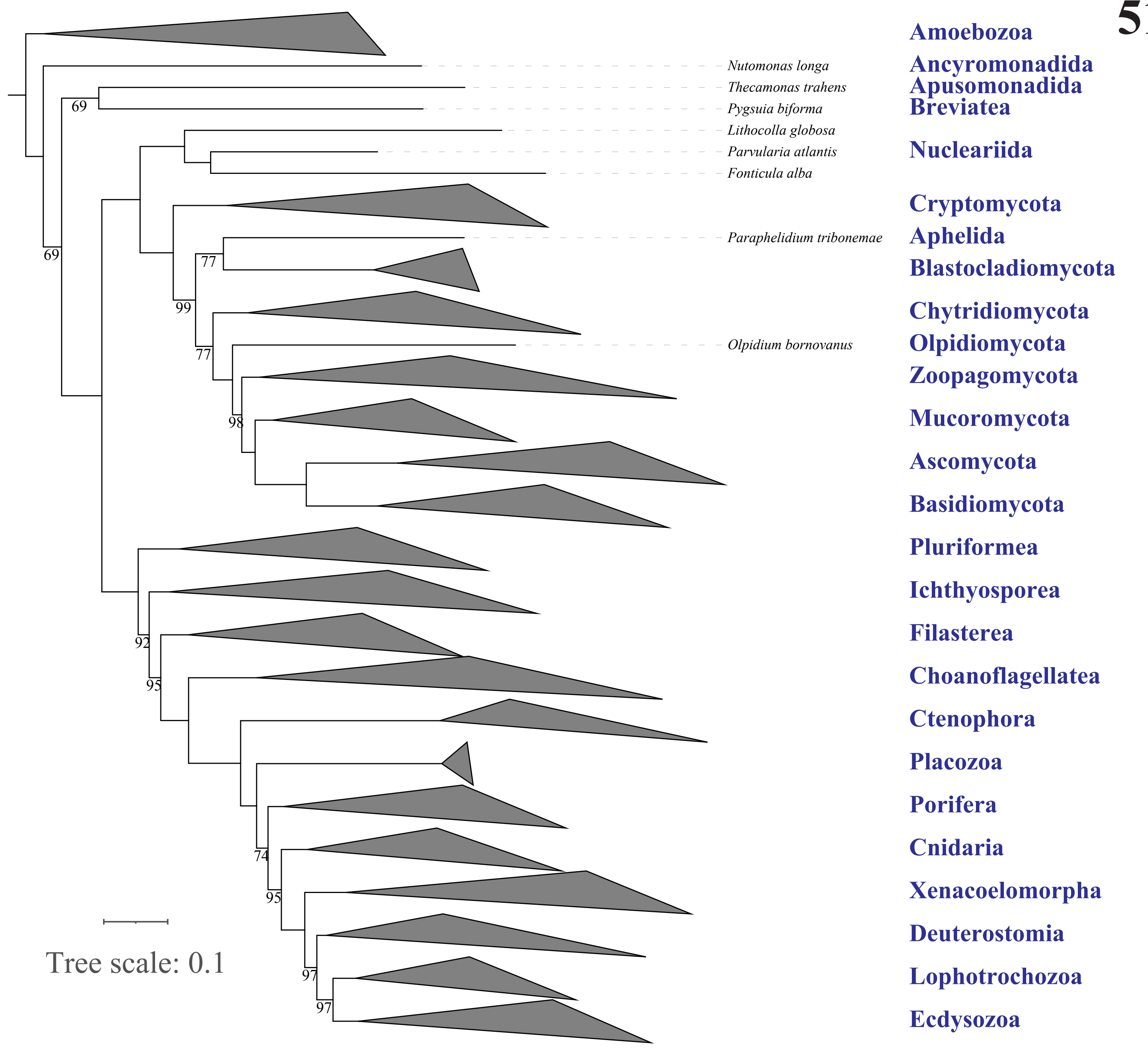

51

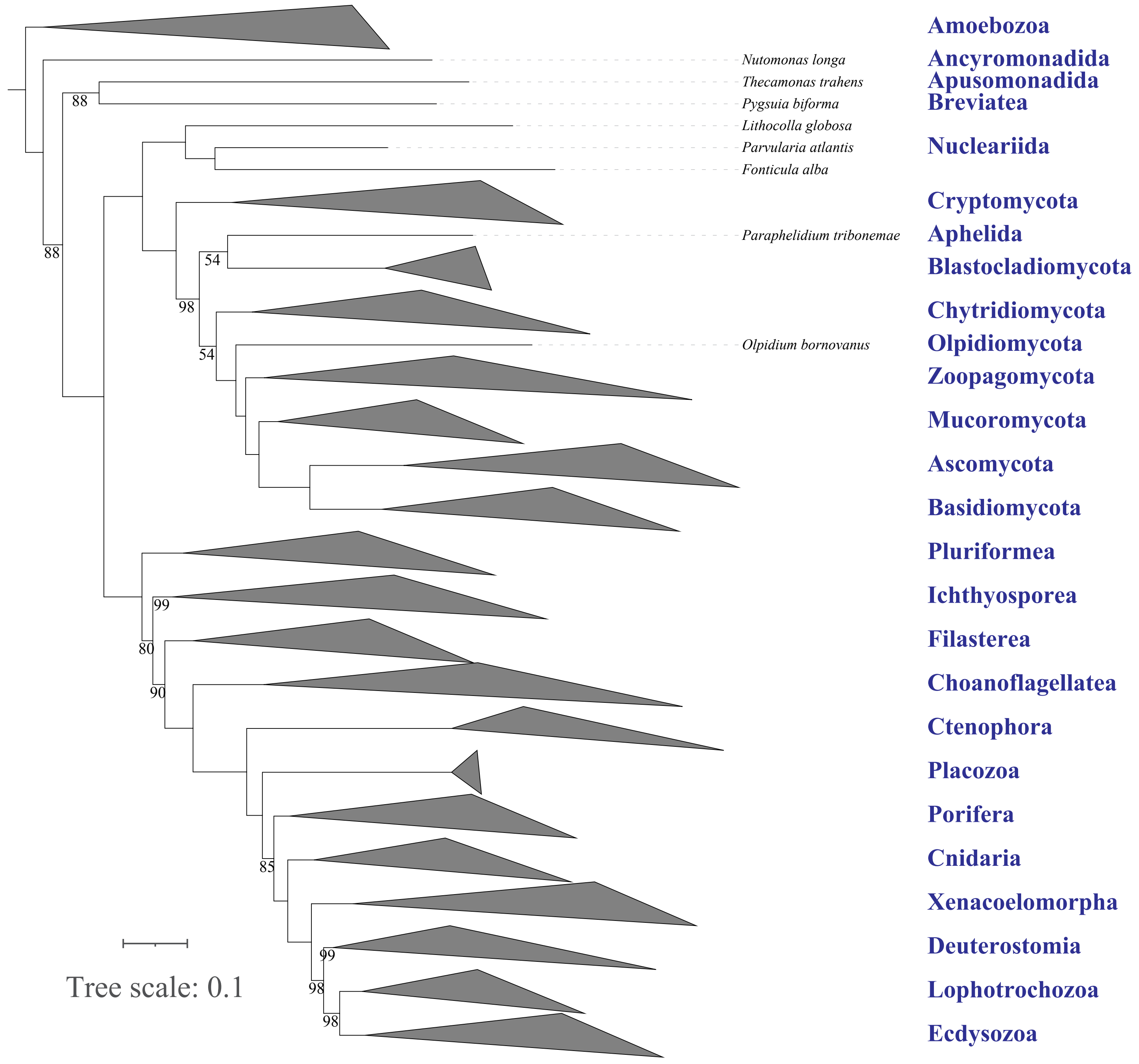

52

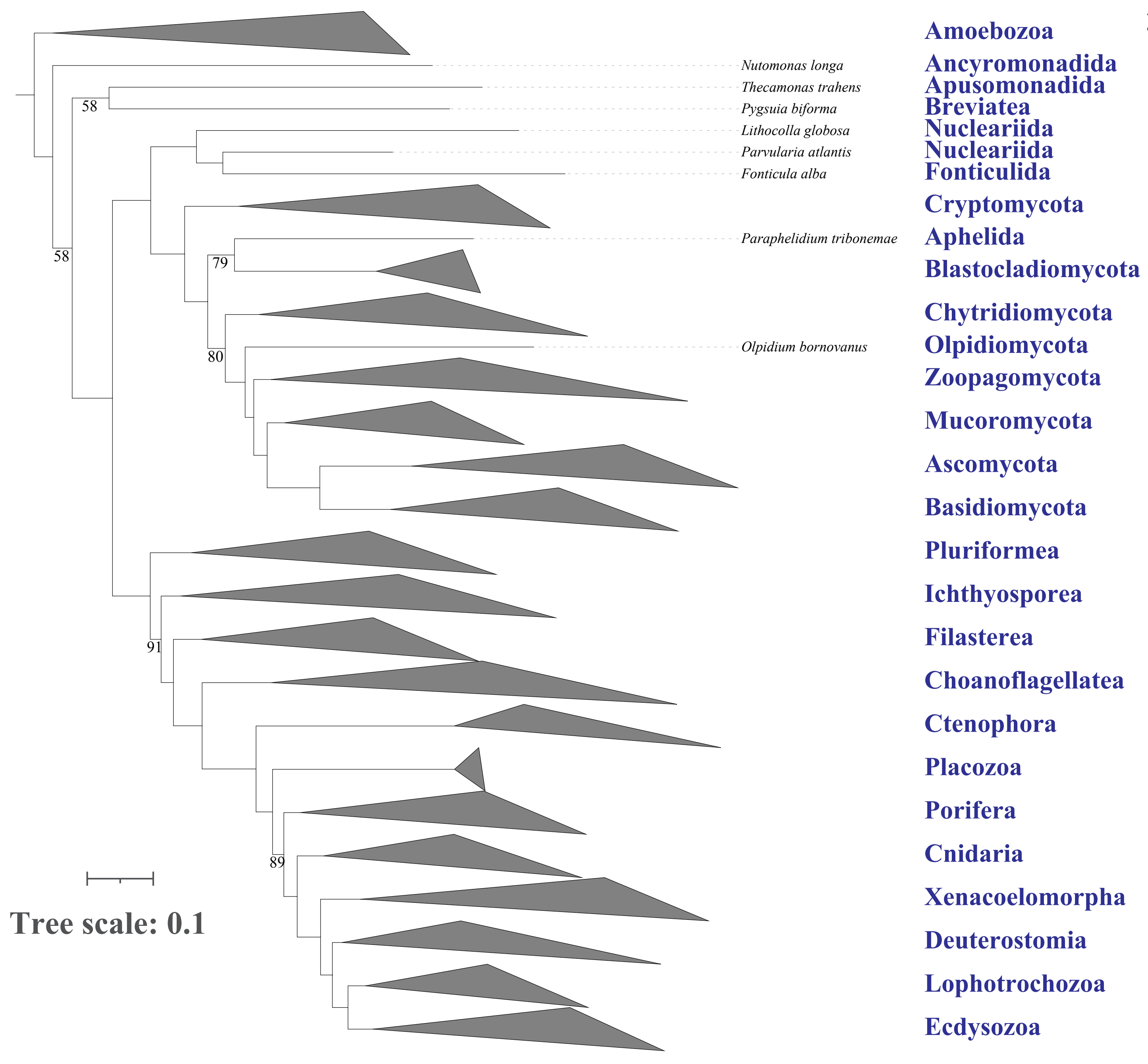

53

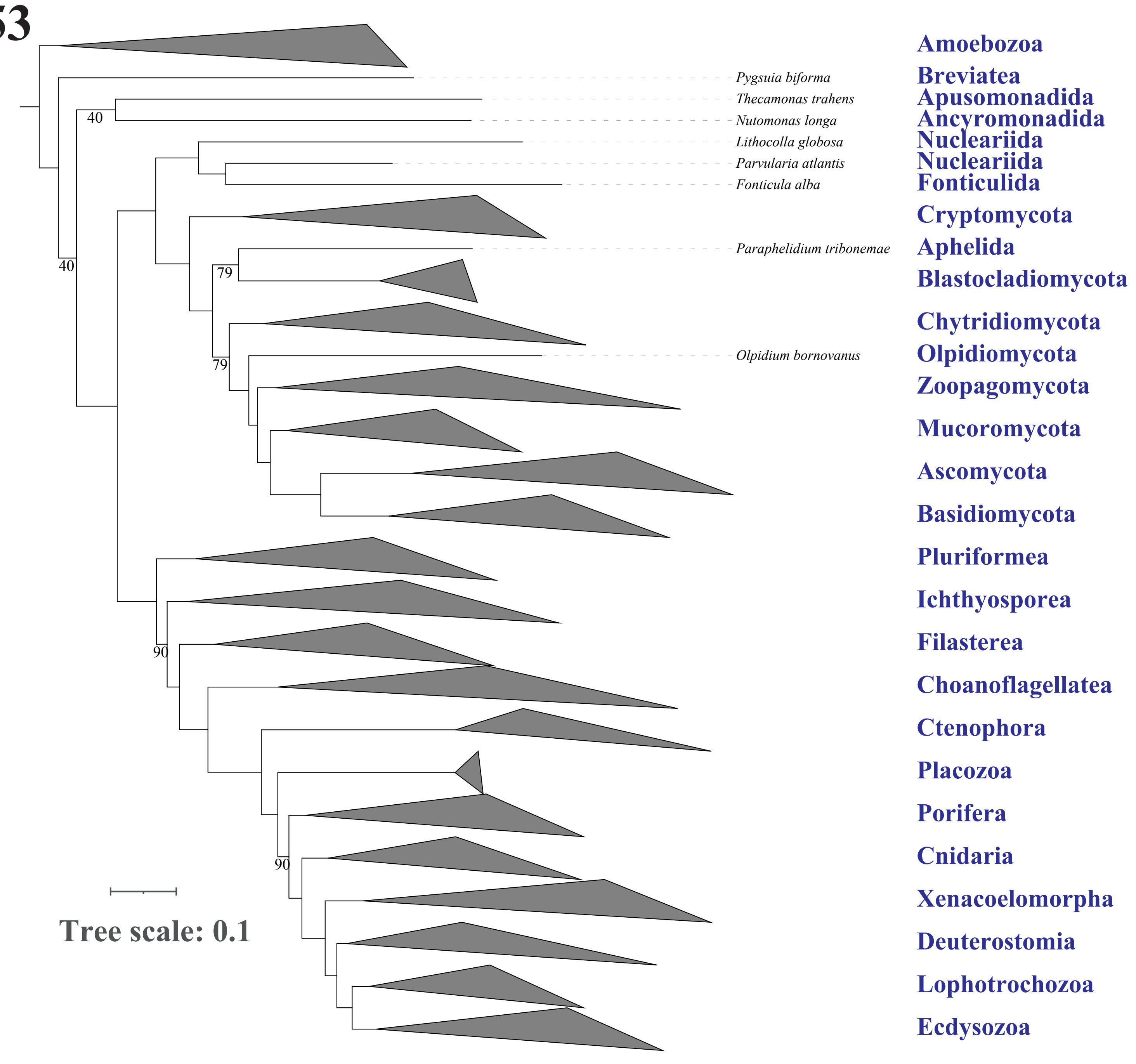

54

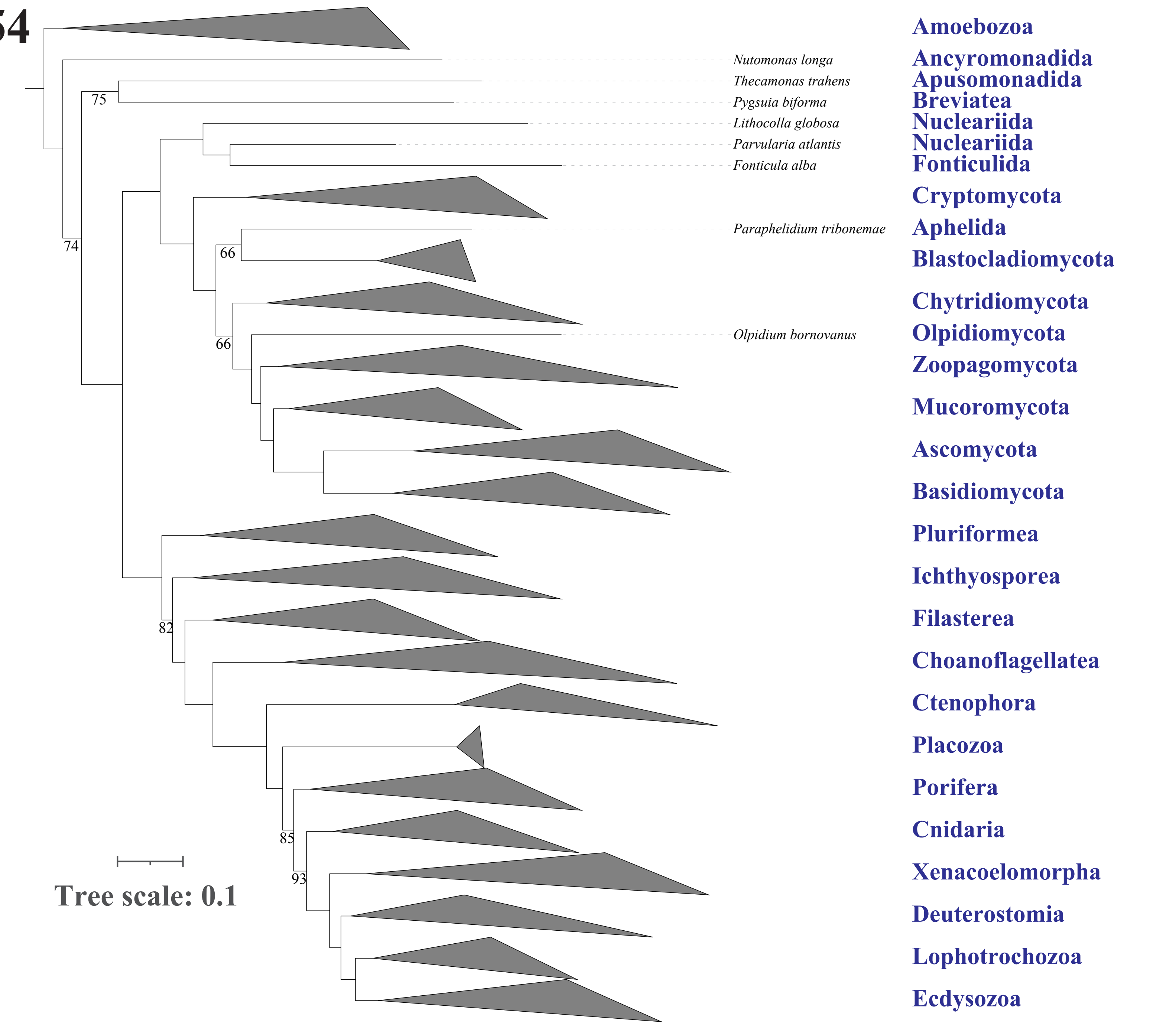

55

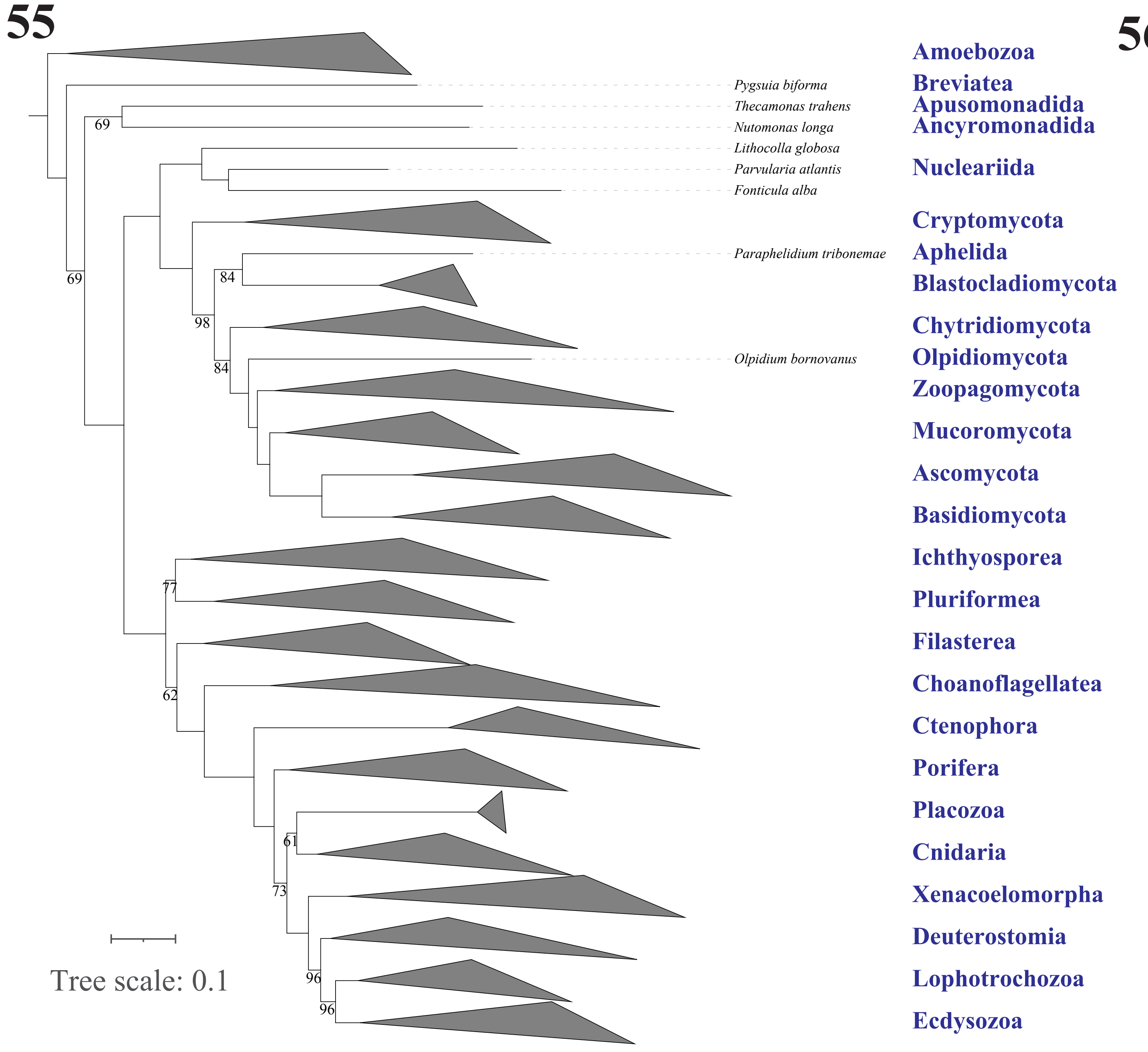

56

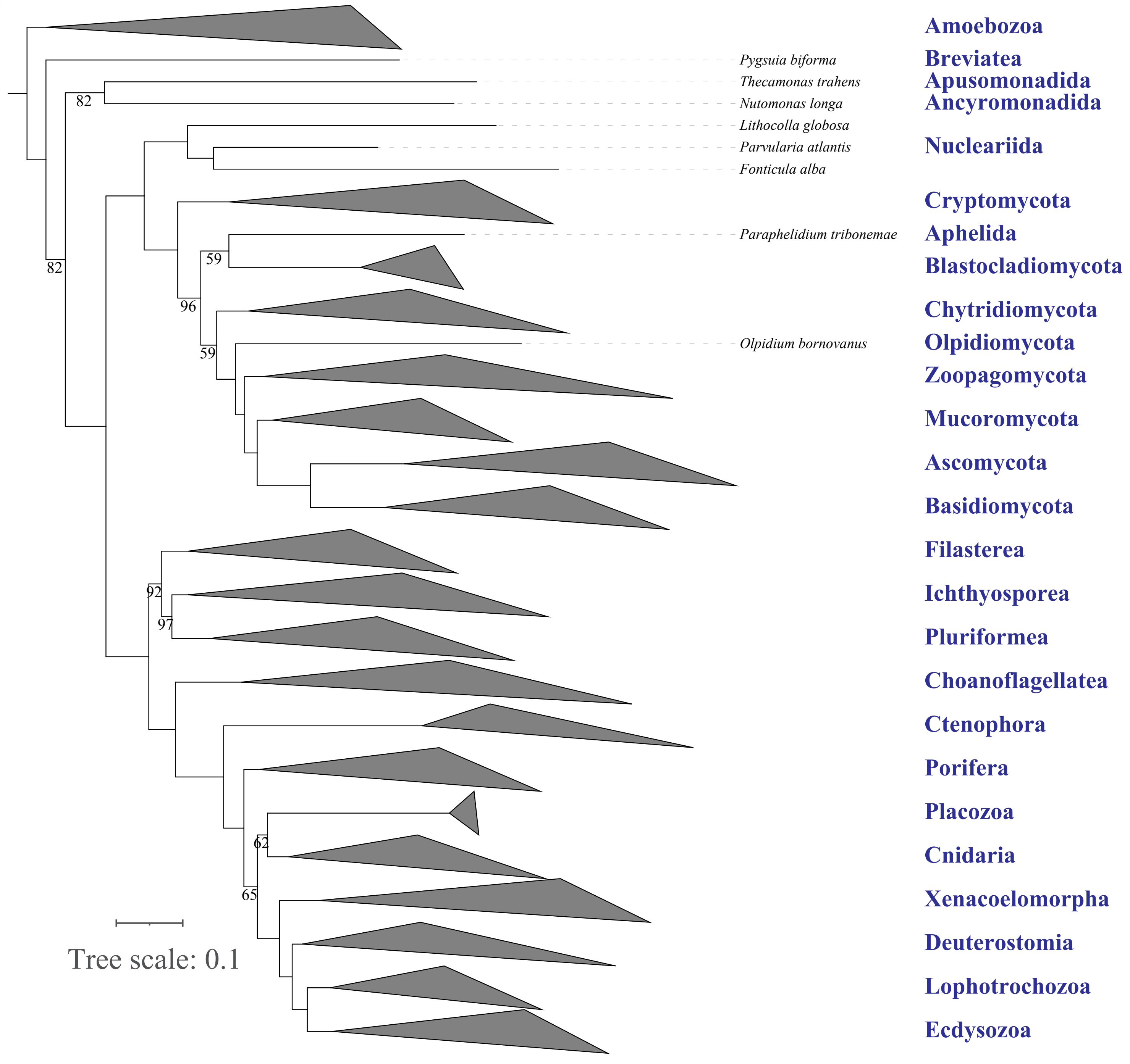

57

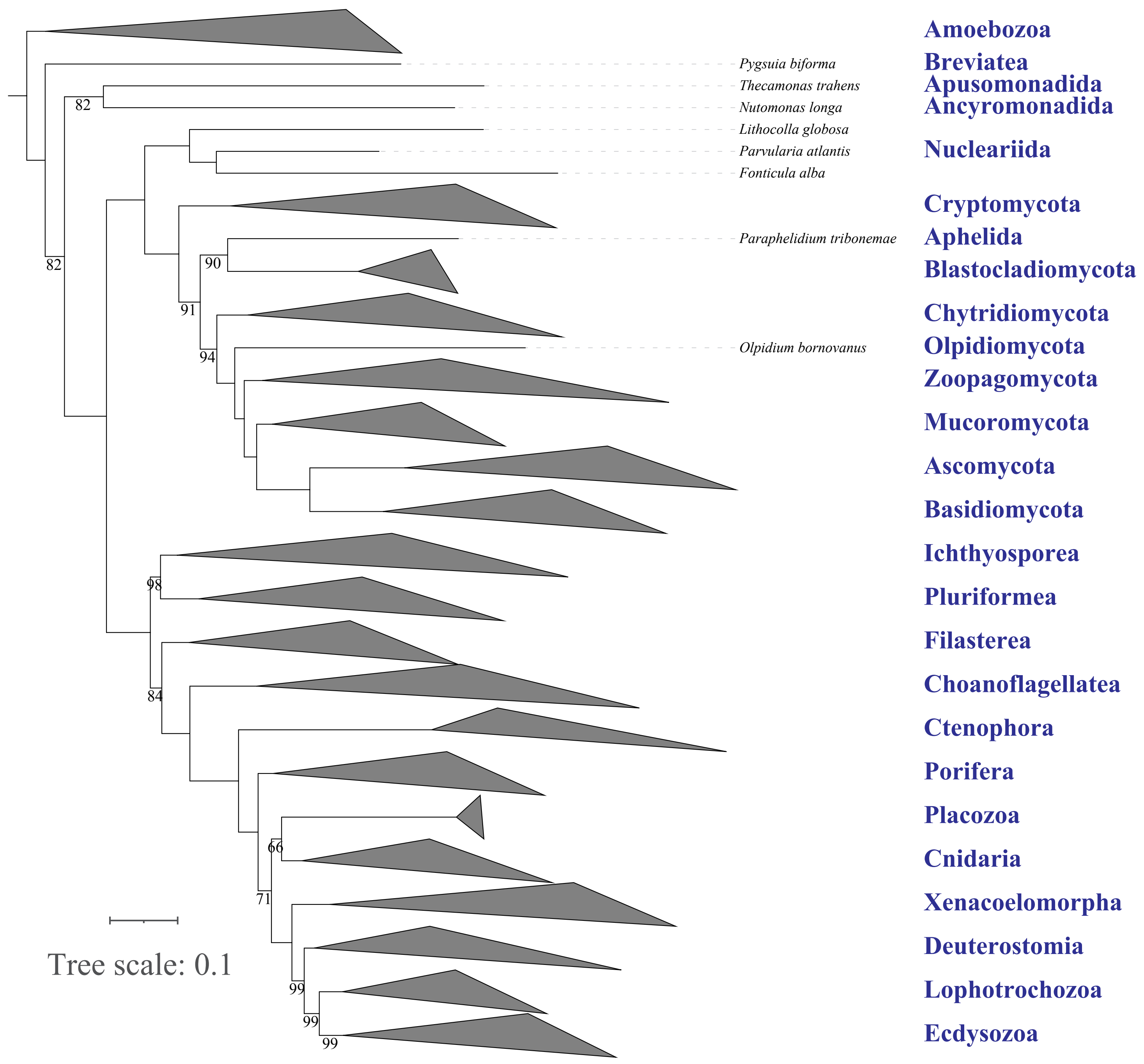

58

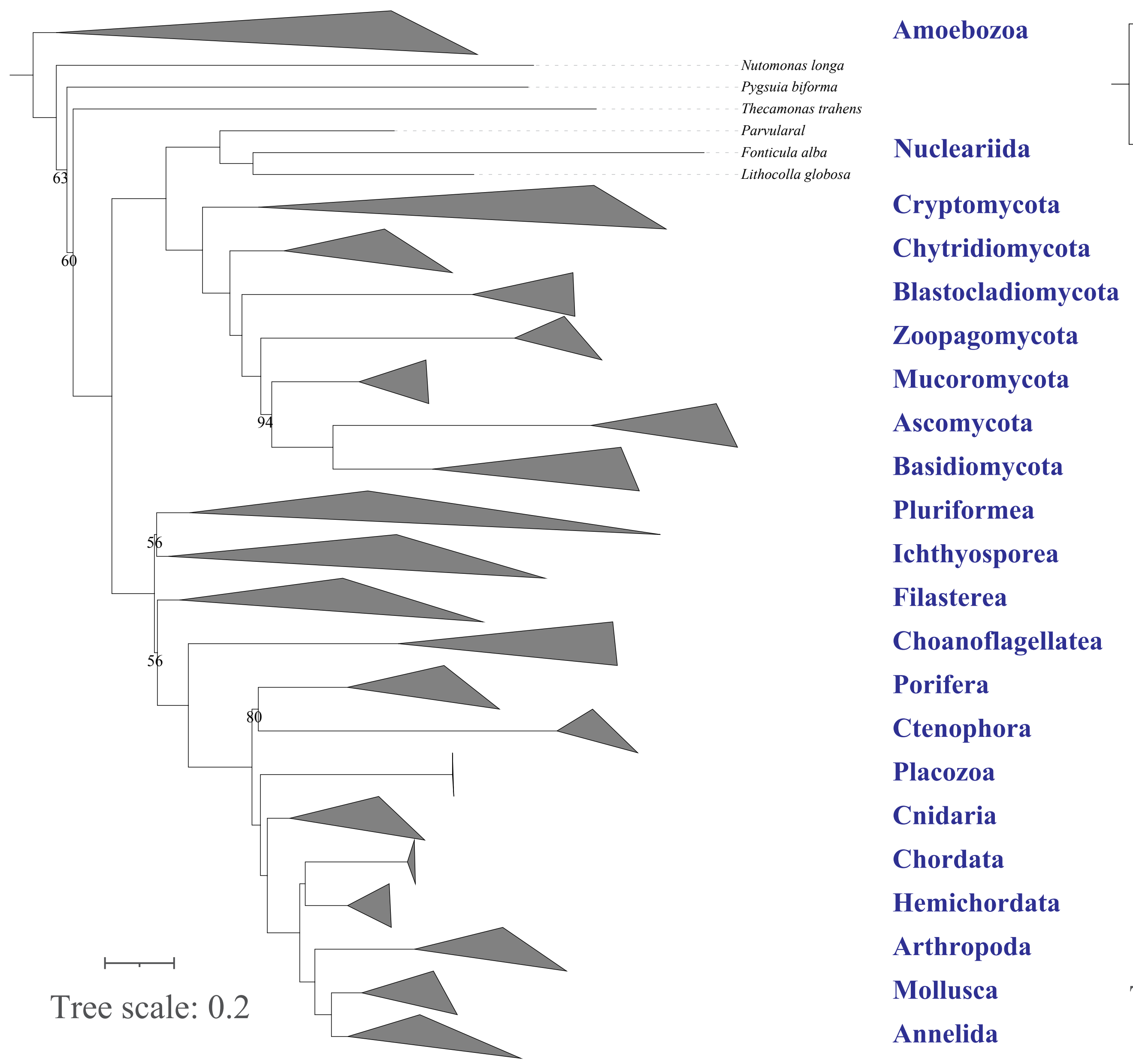

Amoebozoa
Nutomonas longa
Pygsuia biforma
Thecamonas trahens
Parvularal
Fonticula alba
Lithocolla globosa
Nucleariida
Cryptomycota
Chytridiomycota
Blastocladiomycota
Zoopagomycota
Mucoromycota
Ascomycota
Basidiomycota
Pluriformea
Ichthyosporea
Filasterea
Choanoflagellatea
Porifera
Ctenophora
Placozoa
Cnidaria
Chordata
Hemichordata
Arthropoda
Mollusca
Annelida
Tree scale: 0.2

59

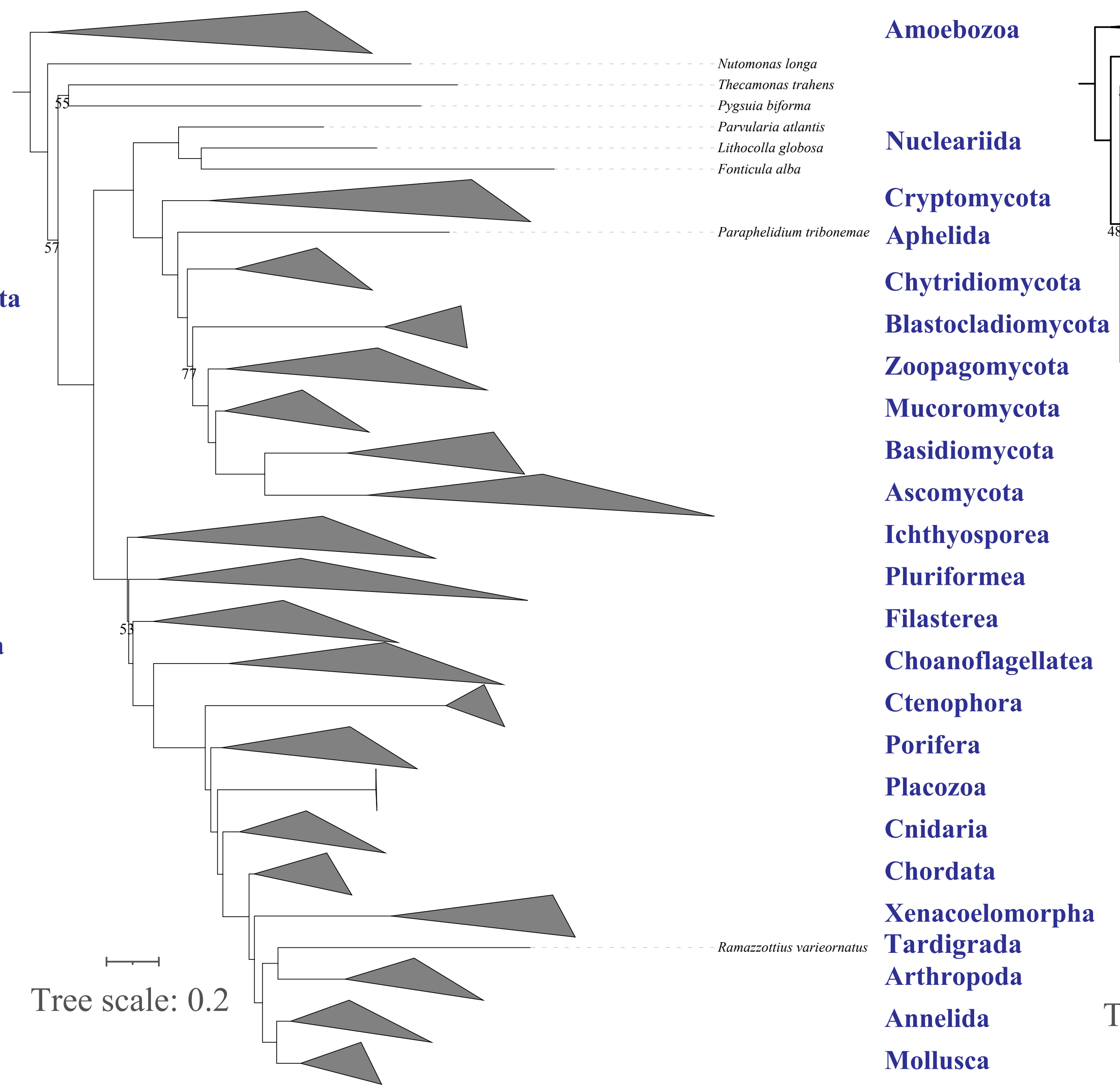

Amoebozoa
Nutomonas longa
Thecamonas trahens
Pygsuia biforma
Parvularia atlantis
Lithocolla globosa
Fonticula alba
Nucleariida
Cryptomycota
Paraphelidium tribonemae
Aphelida
Chytridiomycota
Blastocladiomycota
Zoopagomycota
Mucoromycota
Basidiomycota
Ascomycota
Ichthyosporea
Pluriformea
Filasterea
Choanoflagellatea
Ctenophora
Porifera
Placozoa
Cnidaria
Chordata
Xenacoelomorpha
Ramazzottius varieornatus
Tardigrada
Arthropoda
Annelida
Mollusca
Tree scale: 0.2

60

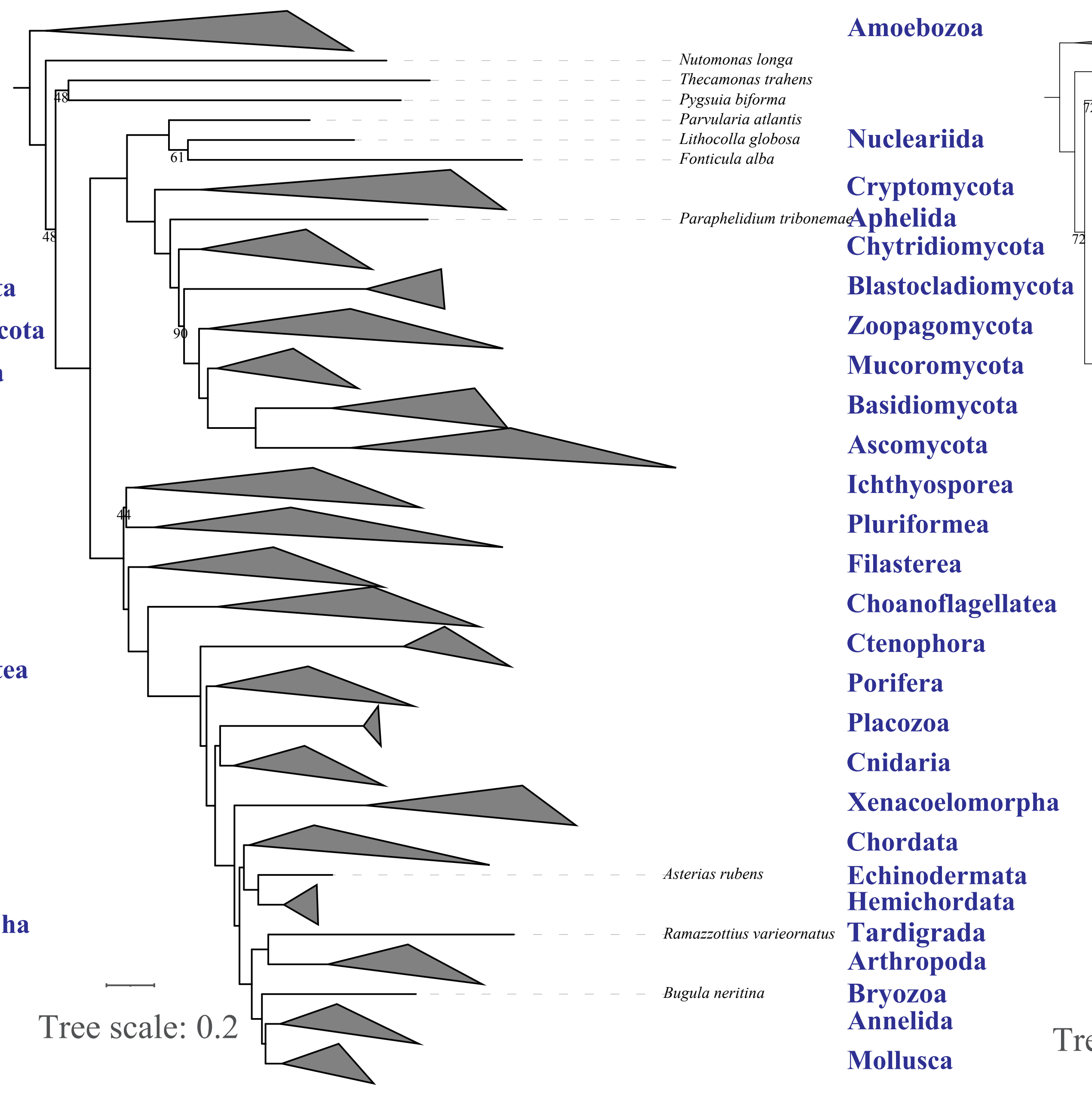

Amoebozoa
Nutomonas longa
Thecamonas trahens
Pygsuia biforma
Parvularia atlantis
Lithocolla globosa
Fonticula alba
Nucleariida
Cryptomycota
Paraphelidium tribonemae
Aphelida
Chytridiomycota
Blastocladiomycota
Zoopagomycota
Mucoromycota
Basidiomycota
Ascomycota
Ichthyosporea
Pluriformea
Filasterea
Choanoflagellatea
Ctenophora
Porifera
Placozoa
Cnidaria
Xenacoelomorpha
Chordata
Asterias rubens
Echinodermata
Hemichordata
Ramazzottius varieornatus
Tardigrada
Arthropoda
Bugula neritina
Bryozoa
Annelida
Mollusca
Tree scale: 0.2

61

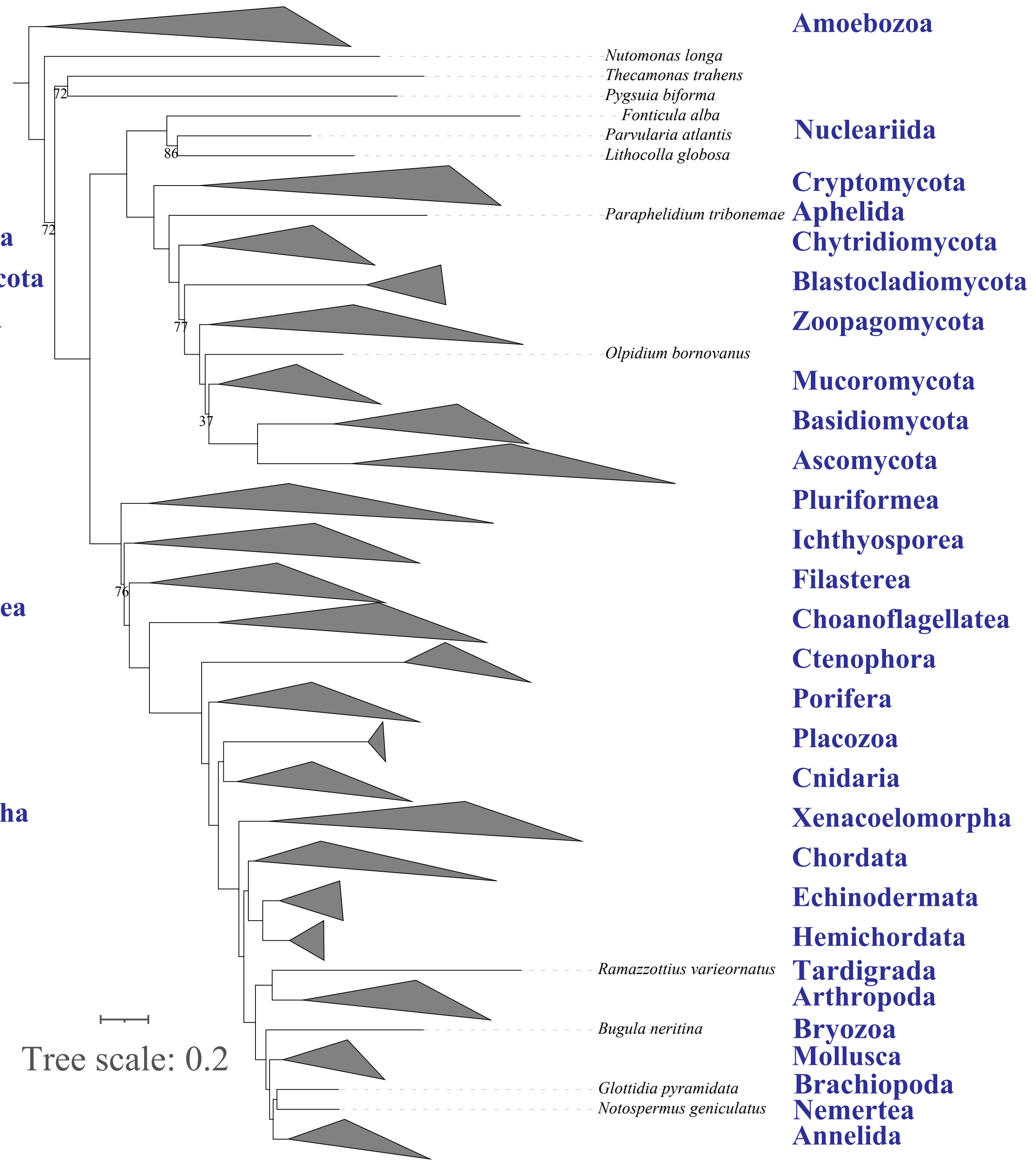

Amoebozoa
Nutomonas longa
Thecamonas trahens
Pygsuia biforma
Fonticula alba
Parvularia atlantis
Lithocolla globosa
Nucleariida
Cryptomycota
Paraphelidium tribonemae
Aphelida
Chytridiomycota
Blastocladiomycota
Zoopagomycota
Olpidium bornovanus
Mucoromycota
Basidiomycota
Ascomycota
Pluriformea
Ichthyosporea
Filasterea
Choanoflagellatea
Ctenophora
Porifera
Placozoa
Cnidaria
Xenacoelomorpha
Chordata
Echinodermata
Hemichordata
Ramazzottius varieornatus
Tardigrada
Arthropoda
Bugula neritina
Bryozoa
Mollusca
Glottidia pyramidata
Brachiopoda
Notospermus geniculatus
Nemertea
Annelida
Tree scale: 0.2

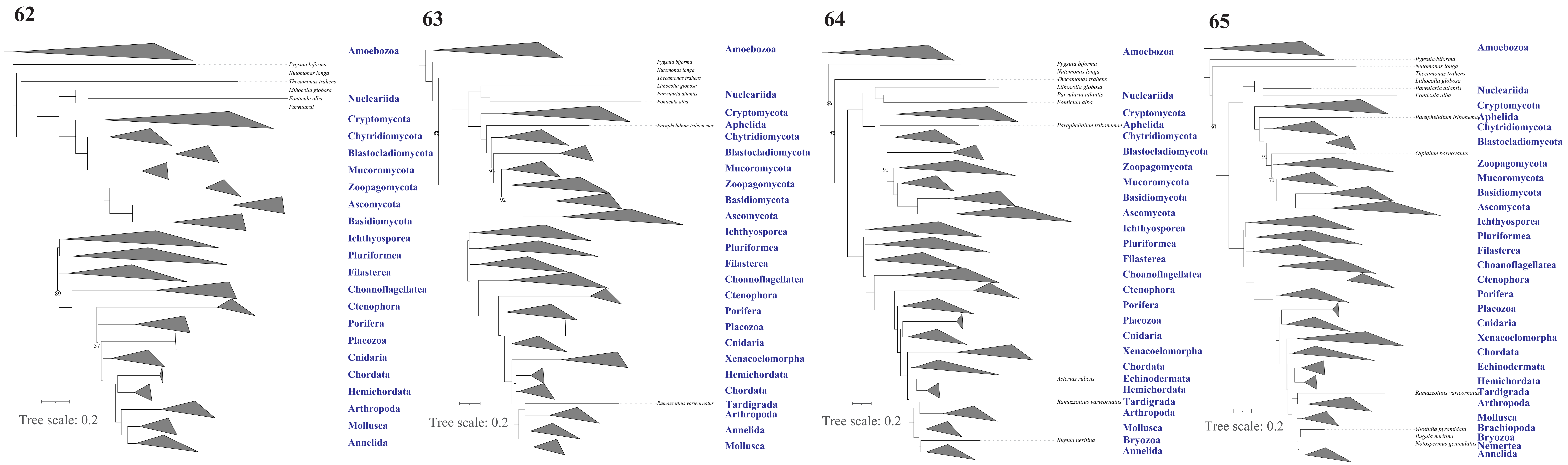

62
Pygsuia biforma
Nutomonas longa
Thecamonas trahens
Lithocolla globosa
Fonticula alba
Parvularal
89
57
Tree scale: 0.2
Amoebozoa
Nucleariida
Cryptomycota
Chytridiomycota
Blastocladiomycota
Mucoromycota
Zoopagomycota
Ascomycota
Basidiomycota
Ichthyosporea
Pluriformea
Filasterea
Choanoflagellatea
Ctenophora
Porifera
Placozoa
Cnidaria
Chordata
Hemichordata
Arthropoda
Mollusca
Annelida
63
Pygsuia biforma
Nutomonas longa
Thecamonas trahens
Lithocolla globosa
Parvularia atlantis
Fonticula alba
Paraphelidium tribonemae
Ramazzottius varieornatus
89
93
92
Tree scale: 0.2
Amoebozoa
Nucleariida
Cryptomycota
Aphelida
Chytridiomycota
Blastocladiomycota
Mucoromycota
Zoopagomycota
Basidiomycota
Ascomycota
Ichthyosporea
Pluriformea
Filasterea
Choanoflagellatea
Ctenophora
Porifera
Placozoa
Cnidaria
Xenacoelomorpha
Hemichordata
Chordata
Tardigrada
Arthropoda
Annelida
Mollusca
64
Pygsuia biforma
Nutomonas longa
Thecamonas trahens
Lithocolla globosa
Parvularia atlantis
Fonticula alba
Paraphelidium tribonemae
Asterias rubens
Ramazzottius varieornatus
Bugula neritina
89
79
91
Tree scale: 0.2
Amoebozoa
Nucleariida
Cryptomycota
Aphelida
Chytridiomycota
Blastocladiomycota
Zoopagomycota
Mucoromycota
Basidiomycota
Ascomycota
Ichthyosporea
Pluriformea
Filasterea
Choanoflagellatea
Ctenophora
Porifera
Placozoa
Cnidaria
Xenacoelomorpha
Chordata
Echinodermata
Hemichordata
Tardigrada
Arthropoda
Mollusca
Bryozoa
Annelida
65
Pygsuia biforma
Nutomonas longa
Thecamonas trahens
Lithocolla globosa
Parvularia atlantis
Fonticula alba
Paraphelidium tribonemae
Olpidium bornovanus
Ramazzottius varieornatus
Glottidia pyramidata
Bugula neritina
Notospermus geniculatus
93
91
71
Tree scale: 0.2
Amoebozoa
Nucleariida
Cryptomycota
Aphelida
Chytridiomycota
Blastocladiomycota
Zoopagomycota
Mucoromycota
Basidiomycota
Ascomycota
Ichthyosporea
Pluriformea
Filasterea
Choanoflagellatea
Ctenophora
Porifera
Placozoa
Cnidaria
Xenacoelomorpha
Chordata
Echinodermata
Hemichordata
Tardigrada
Arthropoda
Mollusca
Brachiopoda
Bryozoa
Nemertea
Annelida

66

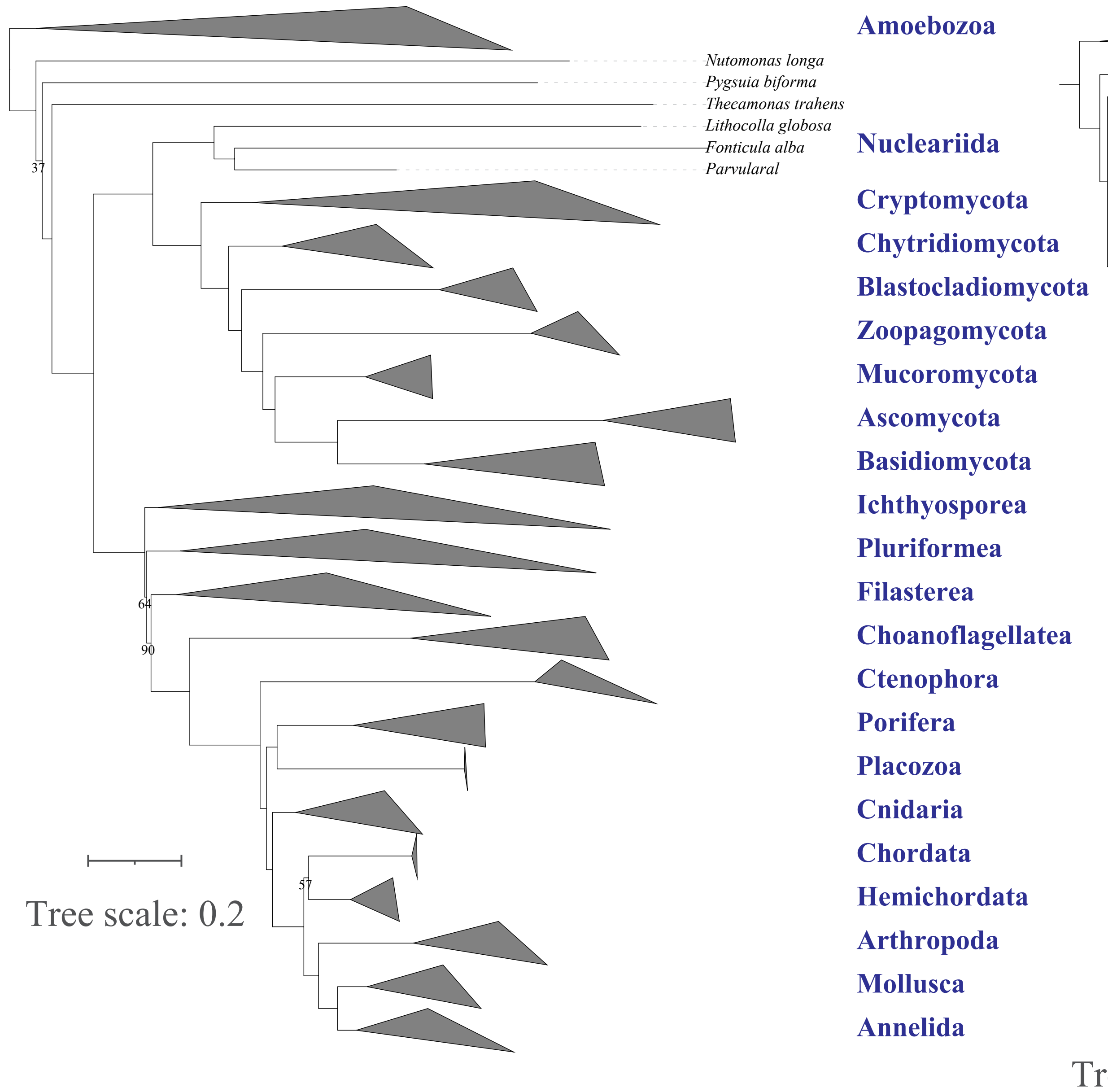
Amoebozoa
Nutomonas longa
Pygsuia biforma
Thecamonas trahens
Lithocolla globosa
Fonticula alba
Parvularal
37
Nucleariida
Cryptomycota
Chytridiomycota
Blastocladiomycota
Zoopagomycota
Mucoromycota
Ascomycota
Basidiomycota
Ichthyosporea
Pluriformea
Filasterea
Choanoflagellatea
Ctenophora
Porifera
Placozoa
Cnidaria
Chordata
Hemichordata
Arthropoda
Mollusca
Annelida
64
90
57
Tree scale: 0.2

67

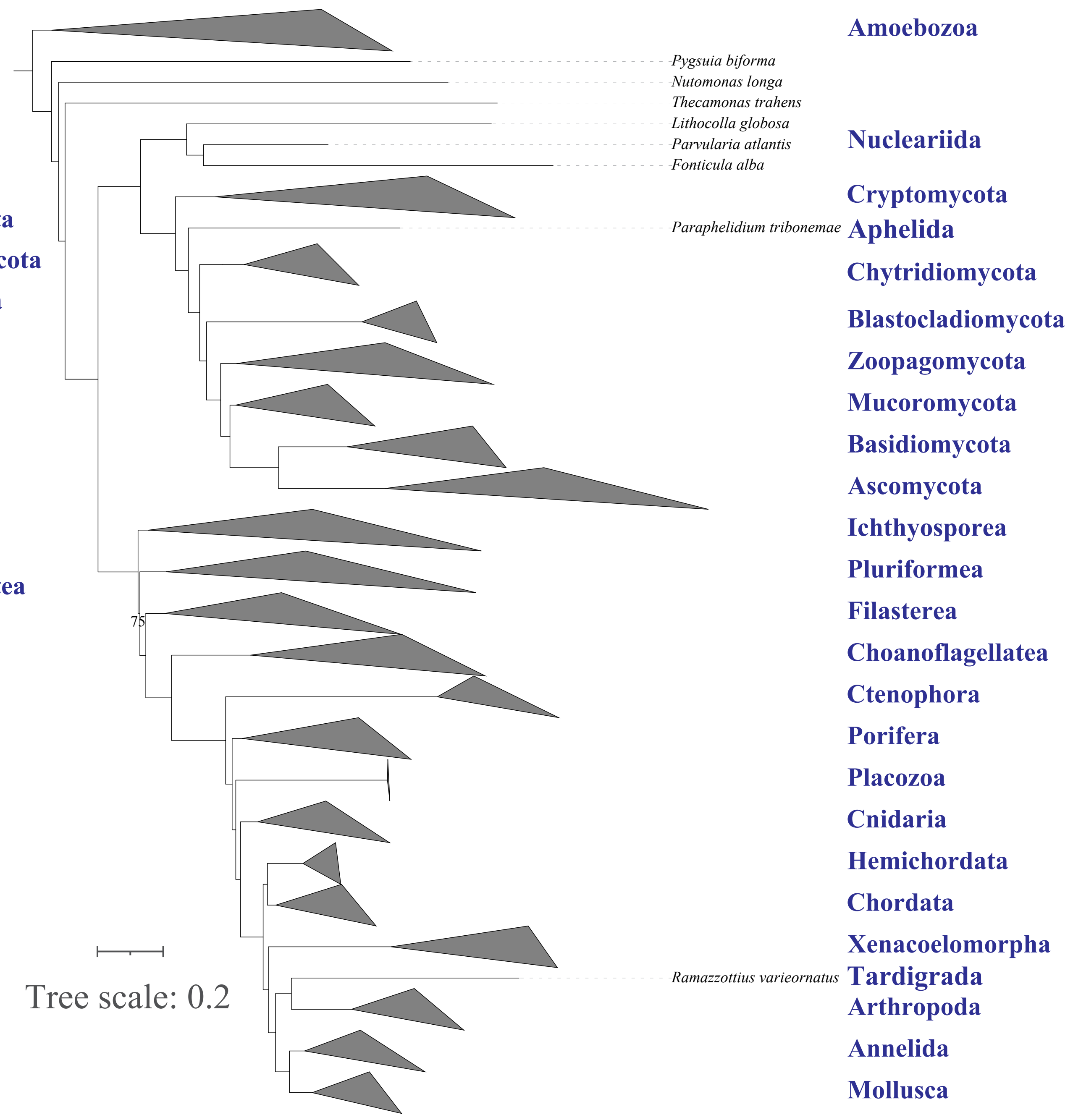
Amoebozoa
Pygsuia biforma
Nutomonas longa
Thecamonas trahens
Lithocolla globosa
Parvularia atlantis
Fonticula alba
Nucleariida
Cryptomycota
Paraphelidium tribonemae
Aphelida
Chytridiomycota
Blastocladiomycota
Zoopagomycota
Mucoromycota
Basidiomycota
Ascomycota
Ichthyosporea
Pluriformea
Filasterea
Choanoflagellatea
Ctenophora
Porifera
Placozoa
Cnidaria
Hemichordata
Chordata
Xenacoelomorpha
Ramazzottius varieornatus
Tardigrada
Arthropoda
Annelida
Mollusca
75
Tree scale: 0.2

68

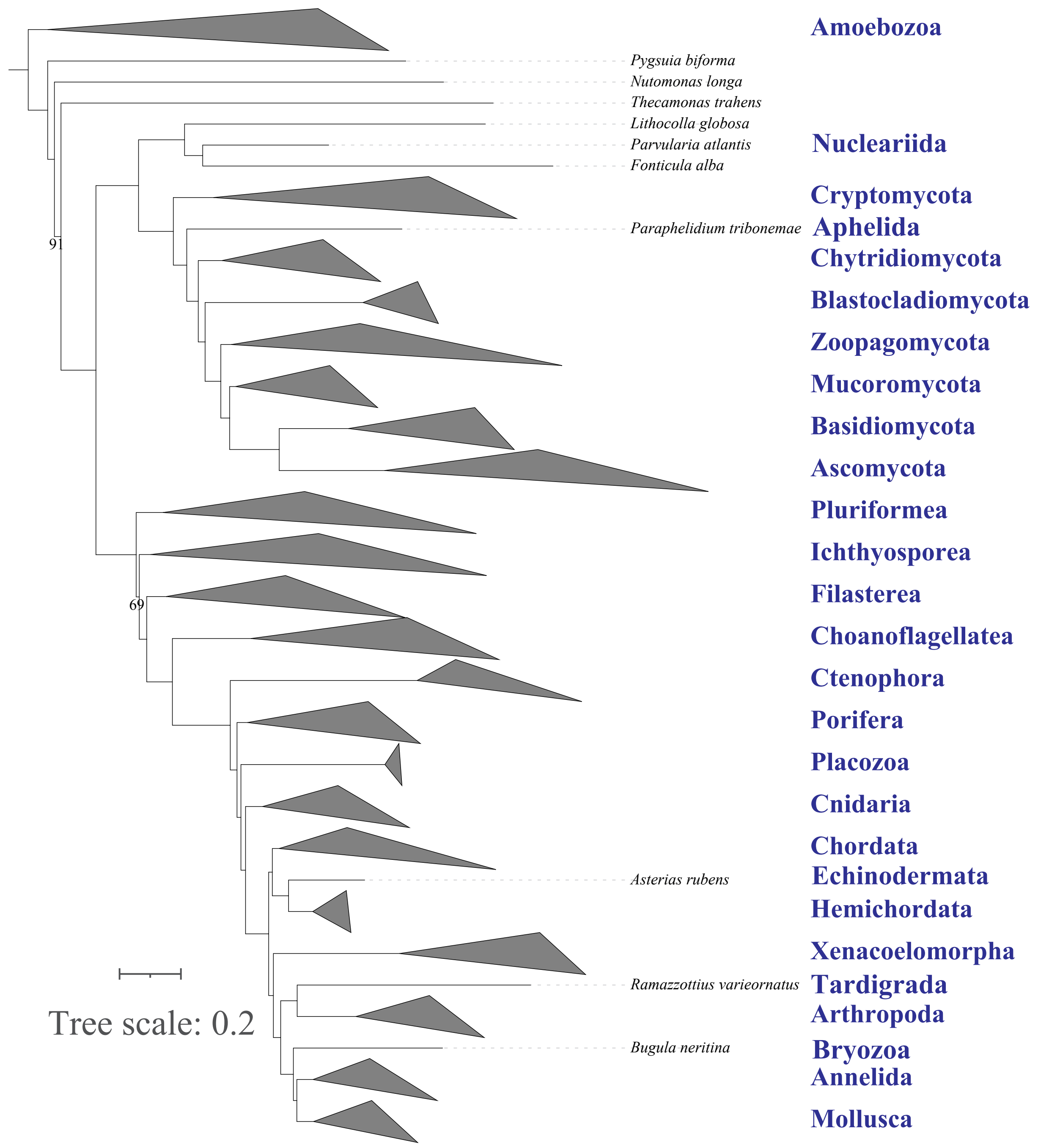
Amoebozoa
Pygsuia biforma
Nutomonas longa
Thecamonas trahens
Lithocolla globosa
Parvularia atlantis
Fonticula alba
Nucleariida
Cryptomycota
Paraphelidium tribonemae
Aphelida
Chytridiomycota
Blastocladiomycota
Zoopagomycota
Mucoromycota
Basidiomycota
Ascomycota
Pluriformea
Ichthyosporea
Filasterea
Choanoflagellatea
Ctenophora
Porifera
Placozoa
Cnidaria
Chordata
Asterias rubens
Echinodermata
Hemichordata
Xenacoelomorpha
Ramazzottius varieornatus
Tardigrada
Arthropoda
Bugula neritina
Bryozoa
Annelida
Mollusca
91
69
Tree scale: 0.2

69

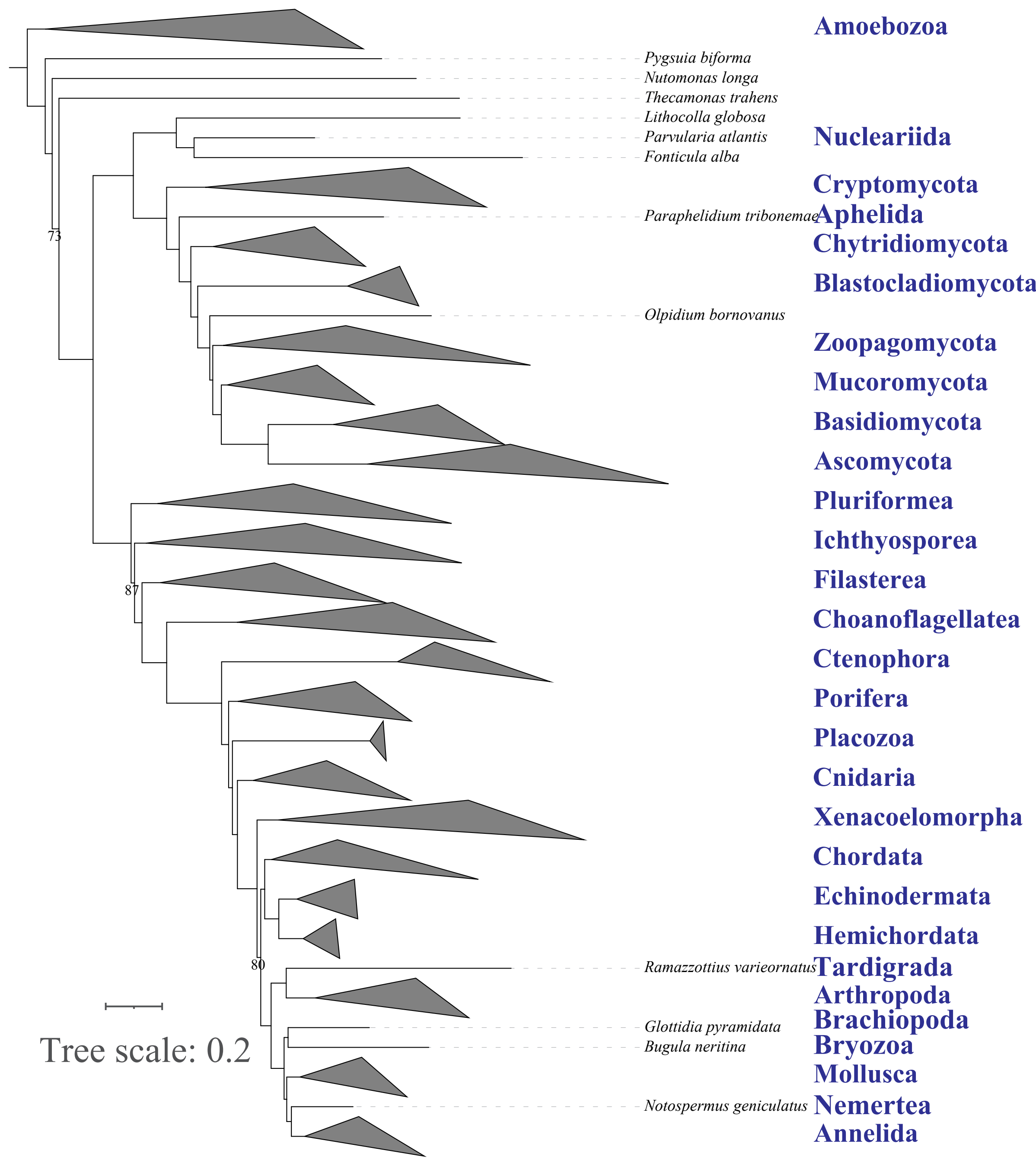
Amoebozoa
Pygsuia biforma
Nutomonas longa
Thecamonas trahens
Lithocolla globosa
Parvularia atlantis
Fonticula alba
Nucleariida
Cryptomycota
Paraphelidium tribonemae
Aphelida
Chytridiomycota
Blastocladiomycota
Olpidium bornovanus
Zoopagomycota
Mucoromycota
Basidiomycota
Ascomycota
Pluriformea
Ichthyosporea
Filasterea
Choanoflagellatea
Ctenophora
Porifera
Placozoa
Cnidaria
Xenacoelomorpha
Chordata
Echinodermata
Hemichordata
Ramazzottius varieornatus
Tardigrada
Arthropoda
Glottidia pyramidata
Brachiopoda
Bugula neritina
Bryozoa
Mollusca
Notospermus geniculatus
Nemertea
Annelida
73
87
80
Tree scale: 0.2

70

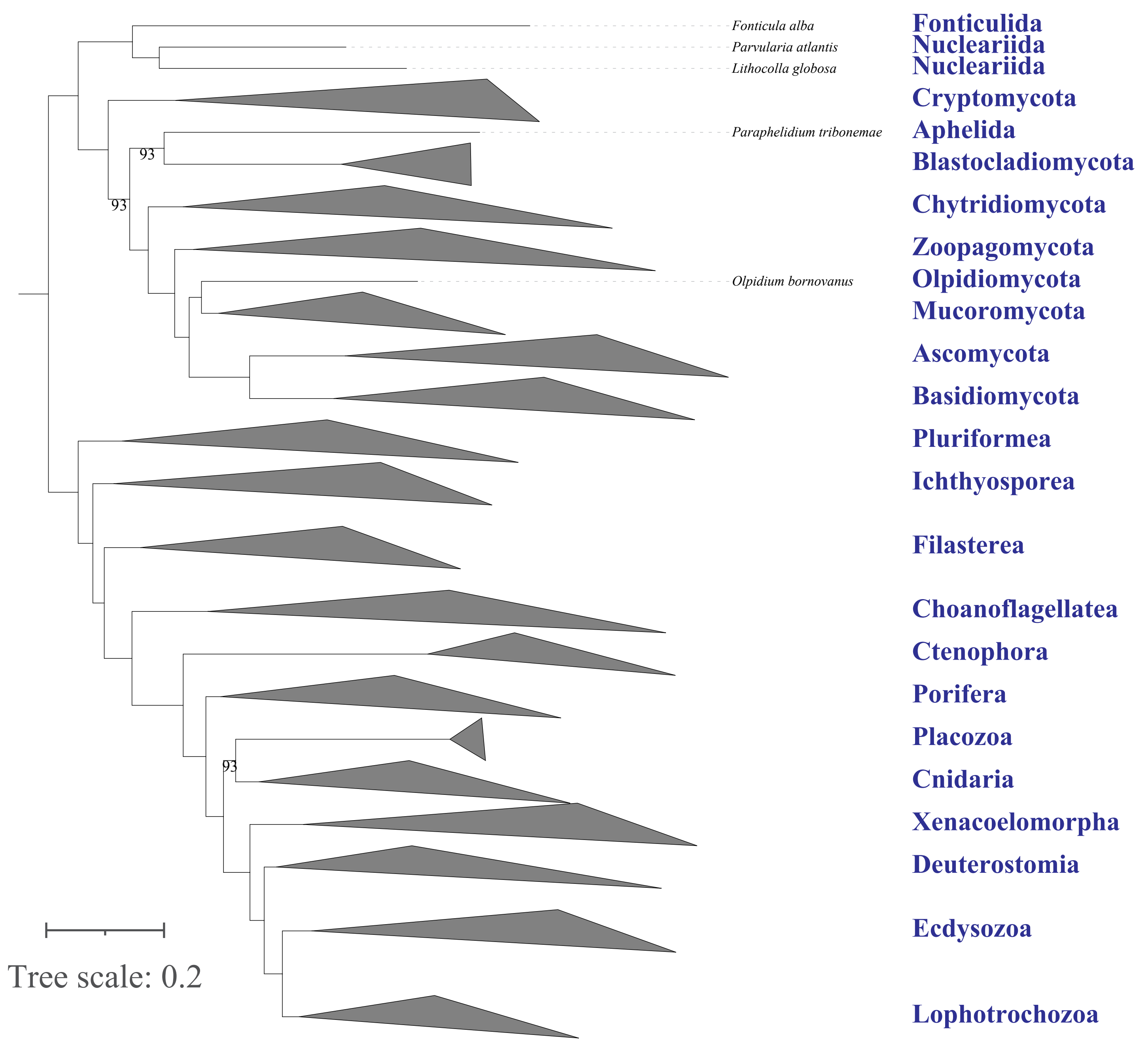

Fonticula alba
Parvularia atlantis
Lithocolla globosa
Paraphelidium tribonemae
Olpidium bornovanus
Fonticulida
Nucleariida
Nucleariida
Cryptomycota
Aphelida
Blastocladiomycota
Chytridiomycota
Zoopagomycota
Olpidiomycota
Mucoromycota
Ascomycota
Basidiomycota
Pluriformea
Ichthyosporea
Filasterea
Choanoflagellatea
Ctenophora
Porifera
Placozoa
Cnidaria
Xenacoelomorpha
Deuterostomia
Ecdysozoa
Lophotrochozoa
93
93
93
Tree scale: 0.2

71

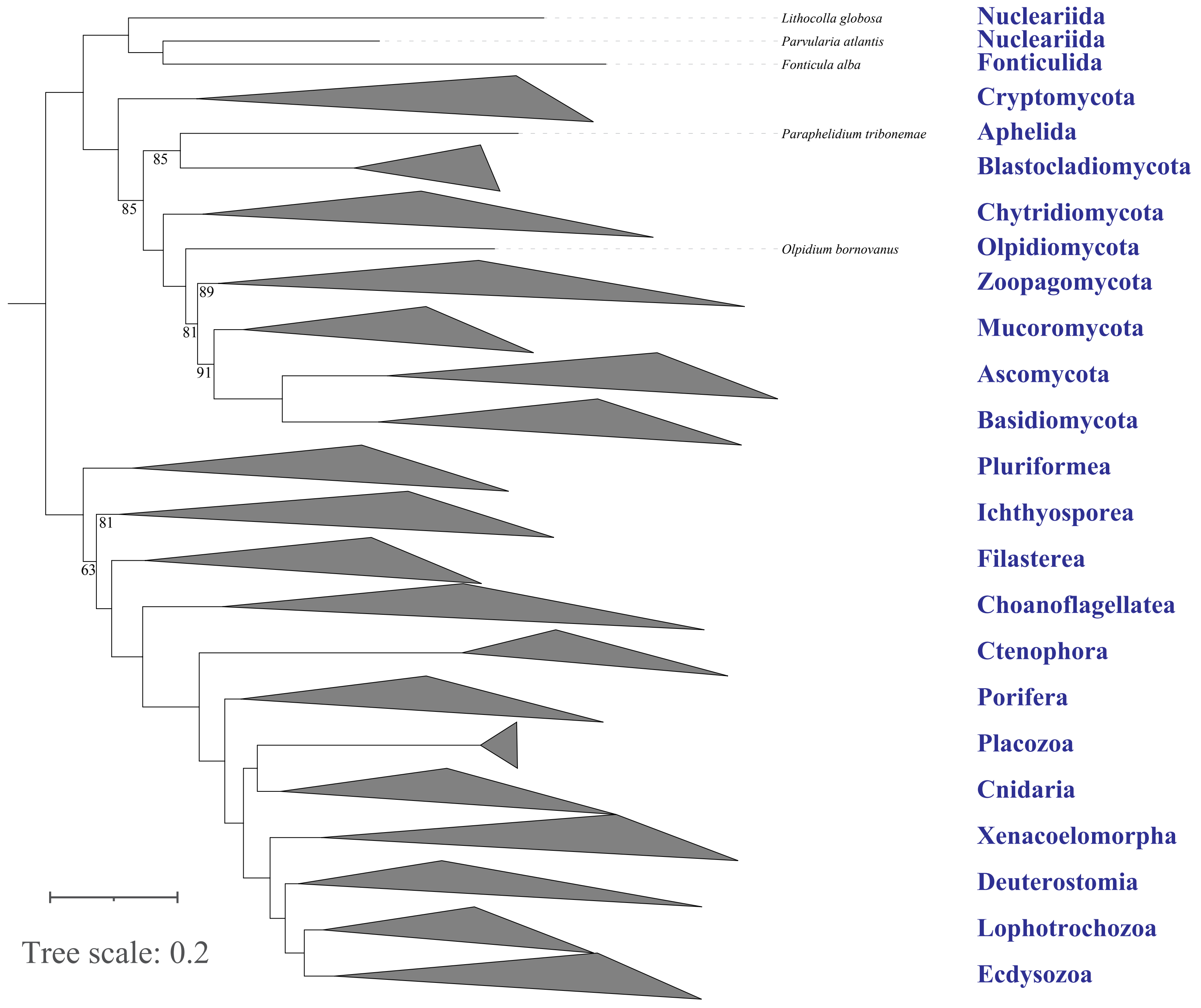

Lithocolla globosa
Parvularia atlantis
Fonticula alba
Paraphelidium tribonemae
Olpidium bornovanus
Nucleariida
Nucleariida
Fonticulida
Cryptomycota
Aphelida
Blastocladiomycota
Chytridiomycota
Olpidiomycota
Zoopagomycota
Mucoromycota
Ascomycota
Basidiomycota
Pluriformea
Ichthyosporea
Filasterea
Choanoflagellatea
Ctenophora
Porifera
Placozoa
Cnidaria
Xenacoelomorpha
Deuterostomia
Lophotrochozoa
Ecdysozoa
85
85
89
81
91
81
63
Tree scale: 0.2

72

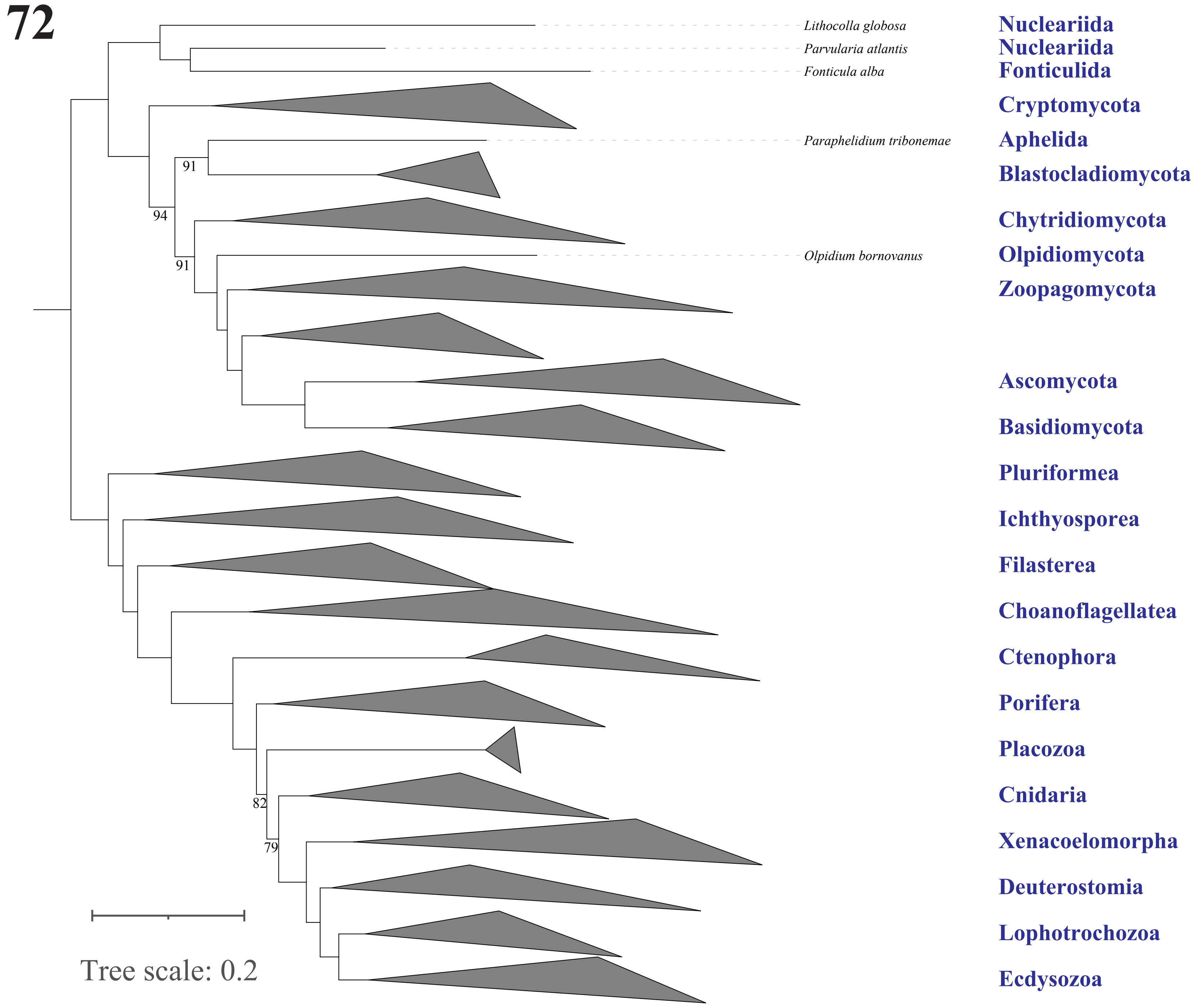

Lithocolla globosa
Parvularia atlantis
Fonticula alba
Paraphelidium tribonemae
Olpidium bornovanus
Nucleariida
Nucleariida
Fonticulida
Cryptomycota
Aphelida
Blastocladiomycota
Chytridiomycota
Olpidiomycota
Zoopagomycota
Ascomycota
Basidiomycota
Pluriformea
Ichthyosporea
Filasterea
Choanoflagellatea
Ctenophora
Porifera
Placozoa
Cnidaria
Xenacoelomorpha
Deuterostomia
Lophotrochozoa
Ecdysozoa
91
94
91
82
79
Tree scale: 0.2

72

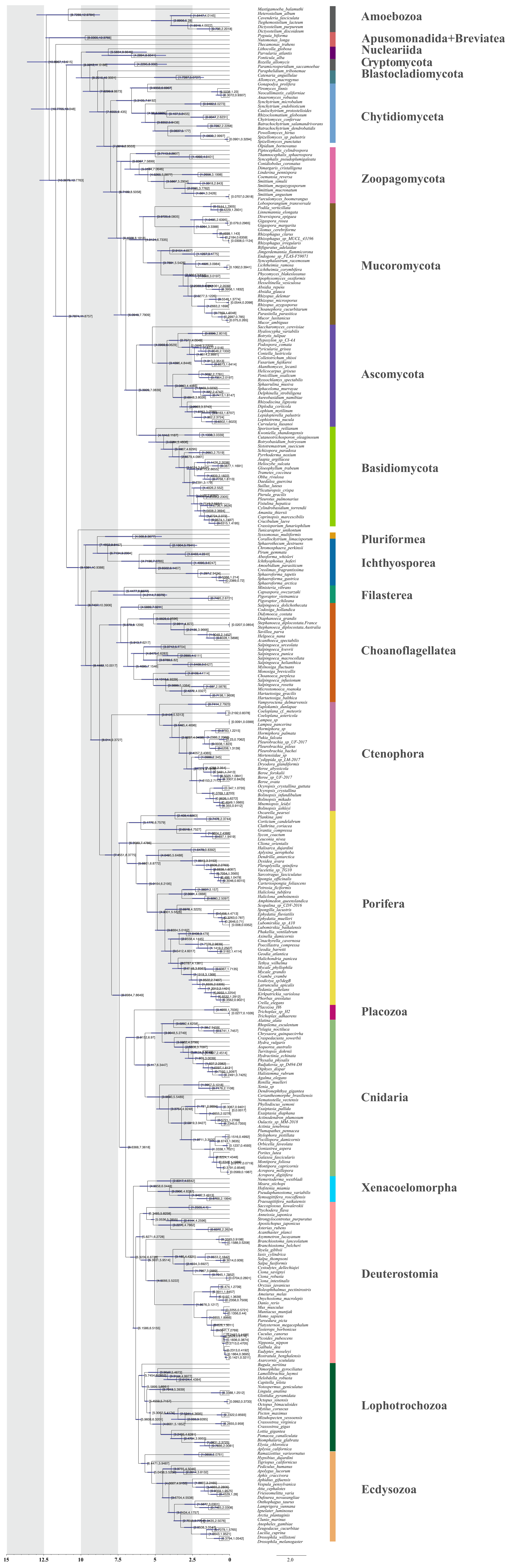
Amoebozoa
Apusomonadida+Breviatea
Nucleariida
Cryptomycota
Blastocladiomycota
Chytidiomyceta
Zoopagomycota
Mucoromycota
Ascomycota
Basidiomycota
Pluriformea
Ichthyosporea
Filasterea
Choanoflagellatea
Ctenophora
Porifera
Placozoa
Cnidaria
Xenacoelomorpha
Deuterostomia
Lophotrochozoa
Ecdysozoa
15
12.5
10
7.5
5
2.5
0
2.0

73

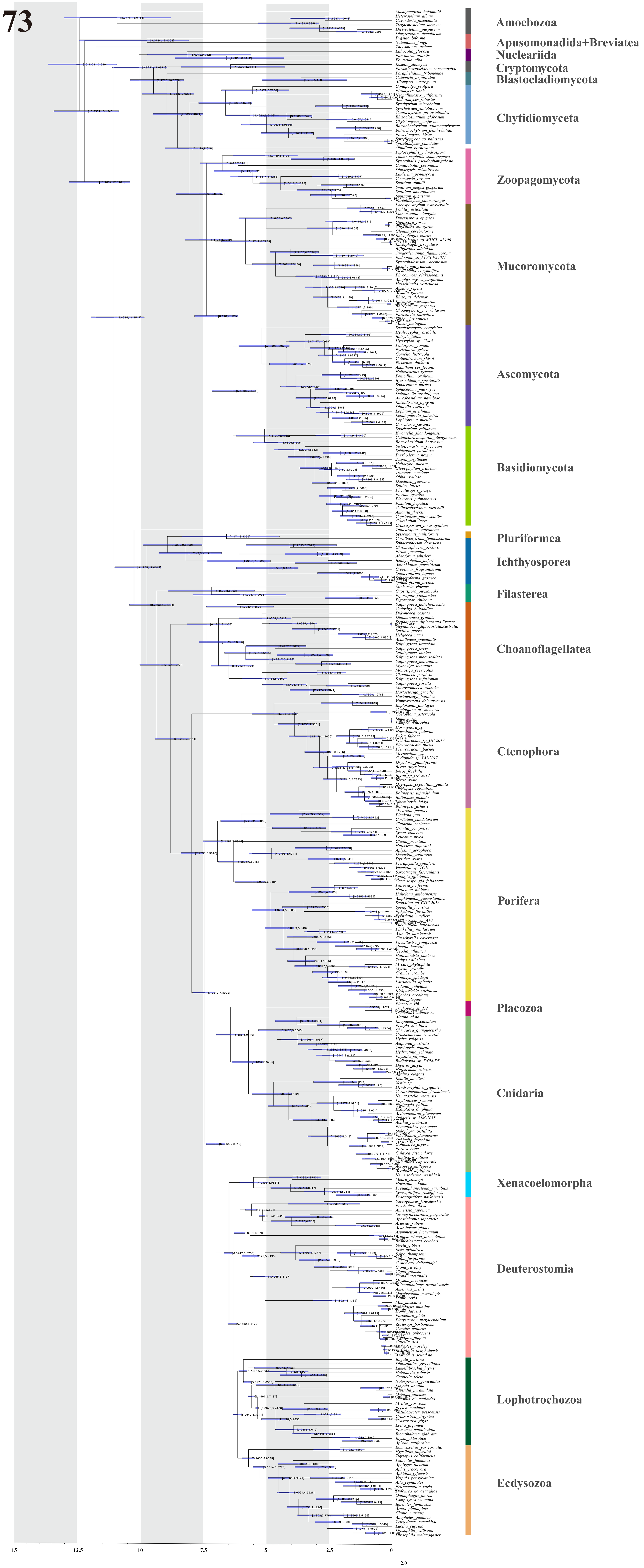
Amoebozoa
Apusomonadida+Breviatea
Nucleariida
Cryptomycota
Blastocladiomycota
Chytidiomyceta
Zoopagomycota
Mucoromycota
Ascomycota
Basidiomycota
Pluriformea
Ichthyosporea
Filasterea
Choanoflagellatea
Ctenophora
Porifera
Placozoa
Cnidaria
Xenacoelomorpha
Deuterostomia
Lophotrochozoa
Ecdysozoa
15
12.5
10
7.5
5
2.5
0
2.0
